# Intrinsic Disorder in the Host Proteins Entrapped in Rabies Virus Particles

**DOI:** 10.3390/v16060916

**Published:** 2024-06-04

**Authors:** Hafiza Nimra Ashraf, Vladimir N. Uversky

**Affiliations:** 1 Department of Molecular Medicine, Morsani College of Medicine, University of South Florida, Tampa, FL 33612, USA; ashraf12@usf.edu; 2 USF Health Byrd Alzheimer’s Research Institute, Morsani College of Medicine, University of South Florida, Tampa, FL 33612, USA

**Keywords:** rabies virus, intrinsically disordered proteins, protein–protein interactions, neuromodulin, Chmp4b, DnaJB6, Vps37B, Wasl

## Abstract

A proteomics analysis of purified rabies virus (RABV) revealed 47 entrapped host proteins within the viral particles. Out of these, 11 proteins were highly disordered. Our study was particularly focused on five of the RABV-entrapped mouse proteins with the highest levels of disorder: Neuromodulin, Chmp4b, DnaJB6, Vps37B, and Wasl. We extensively utilized bioinformatics tools, such as FuzDrop, D^2^P^2^, UniProt, RIDAO, STRING, AlphaFold, and ELM, for a comprehensive analysis of the intrinsic disorder propensity of these proteins. Our analysis suggested that these disordered host proteins might play a significant role in facilitating the rabies virus pathogenicity, immune system evasion, and the development of antiviral drug resistance. Our study highlighted the complex interaction of the virus with its host, with a focus on how the intrinsic disorder can play a crucial role in virus pathogenic processes, and suggested that these intrinsically disordered proteins (IDPs) and disorder-related host interactions can also be a potential target for therapeutic strategies.

## 1. Introduction

The rabies virus (RABV), also known as Rhabdovirus, causes rabies, which is a preventable (through the prompt administration of post-exposure prophylaxis (PEP) to victims of bites by rabid animals [1]) but rarely curable disease [2]. Once the symptoms start manifesting, the disease is nearly 100% fatal [3]. It was reported that an RABV infection causes more than 55,000 deaths worldwide [4].

The rabies virus affects the central nervous system, causing acute infection [5]. The transmission of the virus usually happens through the bite of a rabid animal [2,3]. The virus has a rod- or bullet-like shape, and its genome is a single-stranded, negative-sense, linear non-segmented enveloped RNA [6]. The RABV belongs to the Rhabdoviridae family and genus Lyssavirus, hence, the name rhabdovirus [6,7].

The genome encodes for five different proteins named N (nucleoprotein), P (phosphoprotein), M (matrix protein), G (glycoprotein), and L (polymerase) [6]. The bullet-shaped virus is enclosed in a lipid envelope covered by glycoproteins that facilitates the attachment of the virus to the host cell receptors and thus ensures viral entry. The helical ribonucleocapsid core is composed of the viral genome and nucleoprotein [8].

Most often, exposure to the RABV happens due to the bite or scratches of a rabid animal [2,6]. At the site of injury, the muscle cells of the new host become exposed to the rabid animal saliva, which contains the particles of the rabies virus [9,10]. The RABV initially replicates in the muscle cells, but its next destination is the peripheral nervous system [6,9,10]. The virus binds to the receptors on the nerve endings of the peripheral nervous system near the site of infection [11,12]. From here on, the RABV moves along the nerves through axonal transport to enter the peripheral nervous system [11]. Then, it moves to the main target, the central nervous system [2]. When the RABV is in the central nervous system of the host, it starts to replicate rapidly, spreading to the spinal cord and different parts of the brain, causing inflammation of the brain (encephalitis) [2].

The lifecycle of the rabies virus as it enters the host cell can be divided into the following steps:

- Attachment/adsorption: At first, glycoprotein G of the virus interacts with the specific cell surface receptors [11];
- Endocytosis/penetration: Then, the virus enters the host cell through receptor-mediated endocytosis [6,11];
- Fusion: Upon binding of the glycoprotein to a host cell receptor, the pH-triggered fusion between the viral and host membranes is mediated [13,14];
- Uncoating (envelope removal): The fusion of the viral and endosomal membranes leads to the release of the viral ribonucleoprotein (RNP) complex into the cytoplasm [6,11];
- As the viral genome is tightly encapsidated by the viral nucleoprotein N, phosphoprotein P, and large protein L (or RNA-dependent RNA polymerase (RdRp)), upon its release into the cytoplasm, this RNP acts as the template for the transcription and replication processes catalyzed by the L-P polymerase complex [15];
- Negri body (inclusion body or viral factory) formation: An RABV infection induces the formation of cytoplasmic inclusion bodies (Negri bodies [16]), the biogenesis of which is driven by liquid–liquid phase separation [17,18], which serve as viral factories, i.e., functional structures, where viral transcription and replication take place [15];
- Transcription (or primary transcription): Since the genome of the RABV represents a linear, single-negative-stranded RNA, a viral-encoded RdRp (L protein) transcribes the viral antigenome RNA to mRNA in the cytoplasm [6,11]. Transcription leads to the synthesis of a positive-stranded leader RNA and five monocistronic capped and polyadenylated mRNAs;
- Translation: A viral mRNA strand is used for the translation of five major proteins (N, P, M, G, and L);
- Replication: RdRp replicates the progeny genome through a complementary replicative intermediate, the antigenome [6,11]. Here, “the RABV RdRp ignores the signals for mRNA synthesis on the genome to copy it into the positive-strand antigenome” [19]. After its antigenome is assembled into the RNP complex via its association with N, this replicative intermediate antigenome acts as a template for further rounds of replication to generate genomic RNA for progeny virions (antigenome is always always encapsidated by the N protein). Replication requires the newly synthesized N, P, and L proteins and a set of host factors;
- Secondary transcription: New rounds of transcription (secondary transcription), translation, and replication take place following primary replication;
- Assembly: All these viral particles (genome and proteins) assemble into new virions [11];
- Budding: Assembled virions bud off from the cell surfaces of host cells, acquiring their envelope from the host cell membrane [20];
- Release: The mature rabies virus normally releases from the cells through cell lysis and spreads through the central nervous system and brain to infect healthy cells [20].

During the assembly of viral progeny, some host proteins become integrated into the mature virion particles, which may help the virus to camouflage as host cells to escape the immune system [21]. In this article, we will focus on the analysis of the intrinsic disorder of such host proteins entrapped in the virus particles. Knowing more about the intrinsic disorder properties of these proteins will help us understand the interactions of viruses with host cells, because intrinsically disordered proteins (IDPs) and intrinsically disordered regions (IDRs) are highly flexible and can change their structure and function in response to different environments [22]. Therefore, intrinsic disorder of proteins can help viruses to become more adaptable and flexible. We can also learn the strategies of viruses in evading the immune system to help us understand the pathogenesis of the rabies virus in greater depth.

In this context, Yan Zhang and colleagues published a paper discussing the host proteins that are incorporated into RABV particles when they are released from the host cells [23]. The authors purified the viral particles to perform the proteome profiling of the RABV. They found out that along with 5 main viral proteins, 49 host proteins are also integrated into viral particles, and 24 of these directly take part in viral replication, suggesting that the virus hijacks the host cellular machinery and interacts with host proteins for efficient replication [23]. An illustrative example is given by the integration of heat shock protein (HSP70) into a matured RABV virion. Decreasing the expression of HSP70 leads to a substantial reduction in the levels of viral RNAs, proteins, and virions [24]. This suggests that the enveloped viruses utilized the host proteins specifically to carry out their replication [23].

Rabies viruses that belong to the Rhabdoviruses family bud out of host cells using the host endosomal sorting complex required for transport (ESCRT) machinery [25,26]. The hijacking of the host ESCRT machinery plays a vital role in integrating the host proteins into the virus particles [25,26]. Two important proteins in this respect are charged multivesicular body protein 4b (Chmp4b) and Vacuolar protein sorting-associated protein 37B (Vps37b); both play crucial roles in the budding process during the virus life cycle [23]. Chmp4b is an essential component of ESCRT III complex, which is responsible for the final stages of budding [23]. Thus, the protein is involved in the final detachment of the newly formed virions from the cell membrane of the host cells. On the other hand, Vps37b is involved with ESCRT I and takes part in the initial step of the viral budding process [23]. Therefore, these two proteins can serve as potential therapeutic targets.

The protocol utilized by Zhang and colleagues in this important study [23] is outlined in Supplementary Materials S1. Zhang et al. also performed a functional characterization of the 49 incorporated host proteins found in the virus particles through the gene ontology database [23]. They were aiming to achieve a deep understanding of the complex interaction of host cells and the RABV and the functional implications of these proteins in the virion particles, like the involvement in viral processes such as budding [23]. A protein–protein interaction network analysis was carried out, which also strongly suggests that many of these host proteins are involved in viral budding, especially through ESCRT machinery [23]. This implies the possibility that the virus might be exploiting these host proteins, mainly the ones involved in ESCRT machinery, to exit the host cells, further assisting the viral pathogenesis [23]. One important aspect was left unexplored by the authors, namely the intrinsic disorder status of the host proteins entrapped in RABV particles. 

Intrinsically disordered proteins (IDPs) are a class of biologically active proteins without unique structures [22,27]. Contrary to traditional ordered proteins, IDPs and intrinsically disordered regions (IDRs) lack well-defined, three-dimensional structures and exist as highly dynamic conformational ensembles [22,27]. Intrinsic disorder is highly prevalent, and almost 70% of PDB structures have disordered regions [28]. IDPs are multifunctional proteins that can have multiple binding partners and are characterized by their high sensitivity to subtle changes in local environmental conditions like the pH and temperature, being capable of rapid change of their structures in response to the external environment [22,27]. IDPs/IDRs have a large interface area with a dominance of hydrophobic–hydrophobic contact. Unlike ordered proteins, IDPs have a weak hydrophobic core (if any), as their amino acid sequences have a low content of hydrophobic and aromatic residues and contain large numbers of charged and polar residues [22,29]. All these properties make intrinsically disordered proteins an integral part of the protein universe, with important biological functions that complement the functionality of ordered proteins. The flexibility and adaptability of IPDs make them suitable candidates to take part in diverse cellular functions like cell signaling, molecular recognition, and protein–protein interactions [30]. At the same time, the adaptable and flexible nature of IDPs also makes them important players in the pathogenesis of various diseases like cancer and neurodegenerative diseases [31,32,33].

In this study, to analyze the intrinsic disorder status of the host proteins entrapped in the RABV, we used the data on the 47 high-confidence host proteins reported by Zhang et al. [23]. These entrapped proteins were subjected to a multifactorial disorder analysis using a set of commonly used disorder predictors. Then, we conducted a more detailed bioinformatics characterization of the five entrapped proteins with highest levels of predicted disorder. 

## 2. Materials and Methods

### 2.1. Protein Datasets

The UniProt IDs of all mouse proteins analyzed in this study were retrieved from Table 1 of the Zhang et al. research article [23]. These IDs were used to collect the amino acid sequences (in FASTA format) of these proteins from the UniProt database, which are listed in Supplementary File S1. We subjected all these proteins to a bioinformatics analysis and selected the most disordered proteins for in-depth research. The selected proteins are neuromodulin (also known as growth-associated protein 43 (Gap43), calmodulin-binding protein P-57, or axonal membrane protein GAP-43; UniProt ID: P06837), a charged multivesicular body protein 4b (Chmp4b, UniProt ID: Q9D8B3), Dnaj homolog superfamily B member 6 (Dnajb6, UniProt ID: O54946), a vacuolar protein sorting-associated protein 37B (Vps37b, UniProt ID; Q8R0J7), and a Neural Wiskott–Aldrich syndrome protein (also known as actin nucleation-promoting factor WASL; UniProt ID: Q91YD9). The analysis of proteins using various bioinformatics tools discussed below was performed by submitting their amino acid sequences in FASTA format to corresponding computational platforms.

### 2.2. Exploration of the Intrinsic Disorder Predisposition

The susceptibility of our protein dataset to intrinsic disorder was evaluated through the RIDAO web platform, which is a convenient bioinformatics tool to generate the disorder profiles of query proteins. RIDAO combines the outputs of six commonly used per-residue disorder predictors, such as PONDR^®^ FIT, PONDR^®^ VSL2, PONDR^®^ VL3, PONDR^®^ VLXT, IUPred Short, and IUPred Long to generate the integral disorder profile of an individual query protein or to provide the global disorder characterization of a protein dataset [34]. The disorder score was assigned to each residue, with a residue with disorder score equal to or above 0.5 being considered as disordered and a residue with disorder score below 0.5 being predicted as ordered. Residues/regions with disorder scores between 0.15 and 0.5 were considered as ordered but flexible. For each protein, RIDAO also calculated the percent of predicted intrinsically disordered residues (PPIDRs), which was used for the classification of proteins as ordered (PPIDR < 10%), moderately disordered (10% ≤ PPIDR < 30%), and highly disordered (PPIDR ≥ 30%). 

In this and other studies conducted by our group, we utilize multiple disorder predictors mostly for illustrative purposes, i.e., to show the similarities and differences between different predictors in the per-residue disorder propensity plots generated for individual proteins. This is in line with the accepted practice in the field to use multiple tools, as they are sensitive to different disorder-related sequence features. On the other hand, while conducting global disorder predisposition analyses of various protein datasets, we are usually ranking proteins based on the PONDR^®^ VSL2 outputs, as the effectiveness and accuracy of this tool has been proven in the Critical Assessment of protein Intrinsic Disorder (CAID) [35]. In the second CAID round, PONDR^®^ VSL2 was not listed among the top 10 predictors, being ranked #20 and #18 based on the AUC (area under the receiver operating characteristic (ROC) curve) values derived from the analysis of a 1000-residue-long sequence for the Disorder—NOX and Disorder—PDB reference datasets [36]. However, this tool was one of the fastest disorder predictors tested in CAID2, being ranked #5 based on its prediction time of 0.4 s for a sequence of 1000 residues in length. Furthermore, based on its AUC values, PONDR^®^ VSL2 was ranked #2 and #3 (for the Disorder—NOX and Disorder—PDB reference datasets) among the five fastest disorder predictors [36]. These observations indicated that PONDR^®^ VSL2 continues to be a competitive tool characterized by a short execution time and reasonably high accuracy. Therefore, we selected it for our analyses. 

### 2.3. ELMs: Eukaryotic Linear Motifs

The ELM (eukaryotic linear motif) database is a platform used to recognize the SLiMs (short linear motifs) in the proteins [37,38,39,40,41,42,43]. The motifs recognized are special in a way that if the information on the 3D organization of a functional protein is absent, SLiMs still provide a way to evaluate the potential functionality of protein, since these functional motifs are linear, which is a unique property because of the intrinsic disorder nature of these motifs [44]. The identification of these motifs helps in the understanding of the functionality of the protein, as SLiMs are involved in important interactions and perform regulatory roles [42]. In this study, we found the eukaryotic linear motifs in the aggregation hotspots, droplet-promoting regions, multiple binding-mode regions, and molecular recognition feature (MoRF) regions of our selected proteins. The goal was to map the identified ELMs/SLiMs onto these IDRs. By identifying ELMs, the goal was to deepen our understanding of the functionality of our proteins and how they interact and play a role within the cellular environment.

### 2.4. Functional Annotation Derived from Disorder

D^2^P^2^ is a special Database of Disordered Protein Prediction designed to facilitate the statistical comparison among different prediction methods to facilitate the analysis of IDPs [45]. Along with disorder predictions, D^2^P^2^ also shows the localization of MoRF regions, unique disordered binding sites that become ordered following interaction with specific partners, and are found through the ANCHOR algorithm, PTMs, and also list the SUPERFAMILY domains from evolutionary studies [45].

### 2.5. FuzDrop Analysis: Identifying LLPS Promoters

We used FuzDrop [46] to predict the likelihood of proteins taking part in spontaneous liquid–liquid phase separation and generate a scoring system based on the sequence of proteins to identify the regions that promote this process. Protein with a pLLPS (probability of liquid–liquid phase separation) score of 0.60 or higher are identified as promoters of droplet formation and participants of liquid–liquid phase separation, which leads to droplet formation and generates membrane-less organelles that are important for several cellular functions such as stress response and regulation [47].

### 2.6. Protein–Protein Interaction Network

The STRING database strives to incorporate all established and predicted connections among proteins, comprising both the physical and functional associations [48,49,50]. Users get to analyze network visualizations, predicted connections, and functional annotations for the analysis of proteins. PPI networks of proteins were retrieved by using the STRING database (https://string-db.org, accessed on 10 March 2024). For the analysis of protein interactions through STRING, we used a medium confidence level and 500 interactors in the 1st shell to generate the PPI network. For the global interactions network, the 11 most disordered proteins were used to generate a PPI network, using the same settings mentioned above. The functional enrichment data of these proteins can be found in Supplementary Tables S1–S3.

### 2.7. CH-CDF Analysis

CH-CDF graph combined the results of two plots: charge–hydropathy (CH) and cumulative distribution function (CDF). The CH graph is plotted based on the net charge and hydropathy of proteins; disordered proteins tend to have high net charge and low hydropathy, and they are found to be clustered in the specific area of the plot [51,52]. A linear line is placed to separate these disordered proteins from the ordered [51,52]. A CDF plot is based on PONDR scores, plotting PONDR scores to their frequency. PONDR scores tell us about the disorder associated with the protein sequence. For the CH plot, a protein that appears above the linear boundary is considered disordered, and the one that appears below the boundary is considered as ordered [51,52].

For the CDF plot, the CDF curve for ordered proteins is plotted below the order–disorder line when it is considered to be disordered, and if it appears above this boundary, it is labeled as an ordered protein [51].

The CH-CDF plot classified proteins effectively in two categories, ordered and disordered, by plotting the average distance of the protein from the order–disorder boundary (CDF) and the scores obtained through the CH plot [53].

### 2.8. 3D Structures of Proteins

Alpha Fold, a protein structure database developed by DeepMind exploits an AI system to predict the 3D structures of proteins based on the amino acid sequences with a high accuracy [54].

## 3. Results and Discussion

### 3.1. Global Disorder Analysis of Host Proteins Entrapped in RABV Particles

First, to get an overview of the overall disorder status of the host (mouse) proteins entrapped in RABV particles, we analyzed these proteins using a set of commonly used per-residue disorder predictors, such as PONDR^®^ VSL2, PONDR^®^ VL3, PONDR^®^ VLXT, and PONDR^®^ FIT, IUPred Short, and IUPred Long. These predictors were accessed through the Rapid Intrinsic Disorder Analysis Online (RIDAO) platform (available at https://RIDAO.app; accessed on 10 March 2024) [34]. The average disorder scores (ADSs) and percentages of predicted disordered residues (PPDRs) were computed for each protein, employing the outputs of these per-residue predictors. The ADS is a measure of the average disorder for a protein, and the PPDR is a measure of the proportion of amino acids within a protein that have a predicted disorder score above 0.5. 

The results of these analyses are summarized in Supplementary Table S4. These data were used to classify each protein by its disorder status. Of note, since the ADS does not share a direct relationship with the PPDR, we defined proteins as highly ordered if they had a PPDR of less than 10% and/or an ADS of less than 0.15. Proteins with 10% ≤ PPIDR < 30% and/or 0.15 ≤ ADS < 0.5 were considered moderately disordered. Proteins with a PPDR ≥ 30% and an ADS of 0.5 or more were labeled as highly disordered. These categorizations are consistent with the standards set in our previous publications and are in line with the accepted practice in the field [55]. This approach provides the means for a more detailed study of protein structures by clearly identifying varying levels of their structural (dis)organization. 

**Figure 1:**
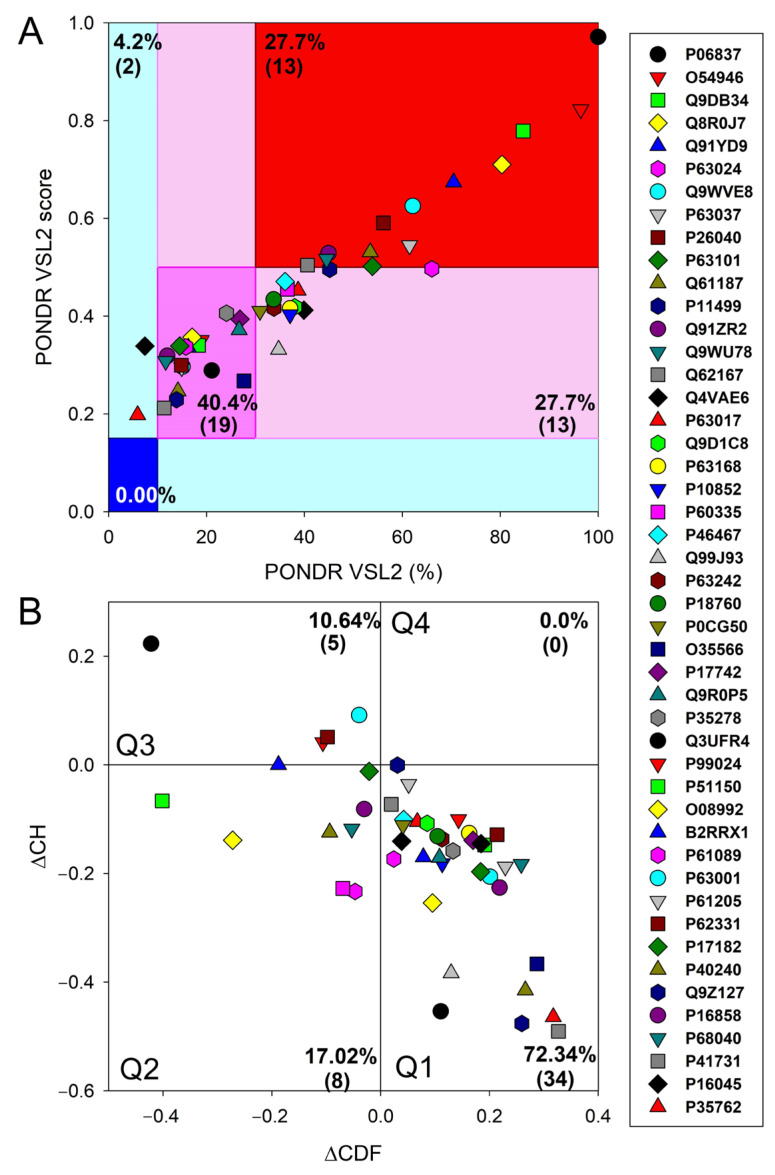
Multifactorial intrinsic disorder analysis of mouse proteins entrapped in RABV particles. (**A**) PONDR^®^ VSL2 score vs. VSL2 PONDR^®^ (%) analysis: PONDR^®^ VSL2 (%) is a percent of predicted disordered residues (PPDR), i.e., residues with disorder scores above 0.5. PONDR^®^ VSL2 score is the average disorder score (ADS) for a protein. Color blocks indicate regions in which proteins are mostly ordered (blue and light blue), moderately disordered (pink and light pink), or mostly disordered (red). If the two parameters agree, the corresponding part of the background is dark (blue or pink), whereas light blue and light pink reflect areas in which the predictors disagree with each other. The boundaries of the colored regions represent arbitrary and accepted cutoffs for ADSs (*y*-axis) and the percentage of predicted disordered residues (PPDRs; *x*-axis). (**B**) Charge–hydropathy and cumulative distribution function (CH-CDF) analysis of entrapped host proteins: The CH-CDF plot is a two-dimensional representation that integrates both the CH plot, which correlates a protein’s net charge and hydrophobicity with its structural order, and the CDF, which cumulates disorder predictions from the N-terminus to the C-terminus of a protein, offering insight into the distribution of disorder residues. The *y*-axis (ΔCH) represents the protein’s distance from the CH boundary, indicating the balance between charge and hydrophobicity, while the *x*-axis (ΔCDF) represents the deviation of a protein’s disorder frequency from the CDF boundary. Proteins are then stratified into four quadrants: Quadrant 1 (bottom right) indicates proteins likely to be structured; Quadrant 2 (bottom left) includes proteins that may be in a molten globule state or lack a unique 3D structure; Quadrant 3 (top left) consists of proteins predicted to be highly disordered; and Quadrant 4 (top right) captures proteins that present a mixed prediction of being disordered according to CH but ordered according to CDF.

Since the effectiveness and accuracy of PONDR^®^ VSL2 has been proven in the Critical Assessment of protein Intrinsic Disorder [35], we used the outputs of this tool to generate an illustrative representation of global disorder distribution in mouse proteins entrapped in the RABV particles. The results of this analysis are shown in Figure 1A, which indicates that most of the host proteins are predicted as moderately or highly disordered. 

In fact, approximately 27.7% of entrapped host proteins are in the red zone (highly disordered), and an additional 27.7% are in the light pink zone (i.e., proteins with PPDR_VSL2_ ≥ 30% but 0.15 ≤ ADS_VSL2_ < 0.5). Furthermore, 40.4% of proteins are predicted as moderately disordered; they are located within the dark pink area and are therefore characterized by 10% ≤ PPIDR_VSL2_ < 30% or 0.15 ≤ ADS_VSL2_ < 0.5. None of these proteins was predicted as highly ordered based on their PPIDR_VSL2_ and ADS_VSL2_ data, and only two were placed in the light cyan area, being characterized by PPDR_VSL2_ < 10% but ADS_VSL2_ > 0.15. Figure 1A also shows that neuromodulin (UniProt ID: P06837) represents a noticeable exception, being located at the top corner of the red zone and being notably separated from other data points. These observations suggest that neuromodulin has a much higher disorder propensity than the rest of the dataset. The detailed characterization of neuromodulin as a highly disordered protein could be of particular interest for further investigation in relation to its unique functional implications in a wide range of biological processes, as well as its disease associations. 

To gain further insight into the structural organization of the entrapped host proteins, we combined the outputs of two binary disorder predictors to their outputs using the charge–hydropathy (CH) plot, which classified proteins based on the distribution of charged amino acids, and the cumulative distribution function analysis. Compared to ordered proteins, disordered proteins often have a lower hydrophobicity and higher net charge [51,52]. The CDF describes the cumulative frequency of disordered proteins along the length of a given protein. If the CDF curve of a given protein is below the order–disorder boundary, this protein is considered to be disordered and is considered ordered if the CDF curve is located above this boundary [51]. The outputs of these binary predictors were used to generate the ∆CH-∆CDF plot, presenting us with the global disorder analysis for our sets of proteins [53,56]. With this technique, we were able to classify proteins based on where they fell on the plot. Quadrant 1 (Q1, bottom right) encompasses proteins that are likely structured. Quadrant 2 (Q2, bottom left) comprises proteins that are either molten globular or hybrid, i.e., proteins that are compact yet lack a distinctive 3D structure or contain noticeable levels of ordered and disordered residues. Quadrant 3 (Q3, top left) includes highly disordered proteins, whereas Quadrant 4 (Q4, top right) captures proteins that are predicted to be disordered according to the CH plot yet ordered according to the CDF plot [53,56]. Therefore, based on their position within the ∆CH-∆CDF phase space, proteins can be classified into ordered with a stable structure, molten and globule-like (not completely ordered and disordered, with a flexible structure), and highly disordered proteins lacking a stable 3D structure. 

Figure 1B represents the results of the global disorder analysis of the entrapped host proteins in the form of the ∆CH-∆CDF graph. The top left quadrant is designated as Quadrant 3; it is where both binary predictors agree that the protein is unstructured and called the disorder quadrant. Neuromodulin is again acting as an outlier in the ∆CH-∆CDF plot, occupying the top-most position in Q3. In addition to neuromodulin, this quadrant contains four more highly disordered proteins. Furthermore, eight entrapped mouse proteins are classified as molten and globular or hybrid, whereas all the remaining proteins in this dataset (34 or 72.34%) are placed in Q1, indicating that they are expected to be mostly ordered. There are no proteins in the upper right quadrant (Q4). Some proteins are located at the boundaries between two quadrants, suggesting they may have mixed characteristics attributed to both adjacent quadrants, indicating that these proteins may have flexible structures. 

**Figure 2:**
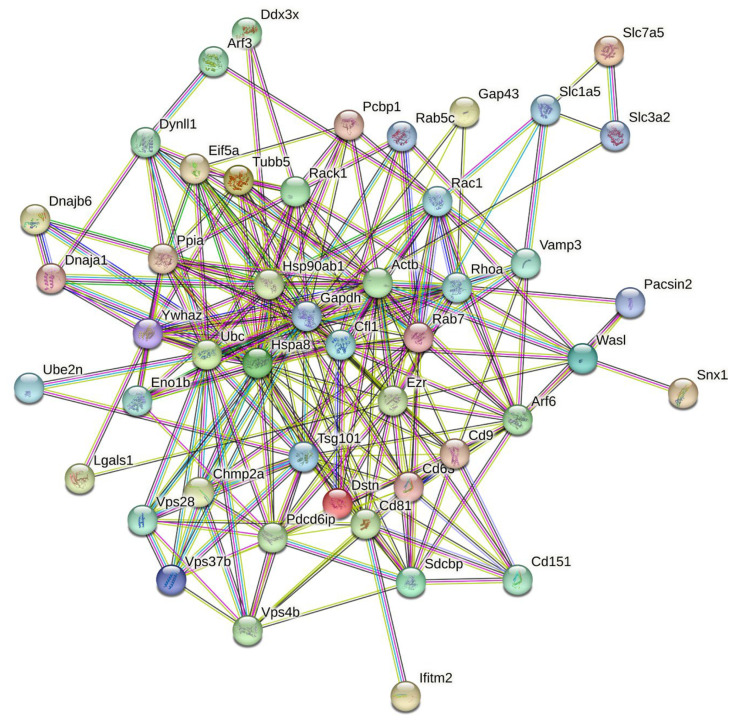
STRING-based analysis of the intra-set interactivity of 47 mouse proteins entrapped in RABV particles. In the corresponding network, the nodes correspond to proteins, whereas the edges show predicted or known functional associations. Seven forms of evidence are used to build the corresponding network and are indicated by the differently colored lines: a green line represents neighborhood evidence; a red line, the presence of fusion evidence; a purple line, experimental evidence; a blue line, co-occurrence evidence; a light blue line, database evidence; a yellow line, text mining evidence; and a black line, co-expression evidence [50].

Next, we analyzed the intra-set interactivity of mouse proteins entrapped in RABV particles. To this end, we utilized the STRING platform, which generates a protein–protein interaction (PPI) network of predicted associations based on predicted and experimentally validated information on the interaction partners of a protein of interest [50]. Surprisingly, Figure 2 shows that all 47 proteins analyzed in this study were involved in the formation of a rather dense PPI network, which is characterized by an average node degree of 10.3 and an average local clustering coefficient of 0.651. Proteins in this network are involved in 243 PPIs, which significantly exceeds the expected number of interactions (69) for a random set of proteins of the same size and degree distribution drawn from the genome. Table 1 represents the most enriched biological processes, molecular functions, and cellular components (as per Gene Ontology annotations) of the members of this network. 

Note that Table 1 represents GO terms corresponding to the five biological processes, molecular functions, and cellular components characterized by the lowest false discovery rates (a measure that describes the enrichment significance evaluated as *p*-values corrected for multiple testing within each category using the Benjamini–Hochberg procedure). However, the complete lists of the GO terms found in this STRING-based analysis include 324 biological processes, 35 molecular functions, and 106 cellular components. In agreement with Zhang et al. [23], who indicated that based on the associated biological processes, virion-packed mouse proteins can be grouped into 12 functional categories, such as cell adhesion, cytoskeleton organization, endocytosis, exosomal secretion, morphogenesis, protein localization, protein ubiquitination, regulation of gene expression, transcription, translation, transport, and viral processes; our analysis also found all these functional categories. Some of the viral life cycle-related biological processes ascribed to the virion-entrapped mouse proteins included viral budding (Vsp4b, Tsg101, Chmp2a, and Pdcd6ip), viral budding from the plasma membrane (Vsp4b and Pdcd6ip), viral budding via the host ESCRT complex (Vsp4b, Chmp2a, and Pdcd6ip), the viral life cycle (Cd81, Chmp2a, Hsp90ab1, Pcbp1, Pdcd6ip, Rab7, Slc3a2, Tsg101, Vps37b, and Vps4b), viral release from the host cell (Vsp4b, Chmp2a, Vps37b, Tsg101, and Rab7), the regulation of the viral life cycle (Ddx3x, Ifitm2, Lgals1, Ppia, Tsg101, and Vps37b), the regulation of viral genome replication (Ddx3x, Ppia, and Ifitm2,), the regulation of viral process (Ddx3x, Ifitm2, Lgals1, Ppia, Rab7, Tsg101, and Vps37b), and positive regulation by the host of the viral process (Cfl1 and Hspa8). For the complete lists of biological processes, molecular functions, and cellular components ascribed by STRING to 47 mouse proteins entrapped in RABV particles, see Supplementary Tables S5–S7. 

**Figure 3:**
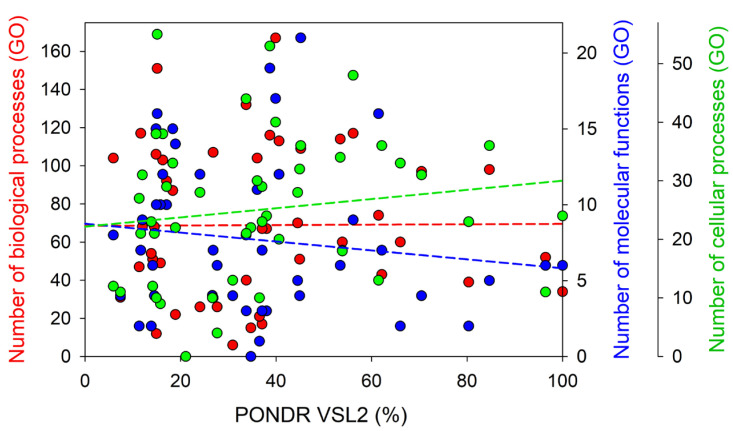
Dependence of the number of biological processes (red circles), molecular functions (blue circles), and cellular components (green circles) ascribed by STRING to 47 mouse proteins entrapped in RABV particles on their level of intrinsic disorder, evaluated as the PPIDR_VSL2_.

Importantly, based on the results of this analysis, almost none of the proteins were found to be unifunctional, and instead, most of the proteins had numerous functions and were classified in multiple functional categories. This observation is illustrated in Figure 3, showing the dependence of the number of biological processes, molecular functions, and cellular components ascribed by STRING to 47 mouse proteins entrapped in RABV particles on their levels of intrinsic disorder. Figure 3 shows that the number of biological processes ascribed to each mouse protein analyzed in this study was not correlated with their level of protein disorder. On the other hand, the number of molecular functions and cellular components showed some negative and positive correlations with the protein disorder level. 

This STRING-based analysis revealed that the number of biological processes attributed to a single protein ranged from 167 to 6 for the transforming protein RhoA and polyubiquitin-C (Ubc), respectively. Fourteen proteins were shown to be involved in more than one hundred biological processes each: Rhoa (167), Rac1 (151), Cfl1 (132), Ezr (117), Rack1 (117), Hspa8, (116), Tsg101 (114), Ddx3x (113), Hsp90ab1 (109), Ppia (107), Arf6 (106), Vps4b (104), Cd81 (104), and Actb (103). Eight of these proteins were predicted as highly disordered (Ezr (56.14%), Tsg101 (53.45%), Hsp90ab1 (45.17%), Ddx3x (40.63%), Rhoa (39.90%), Hspa8 (38.70%), Vps4b (36.04%), and Cfl1 (33.73%)), as their PPIDRs ≥ 30%. Five proteins (Ppia (26.83%), Actb (16.27%), Rac1 (15.10%), Arf6 (14.86%), and Rack1 (11.67%)) were characterized by the 10% ≤ PPIDR < 30% and were therefore classified as moderately disordered. Finally, Cd81 had a PPIDR of 5.93% and was identified as mostly ordered. 

The number of STRING-identified molecular functions of the 47 mouse proteins entrapped in RABV particles ranged from 21 (Hsp90ab1) to 1 (Pcbp1). There were 15 proteins, each associated with at least 10 functions: Hsp90ab1 (21), Hspa8 (19), Rhoa (17), Rac1 (16), Dnaja1 (16), Arf6 (15), Rab7 (15), Tubb5 (14), Ddx3x 12), Actb (12), Rab5c (12), Vps4b (11), Sdcbp (10), Ube2n (10), and Arf3 (10). Six of these proteins were predicted as highly disordered: Dnaja1 (61.46%), Hsp90ab1 (45.17%), Ddx3x (30.63%), Rhoa (39.90%), Hspa8 (38.7%), and Vps4b (36.04%), whereas nine proteins were classified as moderately disordered: Rab5c (24.07%), Tubb5 (18.92%), Rab7 (18.36%), Sdcbp (17.06%), Actb (16.27%), Ube2n (15.79%), Rac1 (15.10%), Arf3 (14.92%), and Arf6 (14.86%).

Based on the outputs of our STRING analysis, the largest number of cellular components (55) was ascribed to Rac1, whereas Cd151 was shown to be characterized by the least number of cellular components, with 4. There were 17 proteins associated with at least 30 cellular components each: Rac1 (55), Hspa8 (53), Ezr (48), Cfl1 (44), Rhoa (40), Arf6 (38), Actb (38), Hsp90ab1 (36), Pacsin2 (36), Chmp2a (36), Tsg101 (34), Rab7 (33), Vamp3 (44), Snx18 (32), Gapdh (31), Wasl (31), and Vps4b (30). Twelve of these proteins were identified as highly disordered: Chmp2a (84.68%), Wasl (70.46%), Vamp3 (60.02%), Pacsin2 (62.14%), Ezr (56.14%), Tsg101 (53.45%), Hsp90ab1 (45.17%), Snx18 (44.95%), Rhoa (39.90%), Hspa8 (38.70%), Vps4b (36.04%), and Cfl1 (33.73%), with the remaining five proteins being moderately disordered: Rab7 (18.36%), Actb (16.27%), Rac1 (15.10%), Arf6 (14.86%), and Gapdh (12.01%).

It was indicated earlier that many host proteins found in RABV particles were also identified in other viruses from 11 viral families [23]. That study also emphasized that 15 host proteins were most frequently recruited by different viruses: Actb (IAV, HIV, VSV, RVFV, IBV, HSV, and KSHV), Cd9 (IAV, HAV, MEV, HIV, and ASFV), Cd81 (IAV, HCV, MEV, HIV, and VV), Cfl1 (IAV, RSV, HIV, and HSV), Eno1b (IAV, HAV, MEV, HIV, VSV, IBV, ASFV, and KSHV), Gapdh (IAV, RSV, HIV, RVFV, IBV, ASFV, and KSHV), Hspa8 (RSV, HIV, VSV, RVFV, HSV, ASFV, and JUNV), Hsp90ab1 (RSV, HAV, HIV, VSV, RVFV, IBV, KSHV, and SARS), Pdcd6ip (HAV, HIV, VSV, HSV, and JUNV), Ppia (IAV, MEV, HIV, HSV, and KSHV), Rab5c (HAV, HIV, HIV, ASFV, and HSV), Rab7a (HAV, HIV, RVFV, HSV, KSHV, and JUNV), Tubb5 (IAV, RSV, HIV, VSV, and ASFV), Ubc (IAV, RSV, HIV, VSV, and JUNV), and Ywhaz (HIV, HSV, KSHV, and SARS) [23]. These observations indicated that such “multiviral” host proteins (i.e., those widely recruited by different viruses) might endow viruses with some benefits for their replication cycles [23]. We looked at the intrinsic disorder predispositions of these proteins and found that six of them (Ywhaz (53.88%), Hsp90ab1 (45.17%), Pdcd6ip (44.53%), Hspa8 (38.70%), Cfl1 (33.73%), and Ubc (30.93%)) are highly disordered, eight (Ppia (26.83%), Rab5c (24.17%), Tubb5 (18.92%), Rab7a (18.36%), Actb (16.27%), Eno1b (14.52%), Cd9 (14.15%), and Gapdh (12.01%)) are moderately disordered, whereas Cd81 (5.93%) is mostly ordered. Therefore, the results of this analysis indicate that intrinsic disorder might also contribute to the “multiviral” functionality of these proteins. 

**Figure 4:**
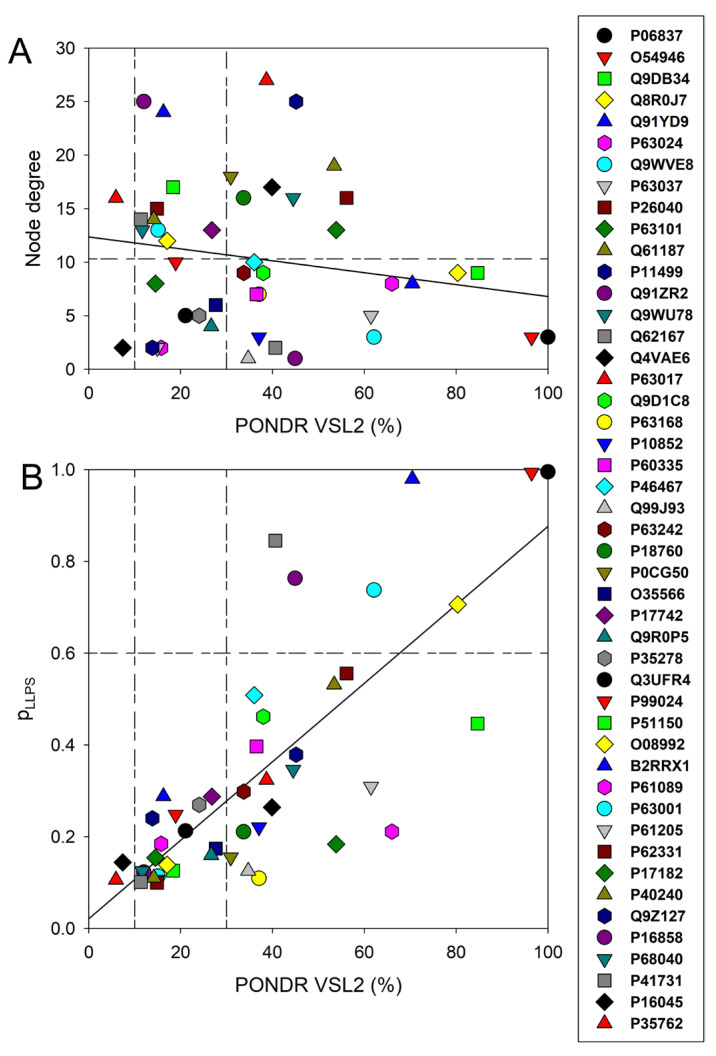
Correlation between the intrinsic disorder levels in the host proteins entrapped in RABV particles and their interactivity within the intra-set PPI (**A**) and predisposition for being involved in liquid–liquid phase separation, LLPS (**B**). Solid lines in both plots show linear fits of the reported data, whereas short–long–dashed lines represent boundaries between different disorder categories, as well as between hubs and non-hubs (**A**) and LLPS promoters and other proteins (**B**).

Next, we looked for the presence of a correlation between the level of intrinsic disorder in a given protein and its interactivity within the intra-set PPI network (i.e., its node degree). The results of this analysis are shown in Figure 4A, illustrating that such a correlation is almost absent. 

Figure 4A shows that in the intra-set PPI network analyzed in this study, almost half of the mouse proteins entrapped in the RABV particles are engaged in more than 12 interactions (i.e., serve as hubs of this network, with a hub being defined here as a node, with the number of interactions exceeding the average node degree of this network, which is 10.3). However, there is no clear disorder enrichment among hubs. These observations suggest that this intra-set PPI network is almost disorder neutral. This is a rather interesting and unexpected observation, as typically, there is a strong positive correlation between the protein interactivity and its intrinsic disorder predisposition. In fact, it is indicated in many studies that one of the remarkable functional features of IDPs and IDRs is their extraordinary binding promiscuity [33,57,58,59,60,61]. Therefore, IDPs/IDRs are considered as binding “professionals”, which continuously interact with various partners via multiple binding modes [33,57,58,59] and form static, semi-static, dynamic, or fuzzy complexes [60,61]; as well, they can be engaged in polyvalent interactions, where multiple binding sites of one protein are simultaneously bound to multiple receptors on another protein [62]. Often, disorder-based interactions are characterized by a combination of high specificity and low affinity [63], and many IDPs/IDRs can fold (at least partially) as a result of binding to their partners [64,65,66]. The degree of such binding-induced folding can be different in various systems, thereby forming complexes with broad structural and functional heterogeneity [60,61]. Furthermore, some IDPs/IDRs are capable of adopting different structures while forming complexes with different partners, thereby acting as morphing shape changers [58,66,67,68,69,70,71,72,73,74,75]. Often, significant levels of disorder are retained by IDPs/IDRs in their bound state (at least outside the binding interface), resulting in the formation of so-called fuzzy complexes [76,77,78,79,80,81,82,83]. Therefore, it is not surprising that many IDPs/IDRs serve as hub proteins: nodes in complex PPI networks that have a very large number of connections to other nodes [71,84,85,86,87,88,89]. As is shown in Figure 1A and Figure 4A, only two mouse proteins entrapped in the RABV particles are classified as mostly ordered (Galectin-1, UniProt ID: P16045 and CD81 antigen, UniProt ID: P35762), whereas all other proteins contain noticeable levels of disorder. It is therefore very likely that the IDRs found in all these moderately and highly disordered proteins are related to their interactability. Furthermore, considered here that the PPI network characterizes only the intra-set connectivity, it does not describe the overall interactivity of these proteins. In fact, as it follows from our comprehensive analyses of the most disordered proteins (see below), all of them are expected to be highly promiscuous binders. For example, STRING-generated PPI networks centered at the mouse neuromodulin (UniProt ID: P06837; PPIDR_VSL2_ = 100.0%), Chmp4b (UniProt ID: Q9D8B3; PPIDR_VSL2_ = 84.7%), DnaJB6 (UniProt ID: O54946; PPIDR_VSL2_ = 96.4%), Vps37B (UniProt ID: Q8R0J7; PPIDR_VSL2_ = 80.4%), and Wasl (UniProt ID: Q91YD9; PPIDR_VSL2_ = 70.5%) contain 145, 100, 68, 42, and 232 nodes, respectively (see below). This is in striking contrast to their intra-set node degrees of 3, 9, 3, 9, and 8, respectively (see Figure 4A). Finally, one should keep in mind that although a positive correlation between the protein interactivity and intrinsic disorder predisposition is typically observed, ordered proteins can serve as hubs as well, but in this case, partners of such ordered hubs are mostly IDPs or proteins with IDRs [71,75]. 

Next, we analyzed the predisposition of mouse proteins entrapped in the RABV particles to serve as drivers of liquid–liquid phase separation (LLPS) using the FuzDrop platform [46]. The results of this analysis are summarized in Figure 4B, showing dependence of the probability of analyzed proteins for spontaneous liquid–liquid phase separation, p_LLPS_, on their intrinsic disorder status. This analysis revealed that there is a strong positive correlation between PPIDR_VSL2_ and p_LLPS_, and all seven proteins predicted as droplet drivers (i.e., proteins characterized by p_LLPS_ ≥ 0.60) are also predicted to be highly disordered. It is recognized now that a significant part of cellular processes is determined by the functioning of liquid droplet-like condensates: membrane-less organelles (MLOs) [90,91]. In fact, MLOs are very diverse and commonly found in the cytoplasm, nucleus, mitochondria of various eukaryotic cells, chloroplasts of plant cells, as well as in bacterial cells. Biogenesis of MLOs is driven by the intracellular LLPS processes, which are also known as liquid–liquid demixing phase separation [92,93] and are strongly dependent on IDPs and IDRs [94,95]. In fact, many of the MLO resident proteins are IDPs or contain IDRs, and the formation of all the MLOs analyzed so far relies on IDPs/IDRs, indicating that intrinsic disorder is important for MLO biogenesis [92]. 

After subjecting all 47 mouse proteins found in the rabies virus to the intrinsic disorder analysis, we selected the 11 most disordered proteins for a comprehensive analysis, with 5 of these highly disordered proteins being discussed in detail (see below for discussion of neuromodulin and Appendix A for the detailed discussions of Chmp4b, DnaJB6, Vps37B, and Wasl). The information about the remaining highly disordered proteins (Pascin2, Ddx3x, Snx18, Tsg101, and Ezr) can be found in the Supplementary Materials (see Supplementary Figures S1–S5).

### 3.2. Functional Intrinsic Disorder in the Most Disordered Mouse Proteins Found in the Rabies Virus

#### Neuromodulin (UniProt ID: P06837)

Neuromodulin is a protein encoded by the gene *Gap43*. This protein is involved in neuron growth acting as a crucial component of the growth cones present at the tips of elongating axons (https://www.uniprot.org/uniprotkb/P06837/entry; accessed on 10 March 2024). 

In mice, neuromodulin is a peripheral membrane protein that is not entirely embedded in the membrane but associated with it, which allows for its dynamic interaction with other membrane proteins. Neuromodulin is transported to the growth cones of neurons. These growth cones are present at the tips of the axons and are essential for guiding the direction of neuronal growth during development and regeneration. Several studies have been conducted to elucidate the process by which protein is transported to the growth cones. Zuber et al. suggested that the N-terminal, ten-amino acid sequence is sufficient to target the protein to these growth cones [96]. However, later, an experiment conducted with a fusion protein combining neuromodulin and β-galactosidase, which is an enzyme used as a marker in an experiment, revealed that the N-terminal, ten-amino acid sequence only is not sufficient to transport a protein to its target, and the protein’s ability to attach to the membrane through palmitoylation at cysteines 3 and 4 is also essential for assembling the protein at the growth cones [97,98]. This also signifies the importance of post-translational modification in the protein. 

The mouse neuromodulin is a 227-residue long, highly disordered protein of 23.6 kDa, whose interactions with calmodulin along with neurogranin are crucial for learning and memory formation in the nervous system [99]. This protein, which is also designated as GAP-43 or P-57 neuromodulin, is one of the main presynaptic substrates of protein Kinase C [99,100,101]. The phosphorylation of neuromodulin leads to a decreased affinity for calmodulin [99]. Under low-calcium-ion conditions, the protein binds to calmodulin through a highly unstructured IQ motif (I/L/V) QXXXRXXXX(R/K), which adopts an α-helical confirmation upon binding with calmodulin [99]. Phosphorylation through protein Kinase at serine residues modulates this interaction, influencing the behavior of F actin in the growth cones of neurons [100].

Along with this, this protein consists of a “Gap junction protein N-terminal region” (residues 2–31) and IQ motif (residues 31–60). Phosphorylation occurs at Ser41, Ser86, Serine96, Thr88, Thr89, Thr89, Thr95, Ser96, Ser103, Thr138, Ser142, Ser144, Ser145, Thr172, Ser192, and Ser 193. Palmitoylation at cysteine residues at positions 3 and 4 (more specifically, S-palmitoyl cysteine modification) is important for protein association with the cellular membrane and its location. The loss of these modifications at these sites are mutations associated with PTM and can prevent the protein from properly being lipidated and lead to changes in the protein function and location (https://www.uniprot.org/uniprotkb/P06837/entry; accessed on 10 March 2024). 

**Figure 5:**
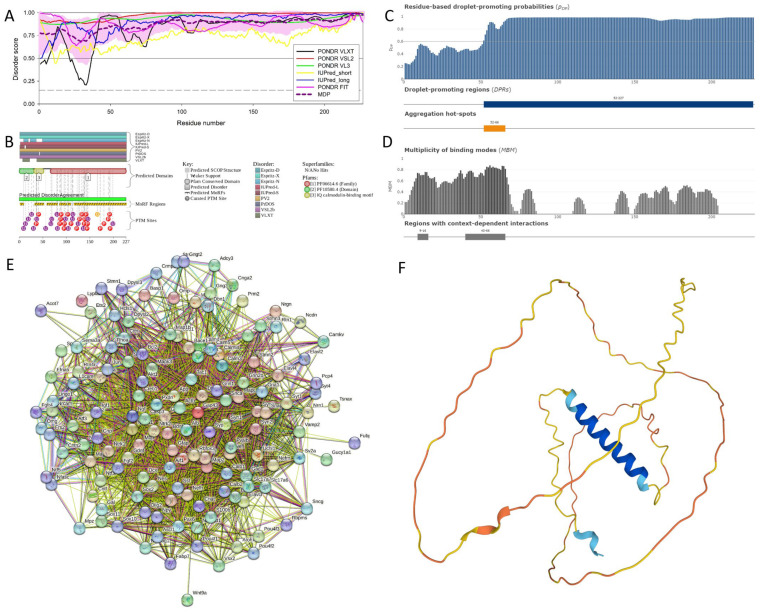
Functional disorder analysis of mouse neuromodulin (UniProt ID: P06837). (**A**) Per-residue disorder profile generated using RIDAO, showing that a major portion of this protein has a predicted value of disorder above the established threshold (0.5). (**B**) Functional disorder profile generated for neuromodulin using the D^2^P^2^ database, showing the outputs of several disorder predictors such as VLXT, VSL2b, PrDOS, IUPred, and Espritz. The colored bar highlighted by blue and green shades represents the disorder prediction; colored circles below the bar shows the predicting PTMs. (**C**) The FuzDrop-generated plot showing the sequence distribution of the residue-based, droplet-promoting probabilities, p_DP_. (**D**) The FuzDrop-generated plot of the multiplicity of binding modes, showing positions of regions that can sample multiple binding modes in the cellular context (sub-cellular localization, partners, and posttranslational modifications)-dependent manner (residues 9–16 and 40–66). (**E**) Protein–protein interaction network generated using STRING. This PPI network was generated by using the minimum required interaction score of 0.4 (medium confidence) and adjusting the value of a maximum number of interactors to 500. Network nodes represent individual proteins, and edges represent protein–protein interactions for shared function, with the types of interactions; the blue line represents curated databases, the black line is for co-expression, and the green line is for the gene neighborhood. (**F**) The 3D structural model is predicted through AlphaFold. The structure is colored according to the per-residue model confidence score, ranging from orange to blue fragments of the structure, from a very low (p_LDDT_ < 50) value to very high confidence (p_LDDT_ > 90), respectively.

Figure 5 represents the results of the functional disorder analysis of this protein. The per-residue disorder profile generated using RIDAO indicates that neuromodulin is predicted as a highly disordered protein (see Figure 5A). In fact, the PPIDR scores determined using the disorder predictors PONDR^®^ FIT, PONDR^®^ VSL2, PONDR^®^ VL3, PONDR^®^ VLXT, IUPred Short, and IUPred Long were 100%, 100%, 100%, 90.75%, 96.68%, and 99.56%, respectively. The mean disorder profile (MDP) was 100%, signifying that the protein is highly disordered. The residues are predicted to be disordered above the 0.5 threshold, and an MDP value of 100% implies that neuromodulin in its entirety is likely to be intrinsically disordered [34]. 

The D^2^P^2^ platform was used to generate a functional disorder profile for neuromodulin (see Figure 5B). The top section of the image is showing colored bars that represent the disordered regions predicted by each predictor, such as IUPred-L, IUPred-S, PV2, PrDOS, VSL2b, VLXT, Espritz-D, Espritz-X, and Espritz-N [45]. Below these colored bars of predicted disorder is the domain prediction bar exhibiting three domains, with one of these domains marked as number 3, being the IQ domain of neuromodulin we discussed above. It ranges from residue 31 to 50 and is known as an IQ calmodulin-binding motif. 

The consensus bar of a green color is the predicted disorder agreement between all predictors. According to D^2^P^2^ platform, all the predictors agree that the disorder regions are found at residues 2–227. This protein is highly unstructured, being the most disordered among all the 47 host proteins analyzed in this study. Moving on with the D^2^P^2^ results, the yellow zigzagged lines represent MoRF regions. MoRF regions is short for molecular recognition features, which are disordered protein regions that become ordered upon binding to the respective protein partners [102]. Multiple MoRF regions are found at the ranges 1–9, 32–52, 58–81, 102–109, and 116–227, identified through the ANCHOR algorithm and also named as the disorder-based binding sites, indicating that neuromodulin has a tendency to engage in disorder-to-order transition-based interactions. Below these MoRF region predictions are the differently colored circles with letters representing the PTMs sites along the length of the protein. Other than this, D^2^P^2^ also included the superfamily annotation and Pfam domains, indicating the large family the protein belongs to and the shared structural and functional domains within the family, giving insight into the role of the protein and its functional profile.

Figure 5C represents the FuzDrop-generated plot, showing the sequence distribution of the residue-based, droplet-promoting probabilities, p_DP_. Residues with p_DP_ values above 0.6 are capable of promoting liquid–liquid phase separation. In neuromodulin, most of the residues have p_DP_ values above the indicated threshold. Therefore, most of the neuromodulin residues have a high probability of promoting droplet formation. Peaks in the graph indicate the regions that can promote the formation of membrane-less organelles in the cells through liquid–liquid phase separation. Membrane-less organelles are liquid compartments within the cell involved in specific biological functions, like in gene regulation, that are not enclosed by traditional lipid membranes [103]. In neuromodulin, the droplet-promoting region, i.e., a region that is particularly susceptible to phase separation, is located at the residues 2–127. Furthermore, neuromodulin contains one aggregation hotspot (residues 52–66), which is a region with high probability of promoting droplet formation that is predicted to exhibit a multiplicity of binding modes, enabling the adaptability of interactions to the cellular context. Furthermore, the p_LLPS_ value was predicted to be 0.9949 for neuromodulin. Since the proteins with p_LLPS_ ≥ 0.60 are designated as droplet drivers, with a tendency to undergo spontaneous liquid–liquid phase separation, mouse neuromodulin is predicted as a protein with a very high droplet-driving potential. 

Figure 5D shows a FuzDrop-generated multiplicity of binding modes (MBM) plot, indicating that the protein can bind to multiple partners, behaving differently in terms of its structure and function, either as an ordered or disordered state depending on the type of interaction and its environment. Values of MBM ≥ 0.65 suggest that the residues/regions are context dependent and are prone to engage in multiple interactions. The bar graph shows positions of MBM regions (residues 9–16 and 40–66) that have the potential to be engaged in multiple binding modes, assisting the phase separation. 

The interactability of neuromodulin was evaluated using the STRING database. Figure 5E reveals that this protein is acting as the central node in the complex PPI network. We used a medium confidence threshold and a maximum limit of 500 interactors to generate this PPI network, which contains 145 nodes, with each node representing a protein, including neuromodulin, and 2925 edges (protein–protein interactions). This number of edges in the neuromodulin-centered network significantly exceeds the number of edges expected for a random set of proteins of the same size and degree distribution drawn from the genome (which is 616). The average node degree of this network is observed to be 39, indicating that the average connectivity of each protein in the network is very high, which is further supported by the average local clustering coefficient of 0.659, indicating a high tendency of nodes to cluster together. Finally, the observed *p*-value of <1.0 × 10^−16^ is indicative of the high significance of the generated data, suggesting that the PPI network is unlikely to be produced by chance. Table 2 lists the most enriched biological processes, molecular functions, and cellular components of the members of the neuromodulin-centered PPI network. 

Figure 5F illustrates the 3D structure of the protein predicted by AlphaFold. Since disordered proteins or protein regions do not have single structures but represent highly dynamic conformational ensembles, they cannot be predicted by AlphaFold and are characterized by very low p_LDDT_ scores. In fact, based on the results of the CAID2 experiment, it has been concluded that AlphaFold2-based disorder predictors are better at detecting absence of order rather than detecting disordered regions [36]. Most of the predicted structure of our protein has low confidence scores and would be in disordered form when not interacting with the partners. In short, most of the protein would be unstructured in isolation, as the average per-residue model confidence score p_LDDT_ is 55.78 for this protein. The only high-confidence structural element of this protein is the blue α-helical region (residues 27–52). However, single α-helix cannot exist in isolation and is likely to be induced by binding to specific partner(s). In line with these considerations, this helical region corresponds to the IQ motif responsible for calmodulin binding. 

Finally, we looked at the localization of ELMs (short functional motifs) within the various regions found in neuromodulin. The results of this analysis are summarized in Table 3. 

The data reported in this section indicate that neuromodulin is characterized by a high level of intrinsic disorder with strong functional potential. 

### 3.3. Global PPI Networks Analysis of the Most Disordered Mouse Proteins Found in the Rabies Virus

**Figure 6:**
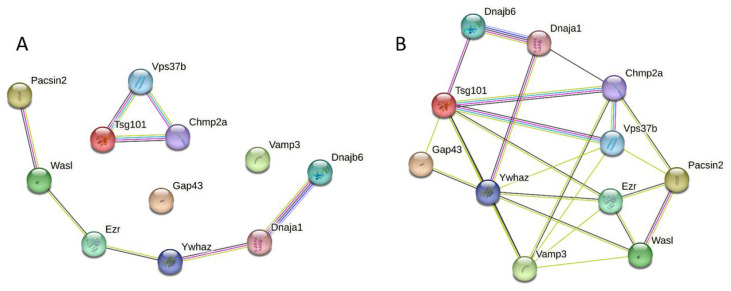
Intra-set interactivity of the 11 most disordered mouse proteins entrapped in RABV particles. Networks are constructed using STRING, using medium confidence of 0.4 (**A**) and low confidence of 0.15 (**B**).

Next, we looked at the interconnectivity of the members of a group of the 11 most disordered mouse proteins found in RABV particles. The results of this analysis are shown in Figure 6. When this set was analyzed using STRING, using a medium confidence of 0.4 for the minimum required interaction score, these proteins were not linked in a single network but formed two disconnected networks consisting of six and three proteins, with two proteins, vesicle-associated membrane protein 3 (Vamp3) and neuromodulin (Gap43), being the loners (see Figure 6A). Although 11 proteins were connected through 8 interactions within this disjoined network (defining the low node degree of 1.45), they still had more interactions among themselves than what would be expected for a random set of proteins of the same size and degree distribution drawn from the genome (1). When the confidence of the minimum required interaction score was decreased to 0.15 (low confidence), all 11 proteins became engaged in interactions and formed a single PPI network with 25 edges and an average node degree of 4.55 (see Figure 6B). Table 4 lists the most enriched biological processes, molecular functions, and cellular components of the members of this PPI network.

**Figure 7:**
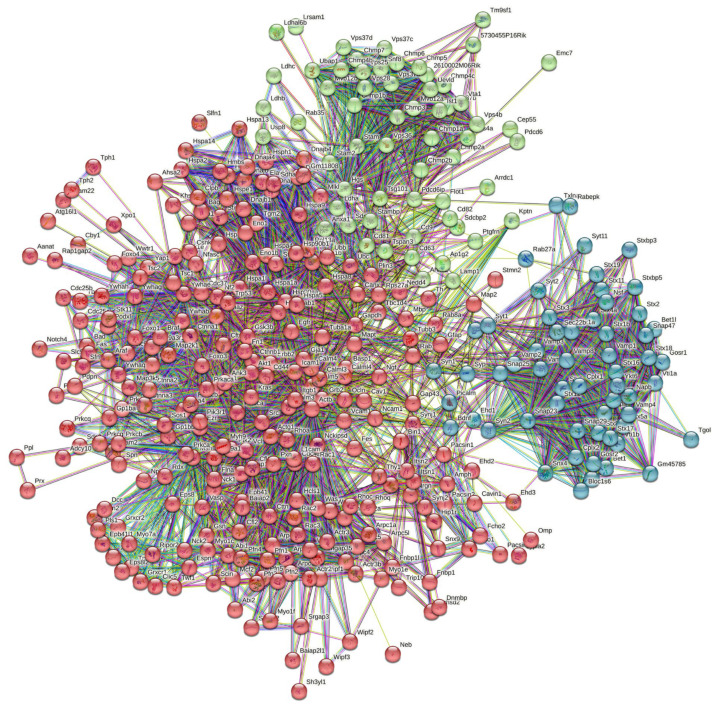
Global interactivity of the 11 most disordered mouse proteins found in the RABV particles. Using k-means clustering (the algorithm, which is included in STRING, automatically assigns data points to one of the K clusters depending on their distance from the center of the clusters), this PPI network can be divided into three clusters.

We also checked the set-centered interactivity of these 11 most disordered mouse proteins found in RABV particles. To this end, we used the multiple proteins search option on the STRING platform and selected a custom value of 500 maximum first-shell interactions (note that the number of interactors in STRING is limited to 500) and high confidence level (minimum required interaction score of 0.7). Using these settings resulted in the generation of a well-connected PPI network containing 281 proteins involved in 3918 interactions (see Figure 7). The average node degree of this network is 20.6, and its average local clustering coefficient is 0.618. 

The application of k-means clustering (which is an unsupervised machine learning algorithm designed to group the unlabeled datasets into different clusters, thereby dividing a set of data into a number of groups depending on how similar and different they are to one another) to this PPI network centered at the 11 most disordered mouse proteins found in RABV particles revealed that the set of 381 proteins can be split into 3 clusters. 

The biggest cluster includes 263 proteins involved in 2312 interactions (see red circles in Figure 7). This sub-network includes many proteins from the regulation of the actin cytoskeleton pathway (KEGG pathway ID: mmu04810, *p*-value = 1.98 × 10^−39^). The average node degree of this network is 17.6, and its average local clustering coefficient is 0.587. 

The second cluster includes 60 proteins involved in 481 interactions (see green circles in Figure 7) and is mostly related to the endocytosis pathway (KEGG pathway ID: mmu04144; *p*-value = 1.50 × 10^−44^). This sub-network is characterized by an average node degree of 16 and average local clustering coefficient of 0.813. 

In the third cluster, there are 58 proteins connected by 875 interactions (see blue circles in Figure 7). Most of the proteins in this cluster are related to the SNARE interactions in the vesicular transport pathway (KEGG pathway ID: mmu04130l *p*-value = 6.94 × 10^−62^). This sub-network is characterized by an average node degree of 30.2, and the average local clustering coefficient of 0.868.

Table 5 represents the most enriched biological processes, biological functions, and cellular components of the members of this PPI network and of each of its clusters. 

**Figure 8:**
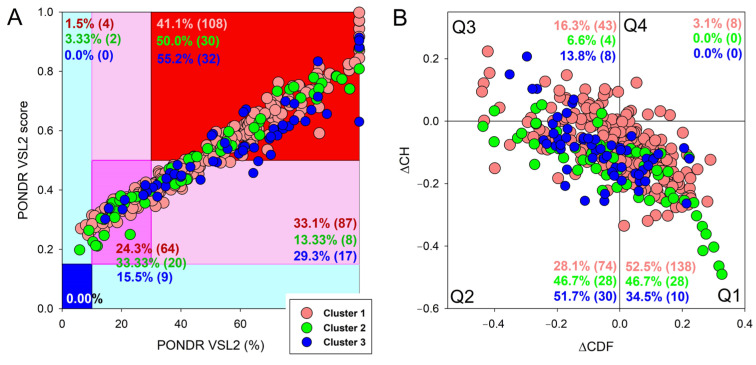
Multifactorial intrinsic disorder analysis of the host interactors of the 11 most disordered mouse proteins found in the RABV particles. (**A**) PONDR^®^ VSL2 score vs. VSL2 PONDR^®^ (%) analysis. (**B**) CH-CDF analysis of the host interactors of entrapped mouse proteins. Data for three clusters discussed in the manuscript are shown by pink, green, and blue symbols, respectively.

To get a hint on the prevalence of intrinsic disorder in host interactors of mouse proteins entrapped in the RABV particle, we applied the RIDAO platform to proteins in the aforementioned clusters. The results of this analysis are summarized in Figure 8, which clearly shows that all these protein sets are characterized by the presence of significant levels of intrinsic disorder. In fact, in all these clusters, proteins classified as disordered based on their PPIDR values exceeding the 30% threshold constitute the vast majority, and 41% to 55% are expected to be highly disordered (based on their positions within the red segment of Figure 8A). Furthermore, from 47.5% to 65.5% of proteins in these clusters are located outside the quadrant Q1 and therefore contain significant levels of intrinsic disorder (see Figure 8B). 

### 3.4. The Roles of Intrinsically Disordered Host Proteins in Viral Immune Evasion and Pathogenesis Enhancement

Now, we are going to focus on how the rabies virus exploited the structural chaos associated with the entrapped host proteins (i.e., their high intrinsic disorder status) to its own benefit. The incorporation of host proteins within viral particles helps them evade immunity and antiviral resistance, and eventually results in the enhancement of viral pathogenesis [21]. These functions can be associated with the intrinsic disorder present in these entrapped host proteins. The viruses with incorporated host proteins are less recognizable by the host immune system, and the antigens of the incorporated host proteins can mask the viral antigens normally recognizable through the immune system [21,104]. Because of the masking of the viral antigens by host proteins, host antibodies cannot efficiently detect the viral particles to successfully eliminate them [21,105]. Furthermore, because of this mimicry, the immune system can get confused, and the effort of finding the mimicking viral particles can sometimes trigger autoimmunity, where the host immune system cells start attacking their own healthy cells, leading to the tissue damage [105,106,107]. 

Viruses can also exploit host receptors to enter various host cells effectively, which not only enhances their transmission rate but also increases the range of cell types a virus can infect [108], and the addition of the host proteins to the viral particles can enhance this ability of the virus [109]. Molecular mimicry also helps viruses to evade antiviral drugs, making the development of antiviral drugs more complicated. Because these drugs are designed to attack unique viral particles without harming healthy host cells [110], the incorporation of host proteins in viral particles can make it difficult for the antiviral drugs to distinguish between the host cells and viral particles, leading to the increased toxicity and side effects and less effective therapeutic targeting.

In the context of our study, when we add the intrinsic disorder of these entrapped proteins to the picture, we can say that the scenario becomes even more complicated. As we have mentioned in the Introduction, IDPs/IDRs lack a 3D structure and are highly flexible and adaptable. They can bind to a variety of partners [111] and can facilitate the interaction of viral particles with a wide array of host cells, facilitating viral entry, replication, and overall pathogenesis. The flexible nature of IDPs can also assist viruses to evolve and become more adaptable to their environment. Viruses can manipulate the properties of intrinsically disordered host proteins to escape the environmental pressure created by the host immune system, also making therapeutic strategies more complex. We can hypothesize that these IDPs/IDRs are providing numerous additional functional and evolutionary benefits to the virus.

In short, we can target the interactions of host IDPs/IDRs with the virus to disrupt the viral life cycle. Understanding the roles of host IDPs/IDRs in the life cycle of viruses can open new lines of research to develop more effective antiviral therapeutic strategies.

## 4. Conclusions

The bioinformatics analysis performed on the host proteins incorporated within the rabies viruses offers significant findings regarding the role of host intrinsic disorder in the life cycles of rabies viruses. 

Out of 47 host proteins that are entrapped in the viral particles, most were predicted as noticeably disordered. In fact, 40.4% of these proteins were predicted as moderately disordered (are characterized by 10% ≤ PPIDR_VSL2_ < 30% and/or 0.15 ≤ ADS_VSL2_ < 0.5), whereas 55.4% of the 47 host proteins were anticipated to be highly disordered (PPDR _VSL2_ ≥ 30% and/or ADS _VSL2_ ≥ 0.5). Based on the results of the PONDR^®^ VSL2-based disorder analysis, 11 proteins were predicted to be mostly disordered, since they were shown to have PPIDR values exceeding 50% and ADS values exceeding 0.5. A detailed computational analysis of the five most disordered host proteins entrapped in the RABV particles, Neuromodulin, Chmp4b, DnaJB6, Vps37B, and Wasl, revealed several important roles that intrinsic disorder can play in the functionality of these proteins. It is also very likely that intrinsic disorder of the host proteins entrapped in the viral particles could be playing essential roles in the pathogenicity of the viruses, modulating their mechanisms of immune evasion, promoting the development of antiviral drug resistance, and thereby contributing to viral adaptability and evolution. 

This study has several obvious limitations. For example, it is not clear at the moment if all the RABV particles produced by an infected cell contain similar quantities of entrapped host proteins or whether the arsenal of the host proteins incorporated into the RABV particles can be influenced by the type of infected cells, where the virions are produced. It is also not clear how different sets of the virion-entrapped host proteins would be in different species infected by the RABV. Another important question is related to understanding the roles of such entrapped host proteins in the inter-species RABV infectivity (e.g., how the dog proteins entrapped in the RABV virions produced in an infected dog would affect a human bitten by said RABV-infected dog). Furthermore, it is not clear how one can use the virion-associated host proteins to experimentally infer the virus–host protein interactions in infected cells. Therefore, subsequent analyses are required to better understand the roles of intrinsic disorder of the host proteins entrapped in the RABV in the virus’s life cycle and pathogenicity. 

## Figures and Tables

**Table 1:** Functional enrichment of the intra-set PPI network of the 47 mouse proteins entrapped in RABV particles.

ID	Description	*p*-Value
**Biological Process (Gene Ontology)**		
GO:0051128	Regulation of cellular component organization	4.73 × 10^−14^
GO:0051049	Regulation of transport	1.28 × 10^−12^
GO:0032879	Regulation of localization	4.85 × 10^−12^
GO:0051050	Positive regulation of transport	8.84 × 10^−12^
GO:0008104	Protein localization	8.84 × 10^−12^
**Molecular Function (Gene Ontology)**		
GO:0044877	Protein-containing complex binding	1.53 × 10^−07^
GO:0005515	Protein binding	3.76 × 10^−07^
GO:0003925	G protein activity	6.11 × 10^−05^
GO:0005488	Binding	6.56 × 10^−05^
GO:0019904	Protein domain-specific binding	0.00014
**Cellular Component (Gene Ontology)**		
GO:0031982	Vesicle	8.04 × 10^−13^
GO:0042470	Melanosome	1.16 × 10^−12^
GO:0031410	Cytoplasmic vesicle	5.80 × 10^−12^
GO:0005829	Cytosol	1.83 × 10^−11^
GO:0005768	Endosome	2.11 × 10^−11^

**Table 2:** Functional enrichment of the PPI networks centered at the most disordered proteins: neuromodulin, Chmp4b, DnaJB6, Vps37B, and Wasl.

Protein	ID	Description	*p*-Value
Neuromodulin	**Biological Process (Gene Ontology)**		
	GO:0022008	Neurogenesis	2.34 × 10^−52^
	GO:0007399	Nervous system development	2.34 × 10^−52^
	GO:0048699	Generation of neurons	1.74 × 10^−48^
	GO:0030182	Neuron differentiation	7.50 × 10^−47^
	GO:0048666	Neuron development	2.92 × 10^−43^
	**Molecular Function (Gene Ontology)**		
	GO:0005515	Protein binding	1.35 × 10^−25^
	GO:0005488	Binding	6.32 × 10^−18^
	GO:0042802	Identical protein binding	1.38 × 10^−10^
	GO:0005102	Signaling receptor binding	5.78 × 10^−10^
	GO:0044877	Protein-containing complex binding	9.88 × 10^−10^
	**Cellular Component (Gene Ontology)**		
	GO:0030424	Axon	2.24 × 10^−59^
	GO:0036477	Somatodendritic compartment	3.44 × 10^−50^
	GO:0043005	Neuron projection	3.44 × 10^−50^
	GO:0044297	Cell body	2.06 × 10^−48^
	GO:0043025	Neuronal cell body	2.87 × 10^−46^
Chmp4b	**Biological Process (Gene Ontology)**		
	GO:0043162	Ubiquitin-dependent protein catabolic process via the multivesicular body sorting pathway	6.73 × 10^−43^
	GO:0071985	Multivesicular body sorting pathway	1.62 × 10^−33^
	GO:0007034	Vacuolar transport	2.43 × 10^−33^
	GO:0032509	Endosome transport via multivesicular body sorting pathway	8.11 × 10^−33^
	GO:0045324	Late endosome to vacuole transport	5.55 × 10^−32^
	**Molecular Function (Gene Ontology)**		
	GO:0005212	Structural constituent of eye lens	4.36 × 10^−20^
	GO:0005198	Structural molecule activity	1.15 × 10^−18^
	GO:0005200	Structural constituent of cytoskeleton	8.50 × 10^−15^
	GO:0005525	GTP binding	3.97 × 10^−09^
	GO:0044389	Ubiquitin-like protein ligase binding	4.28 × 10^−07^
	**Cellular Component (Gene Ontology)**		
	GO:0036452	ESCRT complex	3.59 × 10^−45^
	GO:0005768	Endosome	7.27 × 10^−29^
	GO:0010008	Endosome membrane	3.37 × 10^−28^
	GO:0005770	Late endosome	5.21 × 10^−27^
	GO:0031902	Late endosome membrane	1.09 × 10^−26^
DnaJB6	**Biological Process (Gene Ontology)**		
	GO:0006457	Protein folding	6.61 × 10^−43^
	GO:0061077	Chaperone-mediated protein folding	8.46 × 10^−27^
	GO:0042026	Protein refolding	1.27 × 10^−25^
	GO:0035966	Response to topologically incorrect protein	1.27 × 10^−20^
	GO:0006986	Response to unfolded protein	2.60 × 10^−20^
	**Molecular Function (Gene Ontology)**		
	GO:0051082	Unfolded protein binding	1.35 × 10^−40^
	GO:0044183	Protein folding chaperone	3.88 × 10^−38^
	GO:0140662	ATP-dependent protein folding chaperone	2.63 × 10^−31^
	GO:0031072	Heat shock protein binding	6.85 × 10^−24^
	GO:0051087	Chaperone binding	1.15 × 10^−18^
	**Cellular Component (Gene Ontology)**		
	GO:0005737	Cytoplasm	8.10 × 10^−14^
	GO:0101031	Chaperone complex	1.59 × 10^−13^
	GO:0005829	Cytosol	1.45 × 10^−11^
	GO:0005759	Mitochondrial matrix	2.49 × 10^−10^
	GO:0005739	Mitochondrion	4.02 × 10^−09^
Vps37B	**Biological Process (Gene Ontology)**		
	GO:0043162	Ubiquitin-dependent protein catabolic process via the multivesicular body sorting pathway	1.09 × 10^−52^
	GO:0007034	Vacuolar transport	2.94 × 10^−45^
	GO:0071985	Multivesicular body sorting pathway	3.42 × 10^−41^
	GO:0032509	Endosome transport via multivesicular body sorting pathway	9.64 × 10^−40^
	GO:0045324	Late endosome to vacuole transport	2.43 × 10^−38^
	**Molecular Function (Gene Ontology)**		
	GO:0043130	Ubiquitin binding	3.22 × 10^−11^
	GO:0005515	Protein binding	2.21 × 10^−06^
	GO:0031386	Protein tag	4.31 × 10^−05^
	GO:0019904	Protein domain-specific binding	4.37 × 10^−05^
	GO:0090541	MIT domain binding	0.00019
	**Cellular Component (Gene Ontology)**		
	GO:0036452	ESCRT complex	9.78 × 10^−58^
	GO:0010008	Endosome membrane	8.71 × 10^−42^
	GO:0031902	Late endosome membrane	7.17 × 10^−40^
	GO:0005770	Late endosome	3.41 × 10^−39^
	GO:0005768	Endosome	1.41 × 10^−37^
Wasl	**Biological Process (Gene Ontology)**		
	GO:0030029	Actin filament-based process	6.73 × 10^−119^
	GO:0030036	Actin cytoskeleton organization	1.35 × 10^−117^
	GO:0007010	Cytoskeleton organization	2.58 × 10^−96^
	GO:0032956	Regulation of actin cytoskeleton organization	1.33 × 10^−91^
	GO:0032970	Regulation of actin filament-based process	2.26 × 10^−90^
	**Molecular Function (Gene Ontology)**		
	GO:0003779	Actin binding	1.01 × 10^−71^
	GO:0008092	Cytoskeletal protein binding	1.68 × 10^−71^
	GO:0005515	Protein binding	7.97 × 10^−48^
	GO:0051015	Actin filament binding	3.00 × 10^−40^
	GO:0044877	Protein-containing complex binding	3.18 × 10^−34^
	**Cellular Component (Gene Ontology)**		
	GO:0005856	Cytoskeleton	4.13 × 10^−75^
	GO:0031252	Cell leading edge	2.82 × 10^−74^
	GO:0015629	Actin cytoskeleton	3.67 × 10^−73^
	GO:0042995	Cell projection	2.16 × 10^−63^
	GO:0030027	Lamellipodium	1.97 × 10^−55^

**Table 3:** Localization of ELMs (eukaryotic linear motifs) within the droplet-promoting regions, aggregation hotspots and MoRFs of mouse neuromodulin (UniProt ID: P06837). For additional information, see Supplementary Table S8.

Region Type	Region Range	ELM ID	Position
Droplet-promoting region	52–277	LIG_PDZ_Class_3	222–227
		LIG_WD40_WDR5_VDV_2	219–222
			218–222
			215–222
			155–161
			154–161
			133–137
			132–137
			131–137
			96–99
			95–99
			64–66
			58–64
		MOD_GlcNHglycan	209–212
			132–135
			127–130
			85–88
			84–88
		MOD_SUMO_rev_2	203–207
			200–207
			198–207
			196–201
			193–201
			192–201
			191–201
			154–159
			149–159
			122–126
			118–126
		CLV_C14_Caspase3-7	197–201
		DOC_USP7_MATH_1	207–211
			190–194
			119–123
		MOD_CK2_1	190–196
			142–148
		MOD_GSK3_1	186–193
			135–142
		MOD_PIKK_1	190–196
		LIG_TRAF6_MATH_1	184–192
		DOC_WW_Pin1_4	169–174
			139–144
			93–98
		MOD_ProDKin_1	169–175
			139–145
			93–99
		DOC_USP7_UBL2_3	153–157
		MOD_SUMO_for_1	152–155
			97–100
			25–28
		MOD_CK1_1	142–148
			128–134
			86–92
		LIG_BIR_III_2	118–122
		MOD_Plk_2-3	107–113
		MOD_CDK_SPK_2	93–98
MoRF	102–109	MOD_Plk_2-3	107–113
MoRF	58–81	LIG_WD40_WDR5_VDV_2	58–64
			63–66
Aggregation hotspot	52–66	LIG_WD40_WDR5_VDV_2	58–64
			63–66
MoRF	1–9	LIG_UBA3_1	1–9
		LIG_FHA_1	6–12
		MOD_PKA_2	5–11
		CLV_NRD_NRD_1	6–8
		CLV_PCSK_KEX2_1	6–8
		TRG_ER_diArg_1	5–7
		DEG_Nend_Nbox_1	1–3

**Table 4:** Functional enrichment of the intra-set PPI network of the 11 most disordered mouse proteins found in RABV particles.

ID	Description	*p*-Value
**Biological Process (Gene Ontology)**		
GO:0008104	Protein localization	0.00017
GO:0051641	Cellular localization	0.00017
GO:0019076	Viral release from host cell	0.0011
GO:0043162	Ubiquitin-dependent protein catabolic process via the multivesicular body sorting pathway	0.0013
GO:0016192	Vesicle-mediated transport	0.0014
**Molecular Function (Gene Ontology)**		
GO:0005515	Protein binding	0.0470
**Cellular Component (Gene Ontology)**		
GO:0036452	ESCRT complex	0.00071
GO:0031410	Cytoplasmic vesicle	0.0011
GO:0005768	Endosome	0.0014
GO:0005829	Cytosol	0.0048
GO:0000813	ESCRT I complex	0.0052

**Table 5:** Functional enrichment of the PPI network centered at the 11 most disordered mouse proteins found in the RABV particle, as well as its three clusters.

Network	ID	Description	*p*-Value
Global network	**Biological Process (Gene Ontology)**		
	GO:0016192	Vesicle-mediated transport	3.41 × 10^−80^
	GO:0051641	Cellular localization	3.41 × 10^−80^
	GO:0051128	Regulation of cellular component organization	3.22 × 10^−76^
	GO:0008104	Protein localization	2.85 × 10^−71^
	GO:0051649	Establishment of localization in cell	1.38 × 10^−65^
	**Molecular Function (Gene Ontology)**		
	GO:0005515	Protein binding	1.34 × 10^−77^
	GO:0019904	Protein domain-specific binding	8.56 × 10^−46^
	GO:0008092	Cytoskeletal protein binding	1.06 × 10^−39^
	GO:0005484	SNAP binding	2.99 × 10^−38^
	GO:0005488	Binding	5.52 × 10^−36^
	**Cellular Component (Gene Ontology)**		
	GO:0031982	Vesicle	1.74 × 10^−73^
	GO:0031410	Cytoplasmic vesicle	6.16 × 10^−66^
	GO:0030054	Cell junction	6.16 × 10^−66^
	GO:0042995	Cell projection	3.74 × 10^−64^
	GO:0005488	Cytoplasm	3.74 × 10^−64^
Cluster 1	**Biological Process (Gene Ontology)**		
	GO:0005515	Protein binding	5.60 × 10^−58^
	GO:0008092	Cytoskeletal protein binding	7.30 × 10^−44^
	GO:0003779	Actin binding	6.12 × 10^−43^
	GO:0019904	Protein domain-specific binding	1.31 × 10^−40^
	GO:0019899	Enzyme binding	1.22 × 10^−38^
	**Molecular Function (Gene Ontology)**		
	GO:0051128	Regulation of cellular component organization	1.80 × 10^−65^
	GO:0030029	Actin filament-based process	1.10 × 10^−53^
	GO:0044087	Regulation of cellular component biogenesis	1.35 × 10^−52^
	GO:0030036	Actin cytoskeleton organization	5.31 × 10^−50^
	GO:0007010	Cytoskeleton organization	5.51 × 10^−48^
	**Cellular Component (Gene Ontology)**		
	GO:0042995	Cell projection	1.61 × 10^−62^
	GO:0120025	Plasma membrane bounded cell projection	2.96 × 10^−59^
	GO:0005829	Cytosol	1.36 × 10^−51^
	GO:0030054	Cell junction	2.26 × 10^−50^
	GO:0005856	Cytoskeleton	6.03 × 10^−48^
Cluster 2	**Biological Process (Gene Ontology)**		
	GO:0043162	Ubiquitin-dependent protein catabolic process via the multivesicular body sorting pathway	5.74 × 10^−45^
	GO:0007034	Vacuolar transport	4.66 × 10^−41^
	GO:0071985	Multivesicular body sorting pathway	4.84 × 10^−37^
	GO:0046755	Viral budding	2.25 × 10^−36^
	GO:0032509	Endosome transport via multivesicular body sorting pathway	5.26 × 10^−36^
	**Molecular Function (Gene Ontology)**		
	GO:0043130	Ubiquitin binding	6.09 × 10^−08^
	GO:0005515	Protein binding	2.69 × 10^−06^
	GO:0004459	L-lactate dehydrogenase activity	4.33 × 10^−06^
	GO:0048306	Calcium-dependent protein binding	9.28 × 10^−06^
	GO:0005543	Phospholipid binding	5.52 × 10^−05^
	**Cellular Component (Gene Ontology)**		
	GO:0036452	ESCRT complex	1.23 × 10^−49^
	GO:0010008	Endosome membrane	6.18 × 10^−43^
	GO:0031902	Late endosome membrane	1.39 × 10^−38^
	GO:0005768	Endosome	8.24 × 10^−38^
	GO:0030659	Cytoplasmic vesicle membrane	1.80 × 10^−34^
Cluster 3	**Biological Process (Gene Ontology)**		
	GO:0016192	Vesicle-mediated transport	4.17 × 10^−59^
	GO:0061025	Membrane fusion	6.79 × 10^−59^
	GO:0006906	Vesicle fusion	1.63 × 10^−53^
	GO:0051649	Establishment of localization in cell	5.79 × 10^−46^
	GO:0016050	Vesicle organization	7.23 × 10^−44^
	**Molecular Function (Gene Ontology)**		
	GO:0000149	SNARE binding	1.15 × 10^−72^
	GO:0005484	SNAP receptor activity	5.11 × 10^−70^
	GO:0030674	Protein-macromolecule adaptor activity	6.13 × 10^−46^
	GO:0019905	Syntaxin binding	4.45 × 10^−43^
	GO:0017075	Syntaxin-1 binding	7.64 × 10^−16^
	**Cellular Component (Gene Ontology)**		
	GO:0031201	SNARE complex	2.90 × 10^−91^
	GO:0030133	Transport vesicle	4.17 × 10^−43^
	GO:0070382	Exocytic vesicle	3.76 × 10^−41^
	GO:0008021	Synaptic vesicle	2.50 × 10^−40^
	GO:0098796	Membrane protein complex	4.14 × 10^−40^

**Figure A1:**
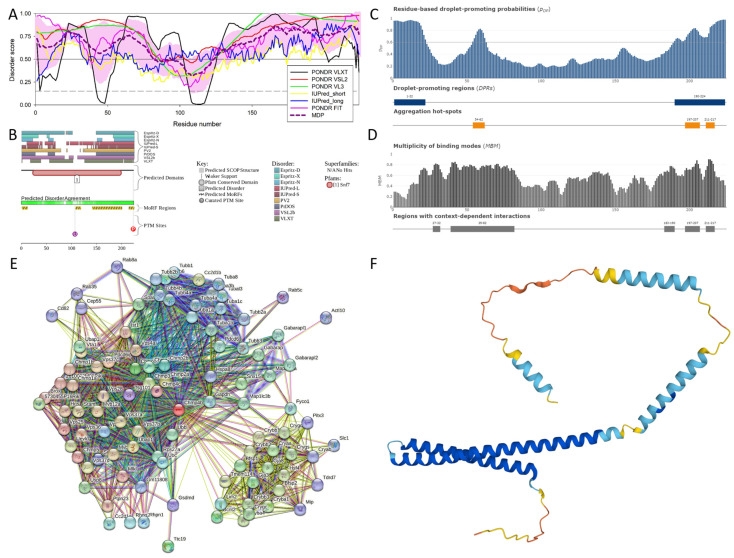
Functional disorder analysis of mouse Chmp4b (UniProt ID: Q9D8B3). (**A**) Per-residue disorder profile generated using RIDAO. (**B**) Functional disorder profile generated using D^2^P^2^. (**C**) Per-residue LLPS potential as estimated using FuzDrop, demonstrating the tendency of each residue to promote droplet formation. (**D**) Multiplicity of binding modes plot generated using FuzDrop. (**E**). The PPI network was generated utilizing STRING by adjusting the value of the maximum number of interactors at 500. (**F**) The 3D structural model generated using AlphaFold. The structure is colored according to the per-residue model confidence score, ranging from orange (very low confidence p_LDDT_ < 50) to blue (very high confidence p_LDDT_ > 90), respectively.

**Figure A2:**
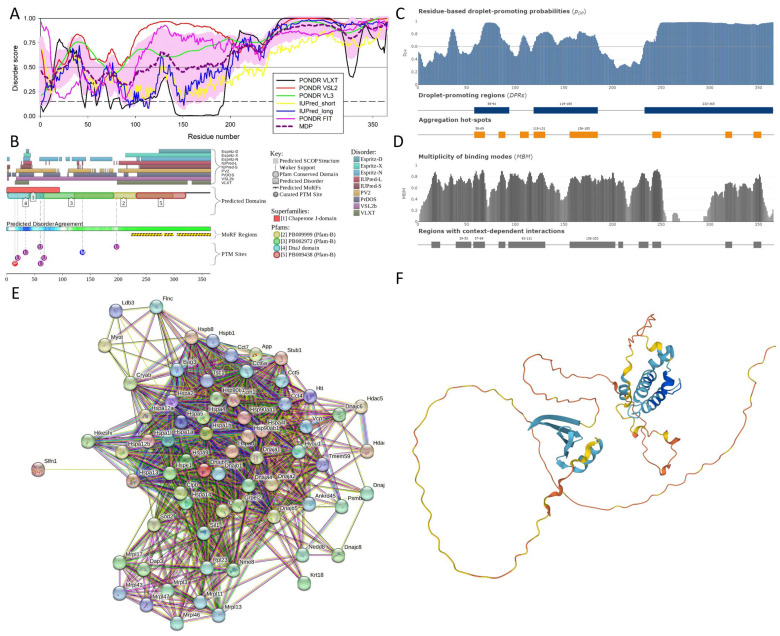
Functional disorder analysis of protein DnaJ homolog subfamily B member 6 (UniProt ID: O54946). (**A**) RIDAO-generated per-residue disorder profile. (**B**) Disorder-based functionality evaluated using D^2^P^2^. (**C**) Per-residue LLPS potential as estimated using FuzDrop, demonstrating the tendency of each residue to promote droplet formation. (**D**) Multiplicity of binding modes plot generated using FuzDrop. (**E**) The PPI network generated utilizing STRING by adjusting the value of the maximum number of interactors at 500. (**F**) The 3D structural model generated using AlphaFold. The structure is colored according to the per-residue model confidence score (p_LDDT_), with fragments of the structure with very low (p_LDDT_ < 50), low (70 > p_LDDT_ > 50, high (90 > p_LDDT_ > 70), and very high confidence (p_LDDT_ > 90) being shown by orange, yellow, cyan, and blue colors, respectively.

**Figure A3:**
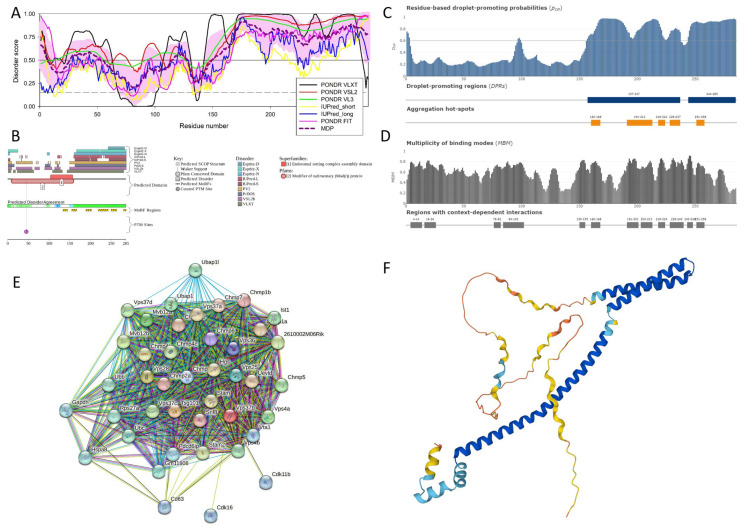
Functional disorder analysis of protein Vps37b (UniProt ID: Q8R0J7). (**A**) Per-residue disorder profile generated using the RIDAO platform. (**B**) Functional disorder profile generated using the D^2^P^2^ database. (**C**) Per-residues droplet formation propensity generated using FuzDrop. (**D**) Multiplicity of binding modes plot generated using FuzDrop. (**E**) Protein–protein interaction network generated for this protein utilizing STRING database. (**F**) The 3D structural model predicted by AlphaFold. The structure is colored according to the per-residue model confidence score, ranging from orange (p_LDDT_ < 50) to blue (p_LDDT_ > 90).

**Figure A4:**
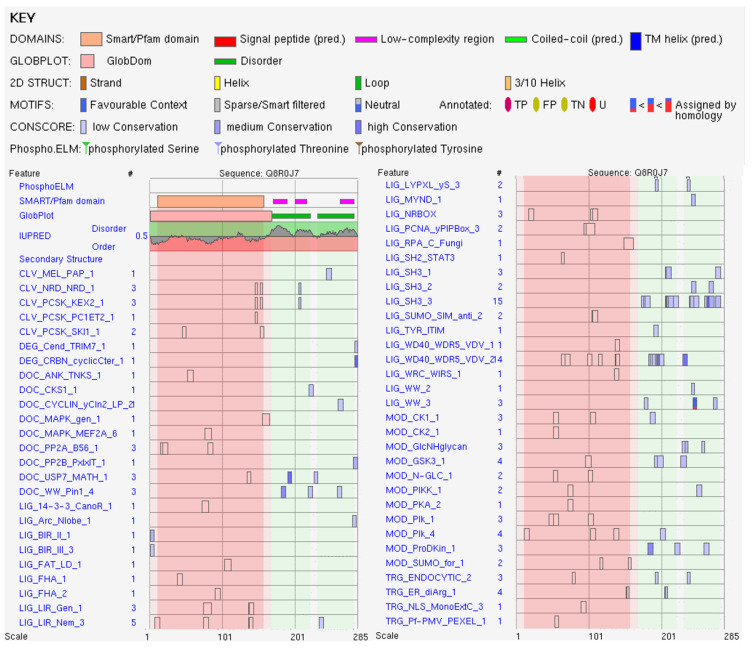
Distribution of ELMs (short linear functional motifs) within the sequence of the mouse Vps37B protein. Refer to the additional information provided in Supplementary Table S11.

**Figure A5:**
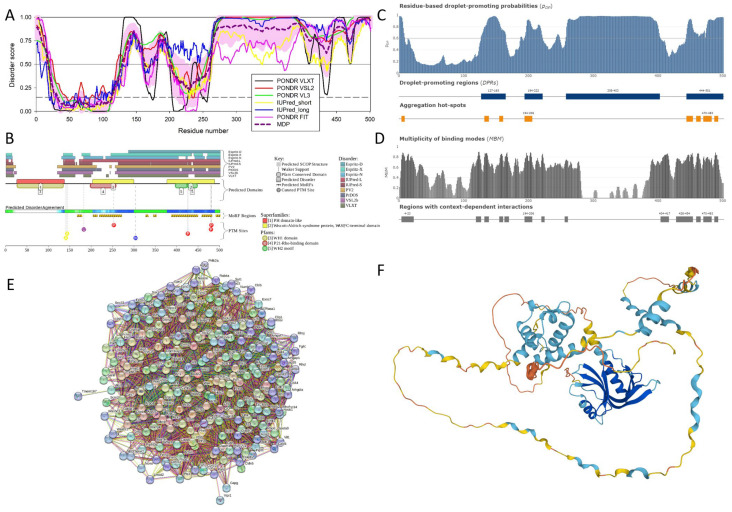
Functional disorder analysis of mouse protein Wasl (UniProt ID: Q91YD9). (**A**) Multiparametric intrinsic disorder profile generated using RIDAO. (**B**) D^2^P^2^-generated functional disorder profile. (**C**) Residue-based LLPS propensity. (**D**) Multiplicity of binding modes plot. (**E**) Wasl-centered PPI network generated utilizing STRING database by adjusting the value of the maximum number of interactors to 500. (**F**) The 3D structural model as predicted by AlphaFold. The structure is colored according to the per-residue model confidence score, ranging from orange (very low confidence, p_LDDT_ < 50) to blue (very high confidence, p_LDDT_ > 90).

**Figure A6:**
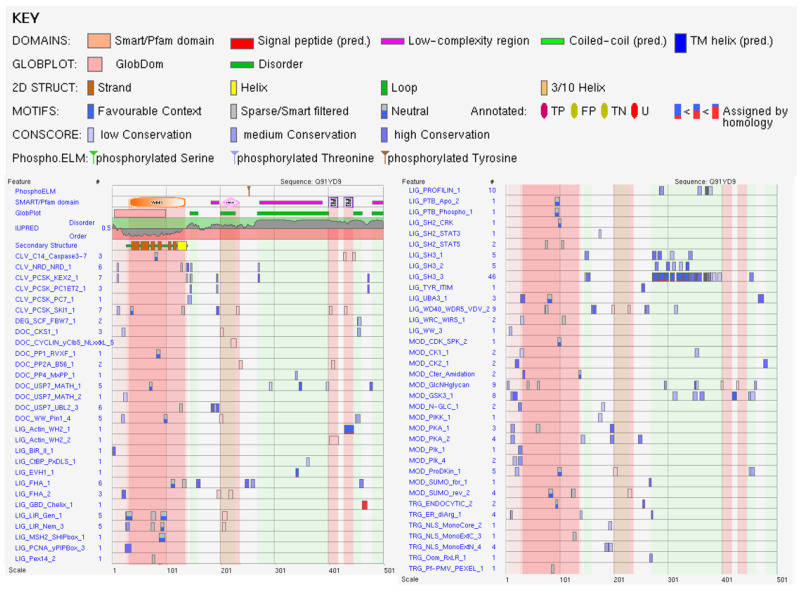
Distribution of ELMs (short linear functional motifs) within the sequence of the mouse Wasl protein (UniProt ID: Q91YD9). For additional information, see Supplementary Table S12.

## Data Availability

The data are contained within the article and the Supplementary Materials.

## Supplementary Materials

The following supporting information can be downloaded at: https://www.mdpi.com/article/10.3390/v16060916/s1, Supplementary materials S1: Brief description of the protocol utilized by Zhang and colleagues for the analysis of the host proteins entrapped within the RABV nanoparticles; File S1. Amino acid sequences of proteins analyzed in this study; Table S1. Functional enrichment data for 11 highly disordered host proteins focusing on gene ontology, highlighting biological processes; Table S2. Functional enrichment data for 11 highly disordered host proteins focusing on their individual gene ontology, highlighting molecular functions; Table S3. Functional enrichment data for 11 highly disordered host proteins focusing on their individual gene ontology, highlighting cellular components; Table S4. Multifactorial analysis of intrinsic disorder predisposition of mouse proteins entrapped in RABV particles; Table S5. Functional enrichment of the intra-set PPI network of the 47 mouse proteins entrapped in RABV particles: biological processes; Table S6. Functional enrichment of the intra-set PPI network of the 47 mouse proteins entrapped in RABV particles: molecular functions; Table S7. Functional enrichment of the intra-set PPI network of the 47 mouse proteins entrapped in RABV particles: cellular components; Table S8. Localization of ELMs (eukaryotic linear motifs) within the droplet-promoting regions, aggregation hotspots, and MoRFs of mouse neuromodulin (UniProt ID: P06837); Table S9. Distribution of ELMs (Eukaryotic Linear Motifs) in droplet-promoting regions, aggregation hotspots, regions with multiplicity of binding modes, and MoRFs (molecular recognition features) of the protein Chmp4b (UniProt ID: Q9D8B3); Table S10. Distribution of ELMs (eukaryotic linear motifs) in droplet-promoting regions, aggregation hotspots, regions with multiplicity of binding modes, and MoRFs (molecular recognition features) of protein DnaJ homolog subfamily B member 6 (UniProt ID: O54946); Table S11. Distribution of ELMs (eukaryotic linear motifs) in droplet-promoting regions, aggregation hotspots, regions with multiplicity of binding modes, and MoRFs (molecular recognition features) of Vps37B protein (UniProt ID: Q8R0J7); Supplementary Table S12. Distribution of ELMs (short linear functional motifs) within the sequence of the mouse Wasl protein (UniProt ID: Q91YD9). Figure S1. FuzDrop results (a,b), RIDAO results (c), D2P2 Results (d), STRING–generated PPI network (e), and AlphaFold Structure (f) for protein Pascin2 (UniProt ID: Q9WVE8). Figure S2. FuzDrop results (a, b), RIDAO results (c), D2P2 Results (d), STRING–generated PPI network (e), and AlphaFold Structure (f) for protein Ddx3x (UniProt ID: Q62167). Figure S3. FuzDrop (a,b), D2P2 (c), and RIDAO (d) results for protein Snx18 (UniProt ID:Q9Z1R2). Figure S4. FuzDrop (a,b), D2P2 (c), and RIDAO (d) results for protein Tsg101 (UniProt ID: Q61187). Figure S5. FuzDrop (a,b), D2P2 (c), and RIDAO (d) results for protein Ezr (UniProt ID: P26040).

## Appendix A

### Appendix A.1. Functional Intrinsic Disorder in the Most Disordered Mouse Proteins, Chmp4b, DnaJB6, Vps37B, and Wasl, Found in the Rabies Virus

#### Appendix A.1.1. Charged Multivesicular Body Protein 4b (Chmp4b, UniProt ID: Q9D8B3)

Charged multivesicular body protein 4b, also known as chromatin-modifying protein 4b (Chmp4b), is an essential component of the ESCRT-III (endosomal sorting complex for transport III) system that plays a significant role in the process of endosomal sorting in the cells [112]. There are five specific ESCRT complexes (ESCRT-0, -I, -II, -III, and the Vps4 complex) characterized by specific functions, such as interaction with ubiquitylated membrane proteins, membrane deformation, and abscission, all related to the topologically unique membrane bending and scission reaction that occur away from the cytoplasm [113]. These ESCRT-driven activities are crucial for the processing of the multivesicular body (MVB) pathway, cytokinesis, and HIV budding [113]. It has been indicated that the ESCRT-0, -I, and -II complexes represent stable protein ensembles in the cytoplasm, whereas ESCRT-III complex, which includes four core subunits (Vps20/CHMP6, Snf7/CHMP4(A–C), Vps24/CHMP3, and Vps2/CHMP2(A,B)) and three assessor proteins (Did2/CHMP1(A,B), Vps60/CHMP5, and Ist1), is transiently formed on endosomes [113].

The point to be noted is that ESCRT machinery including ESCRT-III is hijacked by HIV in humans, which is critical for the release of HIV from the infected host cells [114]. Defects in the ESCRT machinery, which include Chmp4b, play a role in the pathogenesis of neurodegenerative diseases because of their function of clearing out the misfolded proteins from the cells.

Chmp4b is a 224-residue-long protein with a molecular weight of 25KDa and is encoded by the *Chmp4b* gene. Post-translational modifications can be found at residues 2 (N-acetylserine), 6 (N6-acetyllysine), 14 (N6-acetyllysine), 14 (phosphoserine), and 223 (phosphoserine), and the Snf7 domain found between residues 24 and 199 is involved in the multiple functions of the ESCRT machinery. The coiled-coil domain is located between residues 23 and 183, whereas the N-terminal region (residues 2–153) is involved in intramolecular interactions with the C-terminal region (residues 154–224). It has been indicated that core subunits of the ESCRT-III complex potentially have a similar structural organization, where the N-terminal region “consists of two helices (α1, α2) that form a 7 nm hairpin structure important for membrane binding and homo- or hetero-dimerization. In the cytoplasm, the negatively charged C-terminal region (α5 and α6) folds back on the positively charged N-terminal hairpin, which confers an autoinhibitory mechanism that stabilizes the inactive monomers” [113].

The per-residue intrinsic disorder predisposition graph generated based on the outputs of the RIDAO algorithm is shown in Figure A1A. It clearly shows that the mouse Chmp4b is predicted to be a highly disordered protein. In fact, the PPIDR values predicted for this protein by various predictors included in RIDAO are high: PONDR^®^ VLXT: 73.21%; PONDR^®^ VSL2B: 92.41%; PONDR^®^ VL3: 83.48%; PONDR^®^ FIT: 83.48%; IUPred_Short: 55.36%; and IUPred_Long: 78.12%. The average of all these values is 81.70%, which indicates that this protein is highly disordered. Most of the disordered residues were observed in the N- and C-terminal tails of the protein (residues 1–81 and 124–224).

Further proof of the highly disordered nature of mouse Chmp4b is given by the outputs of the D^2^P^2^ platform (see Figure A1B), which provides a comprehensive functional disorder prediction profile of the protein. The predicted disorder agreement is shown in the green-colored bars just below the predicted Snf7domain. MoRF regions depicted below the disorder prediction are residues 1–12, 108–118, 141–200, and 214–244. These are the regions that undergo a disorder-to-order transition upon binding with their respective partners [115]. Two PTM sites indicated in the D^2^P^2^ profile are at Lys107 (ubiquitination) and at Ser223 (phosphorylation).

The results of the FuzDrop-based analysis of mouse Chmp4b are summarized in Figure A1C,D. Figure A1C illustrates the droplet-promoting probabilities for each residue. Although the p_LLPS_ value of 0.5154 predicted for this protein is below the 0.60 threshold, Chmp4b is predicted to have two droplet-promoting regions (residues 1–22 and 190–224), i.e., regions with pDP values above the 0.6 threshold, indicating that this protein can serve as a droplet client. The aggregation hotspots (i.e., regions that have a high tendency to aggregate, and therefore can also contribute to the pathogenesis of neurological disorders) are found at residues 54–62, 197–207, and 211–217. The multiplicity of binding modes graph is displayed in Figure A1D, revealing the tendency of residues to engage in multiple interactions with various partners.

A high MBM predicts that several regions (residues 27–32, 39–82, 183–190, 197–207, and 211–217) can take part in multiple interactions, aiding the liquid–liquid phase separation process, and be involved in context-dependent interactions (see Figure A1D). These regions consist of residues that behave differently depending on the context of their cellular environment.

Figure A1E depicts the Chmp4b-centered PPI network that includes 100 proteins interconnected through 1341 edges (edges represent the interactions between proteins). This observed value of edges is much greater than the expected number of edges of 176. The average node degree (which is the average number of connections per protein) predicted for this network is 26.8, and its average local clustering coefficient is 0.806. The PPI enrichment *p*-value is <1.0 × 10^−16^, suggesting that proteins in this Chmp4b-centered PPI network have more interactions among themselves than what would be expected for a random set of proteins of the same size and degree distribution drawn from the genome. Most enriched biological processes, molecular functions, and cellular components of the members of this network are listed in Table 2.

Lastly, Figure A1F represents the 3D model generated for mouse Chmp4b using Attention AE: rAlphaFold. Surprisingly, although the predicted structure mostly represents a set of disjoined α-helices that do not form a core, this model is characterized by a relatively high confidence of above 70% (the structure mostly consists of structural elements colored in cyan (high confidence, 90 > p_LDDT_ > 70) and blue (very high confidence, p_LDDT_ > 90)). As it was already indicated, long α-helical segments cannot exist in isolation. Therefore, it is very likely that the structure predicted by AlphaFold corresponds to the bound form of the protein. It is known that the remodeling of the membrane in abscission is caused by the polymerization of ESCRT-III components, which are soluble in a monomeric autoinhibited state but assemble into membrane-bound filaments with crucial roles in membrane fission, when this autoinhibition is relieved [116]. Therefore, it is likely that the formation of ESCRT-III filaments is accompanied by the disorder-to-order transition of the core subunits of this complex.

To shed more light on the potential functionality of various regions identified in mouse Chmp4b, we analyzed these proteins using the ELM platform. The results of this analysis are listed in Table A1.

**Table A1:** Distribution of ELMs (eukaryotic linear motifs) in droplet-promoting regions, aggregation hotspots, regions with multiplicity of binding modes, and MoRFs (molecular recognition features) of the protein Chmp4b (UniProt ID: Q9D8B3). The table summarizes the ELMs mapped onto these regions, suggesting the potential functional role of these motifs. For additional information, see the Supplementary Table S9.

Region Type	Region Range	ELM ID	Position
MoRF	1–12	LIG_BIR_II_1	1–5
		LIG_LIR_Nem_3	2–7
		LIG_Pex14_2	4–8
Droplet-promoting region	1–22	LIG_BIR_II_1	1–5
		LIG_LIR_Nem_3	2–7
		LIG_Pex14_2	4–8
		DOC_WW_Pin1_4	18–23
		MOD_ProDKin_1	18–24
		LIG_FHA_2	19–25
Region with multiplicity of binding modes	27–32	MOD_PKA_2	29–35
Region with multiplicity of binding modes	39–82	MOD_SUMO_rev_2	41–47
		TRG_NLS_Bipartite_1	55–75
		DOC_USP7_UBL2_3	56–60
		CLV_PCSK_PC1ET2_1	62–64
		TRG_NLS_MonoExtN_4	70–75
		TRG_NLS_MonoCore_2	69–74
			70–75
		TRG_NLS_MonoExtC_3	69–74
			70–75
Aggregation hotspot	54–62	DOC_USP7_UBL2_3	56–60
		CLV_PCSK_PC1ET2_1	62–64
MoRF	108–118	LIG_SH2_STAP1	111–115
		LIG_WD40_WDR5_VDV_2	111–115
		CLV_PCSK_SKI1_1	114–118
MoRF	141–200	MOD_GSK3_1	143–150
			181–188
		DOC_PP1_RVXF_1	149–156
		LIG_Pex14_2	155–159
		LIG_WD40_WDR5_VDV_2	161–168
			162–168
			163–168
			177–183
		CLV_PCSK_SKI1_1	178–182
		TRG_Pf-PMV_PEXEL_1	178–182
		LIG_SUMO_SIM_par_1	179–184
		MOD_CK2_1	181–187
		MOD_GlcNHglycan	182–186
			183–186
		LIG_FHA_1	186–192
		LIG_SH3_3	186–192
			189–195
			194–200
			197–203
		DOC_USP7_MATH_1	198–202
Region with multiplicity of binding modes	183–190	LIG_WD40_WDR5_VDV_2	177–183
		TRG_Pf-PMV_PEXEL_1	178–182
		LIG_SUMO_SIM_par_1	179–184
		MOD_CK2_1	181–187
		MOD_GlcNHglycan	182–186
			183–186
		LIG_FHA_1	186–192
		LIG_SH3_3	186–192
			189–195
Region with multiplicity of binding modes	197–207	DOC_USP7_MATH_1	198–202
		LIG_SH3_2	200–205
		CLV_PCSK_SKI1_1	202–206
		DOC_USP7_UBL2_3	202–206
Aggregation hotspot	197–207	DOC_USP7_MATH_1	198–202
		LIG_SH3_2	200–205
		CLV_PCSK_SKI1_1	202–206
		DOC_USP7_UBL2_3	202–206
Droplet-promoting region	190–224	LIG_FHA_1	186–192
		LIG_SH3_3	186–192
			189–195
			194–200
			197–203
		DOC_USP7_MATH_1	198–202
		LIG_SH3_2	200–205
		CLV_PCSK_SKI1_1	202–206
		DOC_USP7_UBL2_3	202–206
		LIG_SH3_4	202–209
		TRG_NESrev_CRM1_2	208–217
			209–217
			210–217
			211–217
			212–217
Aggregation hotspot	211–217	TRG_NESrev_CRM1_2	208–217
			209–217
			210–217
			211–217
			212–217
MoRF	214–224	TRG_NESrev_CRM1_2	208–217
			209–217
			210–217
			211–217
			212–217
		MOD_SUMO_rev_2	212–217

The data reported in Table A1 indicate that the intrinsically disordered regions of Chmp4b are involved in the promotion of liquid–liquid phase separation, serving as aggregation hotspots, and acting as MoRFs, and regions with multiplicity of binding modes are heavily enriched in potentially functional short linear motifs.

#### Appendix A.1.2. DnaJ Homolog Subfamily B Member 6 (DNAJB6; UniProt ID: O54946)

DnaJB6 is a 365-residue-long protein with a molecular weight of 99,807 Da and is involved in the cellular response towards stress, and, being a member of the Hsp40 family chaperone family, act as a co-chaperone of Hsp70 [117]. It has a stimulatory effect on the ATPase activity of the heat shock protein Hsp70. DnaJB6’s activity as a co-chaperone indicates its importance in protein folding, repair, and assembly. For example, it plays the role of an endogenous chaperone for huntingtin neuronal protein [117]. Being able to successfully suppress the aggregation and toxicity of polyglutamine-containing, aggregation-prone proteins [118,119], DnaJB6 is designated as the antiamyloid chaperone, which is also capable of binding to the amyloid-β peptide fibrils and inhibiting secondary nucleation [120]. Furthermore, this chaperone is related to the biogenesis of the interphase nuclear pore complex (NPC), binds to phenylalanine–glycine-rich nucleoporins (FG-Nups), and prevents their aggregation in cells and in vitro [121]. Furthermore, it is able to form foci (i.e., likely to phase separate) in close proximity to NPCs [121]. This protein was also shown to play a role in the organization of keratin 8 and 18 (KRT8/KRT18) filaments [122].

The N-terminal half of the protein contains a DnaJ domain (residues 3–69) and contains an Hsp70 interacting region (residues 2–147). The region comprising residues 120–243 has been shown to interact with KRT8 and the C-terminal region (residues 243–365) is expected to be disordered and contains a subregion 273–287 with the compositional bias (enriched in basic and acidic residues). In line with these observations, Figure A2 shows that the mouse DnaJB6 protein contains significant levels of functional intrinsic disorder. Based on the RIDAO-based analyses (see Figure A2A), this protein is characterized by PPIDR values of 50.68% (PONDR^®^ VLXT), 96.44% (PONDR^®^ VSL2B), 89.59% (PONDR^®^ VL3), 76.16% (PONDR^®^ FIT), 43.29% (IUPred_Short), and 52.33% (IUPred_Long). The mean PPIDR value averaged over all these tools is 66.58%. Figure 6A also shows that a highly disordered region was found at the C-terminal region of the protein (residues 253–365).

As per the D^2^P^2^ analysis, the consensus IDRs are found at residues 15–98, 106–188, and 197–365 (Figure A2B). Figure A2B also shows that mouse DnaJB6 contains three MoRFs (residues 223–278, 282–298, and 305–365) and includes several PTMs, such as phosphorylation at Ser15, mono-methylation at Arg136, and ubiquitylation at Lys20, Lys 34, Lys 60, Lys 61, and Lys 67.

Figure A2C shows the FuzDrop-generated profile reflecting the LLPS and droplet formation tendency of the protein. Here, the residues with p_DP_ ≥ 0.6 threshold are expected to have the tendency to promote liquid–liquid phase separation. The p_LLPS_ value of 0.9937 for DNAJB6 is extremely high, significantly exceeding a threshold value of 0.6, indicating that this protein is a droplet driver. This is in a line with the aforementioned capability of DnaJB6 to form foci in the vicinity of NPCs [121]. Figure A2C also shows that in DnaJB6, the droplet-promoting regions are predicted at residues 58–94, 119–185, and 233–365. Aggregations hotspots are found at residues 58–69, 83–90, 105–114, 119–131, 156–185, 241–250, 316–323, and 345–353. Figure A2D portrays a multiplicity of binding modes influenced by cellular contexts such as PTMs and the sub-cellular location of the protein. The residues with an MBM ≥ 0.65 are said to form regions with context-dependent interactions. For DnaJB6, the following regions were predicted to be MBM regions: 14–23, 39–55, 57–69, 83–90, 93–131, 156–203, 206–211,227–237,241–250,316–323, and 345–353.

Figure A2E represents the STRING-generated PPI network of mouse DnaJB6. This network includes 68 interactors and 993 interactions. It is characterized by an average local clustering coefficient of 0.78 and has an average node degree of 29.2. The expected number of edges for the DnaJB6-centerd PPI network is expected to be 209, indicating that the actual network has far more interactions than expected, indicating that the members of this network are involved in the significant number of biological processes. A *p*-value of <1.0 × 10^−16^ suggests that the network we are observing in Figure A2E is statistically significant and cannot be generated by random chance. Most enriched biological processes, molecular functions, and cellular components of the members of this network are listed in Table 2.

The 3D structural model of the protein predicted by AlphaFold, as shown in Figure A2F, has an average per-residue model confidence score (p_LDDT_) of 60.8, indicating an overall low confidence. The AlphaFold-predicted structure also reveals that the C-terminal region of the protein is highly disordered, whereas the N-terminal region includes two structured domains, a mostly α-helical DnaJ domain (residues 1–104) and a mostly β-structural domain (residues 190–234) containing five antiparallel β-strands (residue 190–199, 202–211, 214–221, 224–230, and 233–234), followed by an α-helix (residues 236–245).

Table A2 lists some of the ELMs predicted in mouse DnaJB6 and shows that in line with its high intrinsic disorder status, this protein has a multitude of potential disorder-based functions.

**Table A2:** Distribution of ELMs (eukaryotic linear motifs) in droplet-promoting regions, aggregation hotspots, regions with multiplicity of binding modes and MoRFs (molecular recognition features) of protein DnaJ homolog subfamily B member 6 (UniProt ID: O54946). The table summarizes the ELMs mapped onto these regions, suggesting a potential functional role of these motifs. For additional information, see the Supplementary Table S10.

Region Type	Region Range	ELM ID	Position
Region with multiplicity of binding modes	14–23	DOC_WW_Pin1_4	12–17
		CLV_NRD_NRD_1	23–25
Region with multiplicity of binding modes	39–55	CLV_NRD_NRD_1	43–45
		CLV_PCSK_SKI1_1	44–48
Droplet-promoting region	58–94	LIG_LIR_Nem_3	63–68
		DOC_WW_Pin1_4	83–88
		DOC_PP4_FxxP_1	84–87
Region with multiplicity of binding modes	57–69	TRG_Pf-PMV_PEXEL_1	62–66
		LIG_LIR_Nem_3	63–68
Aggregation hotspot	58–69	TRG_Pf-PMV_PEXEL_1	62–66
		LIG_LIR_Nem_3	63–68
Droplet-promoting region	58–94	TRG_Pf-PMV_PEXEL_1	62–66
		DOC_PP4_FxxP_1	84–87
			94–97
Region with multiplicity of binding modes	83–90	DOC_WW_Pin1_4	83–88
		MOD_ProDKin_1	83–89
		DOC_PP4_FxxP_1	84–87
Aggregation hotspot	83–90	DOC_WW_Pin1_4	83–88
		MOD_ProDKin_1	83–89
		DOC_PP4_FxxP_1	84–87
Region with multiplicity of binding modes	93–131	DOC_PP4_FxxP_1	84–87
			94–97
		CLV_PCSK_SKI1_1	102–106
Aggregation hotspot	105–114	LIG_BRCT_BRCA1_1	111–115
		LIG_AP2alpha_2	109–111
Aggregation hotspot	119–131	DOC_PP4_FxxP_1	116–119
		LIG_AP2alpha_1	116–120
			120–124
		LIG_AP2alpha_2	118–120
		CLV_NRD_NRD_1	127–129
		CLV_PCSK_KEX2_1	127–129
Droplet-promoting region	119–185	DOC_PP4_FxxP_1	116–119
		LIG_AP2alpha_1	116–120
			120–124
		CLV_NRD_NRD_1	127–129
		CLV_PCSK_KEX2_1	127–129
		LIG_Arc_Nlobe_1	148–152
			155–120
		OC_USP7_MATH_1	164–168
		LIG_BRCT_BRCA1_1	177–181
Aggregation hotspot	156–185	LIG_Arc_Nlobe_1	155–159
		DOC_WW_Pin1_4	160–165
		OC_USP7_MATH_1	164–168
		LIG_BRCT_BRCA1_1	177–181
Region with multiplicity of binding modes	156–203	OC_USP7_MATH_1	164–168
		LIG_BRCT_BRCA1_1	177–181
		CLV_PCSK_SKI1_1	202–206
		DOC_USP7_UBL2_3	203–207
Region with multiplicity of binding modes	206–211	CLV_PCSK_SKI1_1	202–206
		DOC_USP7_UBL2_3	203–207
		CLV_PCSK_KEX2_1	207–209
MoRF	223–278	CLV_NRD_NRD_1	245–247
		CLV_PCSK_SKI1_1	226–230
Region with multiplicity of binding modes	227–237	CLV_PCSK_SKI1_1	226–230
Droplet-promoting region	233–365	CLV_NRD_NRD_1	245–247
		DEG_ODPH_VHL_1	253–264
		DOC_USP7_MATH_1	291–295
			293–297
			334–338
		DEG_SCF_FBW7_1	271–278
			273–278
			275–282
			277–282
			287–294
		DOC_USP7_UBL2_3	310–314
			341–345
			348–352
			352–356
			358–362
Region with multiplicity of binding modes	241–250	CLV_NRD_NRD_1	245–247
		DOC_CKS1_1	248–253
Aggregation hotspot	241–250	CLV_NRD_NRD_1	245–247
		DOC_CKS1_1	248–253
MoRF	282–298	DEG_SCF_FBW7_1	275–282
			277–282
			287–294
		DOC_WW_Pin1_4	279–284
			287–292
		DOC_USP7_MATH_1	291–295
			293–297
		LIG_WD40_WDR5_VDV_2	290–295
Region with multiplicity of binding modes	316–323	MOD_CK2_1	312–318
		DOC_ANK_TNKS_1	323–330
Aggregation hotspot	316–323	MOD_CK2_1	312–318
		DOC_ANK_TNKS_1	323–330
Aggregation hotspot	345–353	OC_USP7_UBL2_3	341–345
			348–352
			352–356
		DOC_USP7_UBL2_3	341–345
			348–352
			352–356
		TRG_NLS_Bipartite_1	345–361
			346–261
			347–361
		CLV_NRD_NRD_1	345–347
		CLV_PCSK_KEX2_1	345–347
Region with multiplicity of binding modes	345–353	OC_USP7_UBL2_3	341–345
			348–352
			352–356
		DOC_USP7_UBL2_3	341–345
			348–352
			352–356
		TRG_NLS_Bipartite_1	345–361
			346–261
			347–361
		CLV_NRD_NRD_1	345–347
		CLV_PCSK_KEX2_1	345–347
MoRF	305–365	DOC_USP7_UBL2_3	310–314
			341–345
			348–352
			352–356
			358–362
		OC_USP7_UBL2_3	341–345
			348–352
			352–356
			368–362
		TRG_NLS_Bipartite_1	345–361
			346–261
			347–361
		DOC_USP7_MATH_1	334–338
		CLV_NRD_NRD_1	345–347
		CLV_PCSK_KEX2_1	345–347

Note, Table A2 does not include all ELMs found in mouse DnaJB6, as this protein is predicted to have 57 different ELMs, with many of these being present in multiple copies (there are total of 186 ELM instances in DnaJB6).

#### Appendix A.1.3. Vacuolar Protein Sorting-Associated Protein 37B (Vps37B, UniProt ID: Q8R0J7)

Vps37B, alternatively called ESCRT-I complex subunit Vps37B, is a 285-amino acids long with a molecular mass of 31,056 Da. Vps37B is a component of ESCRT-I complex (endosomal sorting complex required for transport), which is a regulator of the vesicular transport process. As it was already indicated, endosomal sorting complexes required for transport machinery include five complexes with unique but connected functions: ESCRT-0, ESCRT-I, ESCRT-II, ESCRT-III, and the Vps4 complex. Among the many important activities of ancient ESCRT machinery are membrane deformation and scission (budding of the membranes and severing membrane necks from their interface) to form intraluminal vesicles (ILVs) linked to the biogenesis of the multivesicular bodies (MVBs) in endo-lysosomal sorting, as well as the budding of HIV-1 and other viruses from the plasma membranes of infected cells and the membrane abscission step in cytokinesis. Furthermore, these complexes are related to the autophagy, cytokinesis, exovesicle release, and repair of plasma and intracellular membranes as well as enveloped RNA virus budding [123,124,125,126]. ESCRTs are oligomeric complexes that have complementary functions. Major components of the ESCRT-I complex, which is central to all ESCRT pathways and is essential for the MVB sorting of ubiquitylated cargo, are the three core subunits, Tsg101 (Vps23 in *Saccharomyces cerevisiae*), Vps28, one of four Vps37 family members (Vps37A, Vps37B, Vps37C, or Vps37D), and a single auxiliary protein (ubiquitin-associated protein 1 (Ubap1) or MVB protein of 12 kDa (Mvb12A or Mvb12B)) [127,128]. The C-terminal half of Vps37, together with the N-terminal half of Vps28 and the C-terminal steadiness box (SB) domain of Vps23, are involved in the assembly of the ESCRT-I complex. The importance of Vps37 for the ESCRT-I structure and functionality is illustrated by the fact that depletion of this protein induces the destabilization of ESCRT-I and promotes strong cellular stress responses [129].

Vps37B contains the aforementioned C-terminal domain (residues 84–173) involved in the assembly of the ESCRT-I complex and a 50–170 region involved in the interaction with the ESCRT-III protein IST1 [130]. Furthermore, the regions 167–215 and 242–285 are annotated as intrinsically disordered on the corresponding UniProt page (https://www.uniprot.org/uniprotkb/Q8R0J7/entry#family_and_domains; accessed on 10 March 2024). Figure A3 provides support to this idea and shows that the C-terminal half of mouse Vps37B is predicted to be highly disordered. Based on the data reported in Figure A3A, mouse Vps37B is characterized by PPIDR values of 75.09%, 80.35%, 76.84%, 50.18%, 35.09%, and 46.67% as per the outputs of PONDR^®^ VLXT, PONDR^®^ VSL2B, PONDR^®^ VL3, PONDR^®^ FIT, IUPred_Short, and IUP_Long, respectively, and has an MPD (mean predicted disorder)-based PPIDR of 64.56%, classifying this protein as highly disordered.

Figure A3B shows that according to the results of the D^2^P^2^ analysis, disordered regions are found at residues 1–9, 13–18, 23–62, 90–102, 113–126, and 149–285 along the length of the protein. Figure A3B represents the disorder consensus bar in blue and green hues. Above this bar are conserved functional domains, modifier of rudimentary (Mod(r)) protein (residues 10–159), and endosomal sorting complex domains, ranging from 104 to 157. The protein is predicted to have six MoRFs (residues 133–144, 154–166, 188–202, 218–242, 249–263, and 279–285) and one ubiquitylation site at Lys 45.

Figure 7C,D represent the results of the FuzDrop-based analysis and show that mouse Vps37B is characterized by a high probability of spontaneous liquid–liquid phase separation, p_LLPS_ = 0.7062, implying that the protein has a high tendency to be involved in droplet formation and can act as a droplet driver. Figure A3C demonstrates the sequence distribution of residue-based, droplet-promoting probabilities and indicates that Vps37B is expected to contain two droplet-promoting regions (DPRs) positioned at residues 157–237 and 244–285. There are also five aggregation hotspots in mouse Vps37B, residues 160–168, 191–213, 218–224, 228–237, and 251–258. Figure A3D represents a multiplicity of binding modes plot and shows that there are 12 regions with context-dependent interactions in this protein, residues 4–14, 16–26, 76–82, 150–155, 160–168, 191–201, 203–213, 218–224, 228–250, 243–248, and 251–258.

The Vps37B-centered PPI network generated using STRING is shown in Figure A3E. This network includes 42 proteins involved in 636 interactions, which is significantly larger than the expected number of interactions (73), indicating that the network structure is not random as its network enrichment *p*-value is (<1.7 × 10^−16^). With an average node degree of 31.6 and average local clustering coefficient of 0.903, this PPI network is highly connected. Most enriched biological processes, molecular functions, and cellular components of the members of this network are listed in Table 2.

Figure A3F represents the model of the Vps37B 3D structure generated using AlphaFold. Although this model is characterized by an average per-residue model confidence score (p_LDDT_) of 74.5, classifying the confidence of this model as high, Figure A3F shows that the major structural element is a long, stand-alone α-helix (residues 36–100), which physically cannot exist as a stable structure and therefore potentially represents a result of a structure that can be realized in the bound state.

We also looked at the abundance of ELMs in this protein and found that Vps37B has 132 instances of 56 ELMs. Although 25 ELMs (63 instances) were filtered out by the ELM server due to the fact that they were located within a globular domain (modifier of rudimentary (Mod(r)) protein (residues 10–159)), based on the structural model shown in Figure A3F, this region in fact does not form a globular domain (see above), and therefore, all predicted ELMs should be considered here. Figure A4 represents the output of the ELM analysis and shows that the entire protein is covered by short motifs with various functions, and many ELMs are included in or overlap with the disorder-based regions discussed here: MoRFs, DPRs, aggregation hotspots, and MBP regions.

##### Appendix A.1.4. Actin Nucleation-Promoting Factor Wasl (UniProt ID: Q91YD9)

In mammals, the family of Wiskott–Aldrich syndrome protein (WASP) includes five subfamilies, such as WASP (which was the first member of the family discovered as a hematopoietically expressed protein encoded by a gene mutated in the rare X-linked immunodeficiency Wiskott–Aldrich syndrome [131]) and neuronal-WASP (N-WASP; also known as WASL), the three WASP family verprolin homolog isoforms (WAVE1–WAVE3; also known as SCAR1–SCAR3 and WASF1–WASF3), a WASP homolog associated with actin, membranes, and microtubules (WHAMM), WASP and SCAR homologs (WASH; also known as WASHC1), and a junction-mediating regulatory protein (JMY) [132,133]. Members of this family act as regulators of the generation of branched actin filaments that are involved in a multitude of biological processes, such as endocytosis and/or phagocytosis at the plasma membrane, the generation of cargo-containing vesicles from organelles including the Golgi, endoplasmic reticulum (ER), and the endo-lysosomal network, as well as formation of lamellipodia and filopodia [133]. WASP family members promote the nucleation of seven-subunit actin-related proteins-2/3 (ARP2/3) complex, acting as one of the major actin nucleators [134]. The interaction of WASP proteins with the APR2/3 complex is determined by the conserved WCA (WH2, connecting and acidic) domain [133].

WASL, also known as neural Wiskott–Aldrich syndrome protein (N-WASP), is a 501-residue-long protein with a molecular mass of 54,274 Daltons. Because of its role in actin polymerization, WASL is involved in cytokinesis and mitosis and also plays a role in the formation of cell filopodia [135]. WASL interacts with WASP activator CDC42 to form and maintain filopodia [136]. Along with cellular functions, WASL is also involved at the nuclear level, possibly playing a role in regulating gene transcription [137].

In mouse Wasl, WASP homology 1 (WH1) domain (also known as Ena/VASP Homology domain 1, EVH1) is present at residues 31–138, and the CRIB domain is located at residues 200–213. The P21-Rho-binding domain is found in the region 199–257. The WH2 motif, also named as the first tandem Wiskott–Aldrich syndrome homology region 2, is present in the region from residues 398 to 424, and the second WH2 motif is found at the position 424–449. Furthermore, Wasl contains a long proline-rich region (residues 271–391), two regions with compositional bias, a region enriched in polar residues (residues 4420459), and an acidic region (residues 482–501). PTMs are found at positions 2 (N-acetylserine), 239 (phosphoserine), 253 (phosphoserine; by FAK1 and TNK2), 304 (omega-N-methylarginine), and 481 (phosphoserine) (https://www.uniprot.org/uniprotkb/Q91YD9/entry; accessed on 10 March 2024).

Peculiarities of functional intrinsic disorder of mouse Wasl protein are shown in Figure A5. According to the multifactorial disorder analysis using RIDAO, Wasl is predicted to contain a high level of intrinsic disorder, with the C-terminal half of the protein being mostly disordered (see Figure A5A). The overall disorder content of mouse Wasl is exceeding 50%: 60.68% (PONDR^®^ VLXT), 70.46% (PONDR^®^ VSL2), 69.06% (PONDR^®^ VL3), 59.28% (PONDR^®^ FIT), 62.08% (IUPred Short), 72.46% (IUPred Long), and 64.47% (MDP).

Figure A5B represents the functional disorder profile generated using the D^2^P^2^ platform. This analysis revealed that disordered regions, where 75% of the predictors agree, are found at residues 1–7, 13–15, 135–136, 138–160, 182–199, 260–432, 434–434, 440–467, and 470–501. The ordered N-terminal region corresponds to the PH domain-like region (residues 27–138) and WHI domain (residues 28–135). Another region with somehow decreased disorder content corresponds to the P21-Rho-binding domain (PBD, residues 199–258), which is a part of the WASP C-terminal domain (residues 204–300). Finally, two WH2 motifs (residues 398–424 and 426–448), being the parts of a second WASP C-terminal domain (residues 378–489), are also expected to be more ordered than their flanking regions. Note that these two regions correspond to the characteristic dips, with mean disorder scores of 0.68 ± 0.11 and 047 ± 0.25 clearly observed in the PONDR^®^ VLXT profile (see Figure A5A). Furthermore, Wasl is predicted to contain eight MoRF regions (residues 167–178, 204–214, 219–271, 327–33, 345–352, 387–449, 458–482, and 491–501), indicating that intrinsic disorder can play an important role in the functionality of this protein. Finally, the presence of multiple different PTMs (all located within IDRs) should be emphasized (see colored circles at the bottom of Figure A5B).

Figure A5C represents a graph depicting the probability of residues to promote liquid–liquid phase separation. Droplet-promoting regions (DPRs) are found at residues 127–165, 194–222, 258–402, and 444–501. Figure A5C also shows that the aggregation hotspots are located at residues 4–9, 132–139, 155–161, 194–206, 444–454, 459–466, 470–483, and 487–492. The p_LLPS_ of 0.9796 for this protein is second highest among other proteins considered here, suggesting that the mouse Wasl protein has a very high tendency to promote the formation of membrane-less organelles and potentially acts as a droplet driver. Figure A5D shows the multiplicity of binding modes plot, illustrating that Wasl contain 13 regions with MBM values exceeding the 0.65 threshold, residues 4–23, 119–127, 132–139, 155–161, 163–170, 194–206, 220–225, 256–261, 404–471, 428–454, 459–466, 470–483, and 487–493.

The STRING analysis revealed that the mouse Wasl forms a dense PPI network that includes 232 nodes linked by 5283 edges (see Figure A5E). The number of PPIs in this network is much larger than the expected number of edges (917), indicating that this is a statistically significant PPI network with a PPI enrichment *p*-value of <1.0 × 10^−16^. The average node degree is 45.5, and the average local clustering coefficient is 0.595. Table 2 lists the most enriched biological processes, molecular functions, and cellular components of the members of this network.

Figure A5E represents the 3D structural model generated for mouse Wasl using AlphaFold and supports the idea of the high disorder content in this protein. In fact, Figure A5E shows that although Wasl is predicted to have several ordered domains and regions, it also contains multiple regions with low and very low per-residue model confidence scores (p_LDDT_), indicating that such regions can be disordered in isolation. Overall, the structural model of Wasl is characterized by an average p_LDDT_ value of 69.28, indicating that this structure is generally modeled with low confidence (70 > p_LDDT_ > 50).

At the final stage, we analyzed the presence and distribution of ELMs within the sequence of this protein. Not surprisingly, because of its length and high prevalence of disorder, mouse Wasl was predicted to have 231 instances of 65 ELMs. The results of this analysis are summarized in Figure A6 and show that many ELMs are incorporated in or overlap with the disorder-based regions discussed here: MoRFs, DPRs, aggregation hotspots, and MBP regions.
