# Supplementary material for: Intrinsic Disorder in the Host Proteins Entrapped in Rabies Virus Particles
Source: Viruses. 2024 Jun 4;16(6):916. doi: 10.3390/v16060916 (PMC11209445; doi:10.3390/v16060916)
Supplement: Supplementary file 1 [file viruses-16-00916-s001.zip › viruses-2950452-suppl-conv.pdf]

Supplementary Materials S1. Brief description of the protocol utilized by Zhang and colleagues for the analysis of the host proteins entrapped within the RABV nanoparticles.

The authors used nano-scale liquid chromatography tandem mass spectrometry techniques on purified viral particles to identify 49 virus-associated host proteins [16]. Then, the Western blotting approach was used to validate the presence of these proteins in the matured viral particles [16]. They used RABV, CVS-11 strain to infect mouse Neuro-2A cells [16]. This step was crucial to obtain virus particles for further analysis [16]. In these experiments, cells were infected at 70% confluence, and the virus-containing supernatant was subject to multiple rounds of differential centrifugation [16]. Ultra-centrifugation at 100,000× *g* was performed on samples containing bullet-shaped viruses for higher purification [16]. The purified viral particles isolated through centrifugal separation were observed through transmission electron microscopy and cryo-electron microscopy to inspect and verify the purity and structural integrity of viral particles [16].

At the next stage, the deglycosylation of proteins by the PNGase F enzyme was conducted to enhance the sensitivity to the peptides that contain glycosylation sites [16,20,21]. The proteins were analyzed through SDS-PAGE to separate them based on their molecular weight [16], followed by Western blotting using a monoclonal antibody against the RABV glycoprotein and a secondary antibody labeled with Alexa Flours 680 for detection [16].

Next, the proteomic analysis was conducted by nano LC-MS/MS on the peptide mixtures obtained through the digestion of deglycosylated protein samples with trypsin [16]. Analysis of proteins through Liquid Chromatography-Tandem Mass Spectrometry allowed for the detailed analysis of peptides, including their structure and sequence [16]. To ensure accuracy and reliability, the data obtained through mass spectrometry were then analyzed using the Andromeda search engine and MaxQuant software against specific databases, such as UniProtKB mouse sequence database and RABV (CVS-11) protein sequences from GenBank [16]. The authors employed intensity-based absolute quantification (iBAQ) for evaluation of the protein abundance and applied FDR (False Discovery Rate) of 0.01 % to ensure the data accuracy [16]. To further increase the confidence, Zhang et al. did not rely solely on one experimental run but used three different assays [16].

Then, to investigate the incorporation of host proteins into the virus particles, purified virus particles were treated with protease K to digest proteins, which helps in focusing on proteins that are truly incorporated into the viral particles and in removing the loosely attached proteins [16]. The treated virions were processed to remove any cleaved peptides [16].

To prepare the cell extracts, 2 groups of N2a cell lines were prepared; one treated with CVS11 and the other serves as control. The supernatant of the cell extracts was collected after 72 hours, these liquid parts contain the proteins released from the cells [16]. Both the cell extracts and virus particles the one treated with protease K and untreated were subjected to Western blotting. The goal was to identify the proteins in Virus particles and cell extracts using antibodies [16].

Viral proteins, such as Glycoprotein G, Nucleoprotein N, and matrix protein M were detected using specific mouse polyclonal antibodies, while host cell proteins, such as Hsc70, cofilin, and Chmp4b were probed through additional antibodies [16]. Finally, all these proteins were visualized using the fluorescently labeled secondary antibodies [16]. The overall step was crucial to check whether host proteins were incorporated into the virus particles and do they incorporate in them firmly [16].

File S1. Amino acid sequences of proteins analyzed in this study.

>sp|P06837|NEUM-MOUSE Neuromodulin OS=Mus musculus OX=10090 GN=Gap43 PE=1 SV=1
MLCCMRRTKQVEKNDEDQKIEQDGVKPEDKAHKAATKIQASFRGHITRKKLKGEKKG-
DAPAAEAEAKEKDDAPVADGVEKKEGDGSATTDAAPATSPKAEEPSKAGDAPSEEKKGEGDAAPSEEKAGSAETESAAKATTDNSPSSKAEDGPAKEEPKQADVPAAVTDAAATTPAAEDAATKAAQPPTETAES
SQAEEEKDAVDEAKPKESARQDEGKEDPEADQEHA

>sp|O54946|DNJB6-MOUSE DnaJ homolog subfamily B member 6 OS=Mus musculus OX=10090 GN=Dnajb6 PE=1 SV=4
MVDYYEVLGVQRHASPEDIKKAYRKQALKWHPDKNPENKEEAERK-
FKQVAEAYEVLSDAKKRDIYDKYGKEGLNGGGGGGGIHFDSPFEFGFTFRNPDDVFREFFGGRDPFSFDFFEDPFDDFFGNRRGPRGNRSRGAGSFFSTFSGFPSFGSGFPAFDTGFTPFGSLGHGGLTSFSSTSFGGSGM
GNFKSISTSTKIVNGKKITTKRIVENGQERVEVEEDGQLKSLTINGVADENALAEEC-
QRRGQPTPALAPGPAPAPVRVPSQARPLAPTPAPTPAPTPAPAPAQTPAPSVSTRPQKPPRPAPTAKLGSKSNWEDDEQDRQRVPGNWDAPMTSAGLKEGGKRKKQKQKEDLKKKKSTKGNH

>sp|Q9DB34|CHM2A-MOUSE Charged multivesicular body protein 2a OS=Mus musculus OX=10090 GN=Chmp2a PE=1 SV=1
MDLLFGRRKTPEELLRQNQRALNRAMRELDRERQKLETQEKKIIADIKKMAKQGQMDAV-
RIMAKDLVRTRRYVRKFVLMRANIQAVSLKIQTLKSNNSMAQAMKGVTKAMGTMNRQLKLPQIQKIMMEFERQAEIMDMKEEMMNDAIDDAMGDEEDEEESDAVVSQVLDELGLSLTDELSNLPSTGGSLSVAA
GGKKAEATASALADADADLEERLKNLRRD

>sp|Q8R0J7|VP37B-MOUSE Vacuolar protein sorting-associated protein 37B OS=Mus musculus OX=10090 GN=Vps37b PE=1 SV=1
MAGAVSEARFAGLSLMQLHELLEDDAQLGDMVRG-
MEEAQTVQLNKEMTLASNRSLAEGNLLYQPQLDAQKARLTQKYQELQVLFEAYQIKKTKLDKQSNNASLETLLALLQAEGAKIEEDTENMAEKFLDGELPLDSFIDVYQSKRKLAHMRRVKVEKLQELVLKGQRH
PQAGAPPPPRVPEPSPATALPYPSLEATGLPSVVPRRIPPPPPPVPAGHVATPFAAAMGSGQVSAYPGLQCPPLPPRVGLPSQQGFSAQLVSPYPPALPQRPPPRMAPHQPGFILQ

>sp|Q91YD9|WASL-MOUSE Actin nucleation-promoting factor WASL OS=Mus musculus OX=10090 GN=Wasl PE=1 SV=1
MSSGQQP-
PRRVTNVGSLLLTPQENESLFSFLGKKCVTMSSAVVQLYAADRNCMWAKKCSGVACLVKDNPQRSYFLRIFDIKDGKLLWEQELYNNFVYNSPRGYFHTFAGDTCQVALNFANEEEAKKFRKAVTDLLGRRQRKSE
KRRDAPNGPNLPMATVDIKNPEITTNRFYGSQVNNISHTKEKKKGKAKKKRLTKADIGTP-
SNFQHIGHVGWDPNTGFDLNNLDPELKNLFDMCGISEAQLKDRETSKVIYDFIEKTGGVEAVKNELRRQAPPPPPPSRGGPPPPPPPPHSSGPPPPPARGRGAPPPPPSRAPTAAPPPPPPSRPGVVVPPPPPNRMYPPP
PPALPSSAPSGPPPPPPPSMAG-
STAPPPPPPPPPPPPGPPPPPGLPSDGDHQVPAPSGNKAALLDQIREGAQLKKVEQNSRPVSCSGRDALLDQIRQGIQLKSVSDGQESTPPTPAPTSGIVGALMEVMQKRSKAIHSSDEDEDDDDEEDFEDDDEWED

>sp|P63024|VAMP3-MOUSE Vesicle-associated membrane protein 3 OS=Mus musculus OX=10090 GN=Vamp3 PE=1 SV=1
MSTGVPSGSSAATGSNRRLQQTQNQVDEVVDIMRVNVDKVLERDQKLSELDDRADALQAGASQFETSAAKLKRKYWWKNCKMWAIGISVLVIIVIIIIVWCVS

>sp|Q9WVE8|PACN2-MOUSE Protein kinase C and casein kinase substrate in neurons protein 2 OS=Mus musculus OX=10090 GN=Pacsin2 PE=1 SV=1
MSVTYDDSVGVEVSSDSFWEVGNYKRTVKRIDDGHRLCGDLMNCLHERARIEKAYA-
QQLTEWARRWRQLVEKGPQYGTVEKAWIAVMSEAERVSELHLEVKASLMNEDFEKIKNWQKEAFHKQMMGGFKETKEAEDGFRKAQKPWAKKLKEVEAAKKAHHTACKEEKLAISREANSKADPSLNPEQLK

KLQDKIEKCKQDVLKTKDKYEKSLKELDQTTPQYMEN-
MEQVFEQCQQFEEKRLRFFREVLLEVQKHLDLSNVASYKTIYRELEQSIKAADAVEDLRWFRANHGPGMAMNWPQFEEWSADLNRTLSRREKKKAVDGVTLTGINQTGDQSGQNKPGSNLSVPSNPAQSTQLQSS
YNPFEDEDDTGSSISEKEDIKAKNVSSYEKTQTYPTDWSDDESNNPFSSTDANGDSNPFDEDTTSGTEVRVRALYDYEGQEHDELSFKAGDELTKIEDEDEQGWCKGRLDSGQVGLYPANYVEAIQ

>sp|P63037|DNJA1-MOUSE DnaJ homolog subfamily A member 1 OS=Mus musculus OX=10090 GN=Dnaja1 PE=1 SV=1
MVKETTYYDVLGVKPNATQEELKKAYRKLALKYHPDKNPNEGEKFKQISQAYEVLADSKKRE-
LYDKGGEQAIKEGGAGGGFGSPMDIFDMFFGGGGRMQRERRGKNVVHQLSVTLEDLYNGATRKLALQKNVICDKCEGRGGKKGAVECCPNCRGTGMQIRIHQIGPGMVQQIQSVCMECQGHGERISPKDRCKSC
NGRKIVREKKILEVHIDKGMKDGQKITFHGEGDQEPGLEPGDIIIVLDQKDHA-
VFTRRGEDLFMCMDIQLVEALCGFQKPISTLDNRTIVITSHPGQIVKHGDIKCVLNEGMPIYRRPYEKGRLIIEFKVNFPENGFLSPDKLSLLEKLLPERKEVEETDEMDQVELVDFDPNQERRRHYNGEAYEDDEHHPR
GGVQCQTS

>sp|P26040|EZRI-MOUSE Ezrin OS=Mus musculus OX=10090 GN=Ezr PE=1 SV=3
MPKPINVRVTTMDAELEFAIQPNTTGKQLFDQVVKTIGLREVWYFGLQYVDNKGFPT-
WLKLDKKVSAQEVRKENPVQFKFRAKFYPEDVAEELIQDITQKLFFLQVKDGILSDEIYCPPETAVLLGSYAVQAKFGDYNKEMHKSGYLSSERLIPQRVMDQHKLSRDQWEDRIQVWHAEHRGMLKDSAMLEYLKI
AQDLEMYGINYFEIKNKKGTDLWLGVDALGLNIYEKDDKLTPKIGFPWSEIRNISFND-
KKFVIKPIDKKAPDFVFYAPRLRINKRILQLCMGNHELYMRRRKPDTIEVQQMKAQAREEKHQKQLERQQLETEKKRRETVEREKEQMLREKEELMLRLQDYEQKTKRAEKELSEQIEKALQLEEERRRAQEEAERLE
ADRMAALRAKEELERQAQDQIKSQEQLAAELAEYTAKIALLEEARRRKEDEV-
EEWQHRAKEAQDDLVKTKEELHLVMTAPPPPPPPVYEPVNYHVQEGLQDEGAEPMGYSAELSSEGILDDRNEEKRITEAEKNERVQRQLLTLSNELSQARDENKRTHNDIIHNENMRQGRDKYKTLRQIRQGNTK
QRIDEFEAM

>sp|P63101|1433Z-MOUSE 14-3-3 protein zeta/delta OS=Mus musculus OX=10090 GN=Ywhaz PE=1 SV=1
MDKNELVQKAKLAEQAERYDDMAACMKSVTE-
QGAELSNEERNLLSVAYKNVVGARRSSWRVVSSIEQKTEGAEKKQQMAREYREKIETELRDICNDVLSLLEKFLIPNASQPESKVFYLKMKGDYYRYLAEVAAGDDKKGIVDQSQQAYQEAFEISKKEMQPTHPIRLG
LALNFSVFYYEILNSPEKACSLAKTAFDEAIAELDTLSEESYKDSTLIMQLLRDNLTLWTSDTQGDEAEAGEGGEN

>sp|Q61187|TS101-MOUSE Tumor susceptibility gene 101 protein OS=Mus musculus OX=10090 GN=Tsg101 PE=1 SV=2
MAVSESQLKKMMSKYKYRDLTVRQTVNVIAMYKDLKPVLDSYV-
FNDGSSRELVNLTGTIPVRYRGNIYNIPICLWLLDTYPYNPPICFVKPTSSMTIKTGKHVDANGKIYLPYLHDWKHPRSELLELIQIMIVIFGEEPPVFSRPTVSASYPPYTATGPPNTSYMPGMPSGISAYPSGYPPNPSGYP
GCPYPPAGPYPATTSSQYPSQPPVTTVGPSRDGTISEDTIRASLISAVSDKLR-
WRMKEEMDGAQAELNALKRTEEDLKKGHQKLEEMVTRLDQEVAEVDKNIELLKKKDEELSSALEKMENQSENNDIDEVIIPTAPLYKQILNLYAEENAIEDTIFYLGEALRRGVIDLDVFLKHVRLLSRKQFQLRAL
MQKARKTAGLSDLY

>sp|P11499|HS90B-MOUSE Heat shock protein HSP 90-beta OS=Mus musculus OX=10090 GN=Hsp90ab1 PE=1 SV=3
MPEEVHHGEEEVETFAFQAEIAQLMSLIINTFYSNKEIFLRELISNASDALDKIRYESLT-
DPSKLDSGKELKIDIIPNPQERTLTLVDTGIGMTKADLINNLGTIAKSGTKAFMEALQAGADISMIGQFGVGFYSAYLVAEKVVVITKHNDDEQYAWESSAGGSFTVRADHGEPIGRGTKVILHLKEDQTEYLEERRVK
EVVKKHSQFIGYPITLYLEKEREKEISDDEAEEEKGEKEEEDKEDEEK-
PKIEDVGSDEEDDSGKDKKKKTKKIKEKYIDQEELNKTKPIWTRNPDDITQEEYGEFYKSLTNDWEDHLAVKHFSVEGQLEFRALLFIPRRAPFDLFENKKKKNNIKLYVRRVFIMDSCDELIPEYLNFIRGVVDSEDLPL

NISREMLQQSKILKVIRKNIVKKCLELFSELAEDKENYKKFYEAFSKNLKLGI-
HEDSTNRRRLSELLRYHTSQSGDEMTSLSEYVSRMKETQKSIYYITGESKEQVANSAFVERVRKRGFEVVYMTEPIDEYCVQQLKEFDGKSLVSVTKEGLELPEDEEEKKKMEESKAKFENLCKLMKEILDKKVEKVTIS
NRLVSSPCCIVTSTYGWTANMERIMKAQALRD-
NSTMGYMMAKKHLEINPDHPIVETLRQKAEADKNDKAVKDLVVLLFETALLSSGFSLEDPQTHSNRIYRMIKLGLGIDEDEVTAEEPSAAVPDEIPPLEGDEDASRMEEVD

>sp|Q91ZR2|SNX18-MOUSE Sorting nexin-18 OS=Mus musculus OX=10090 GN=Snx18 PE=1 SV=1
MALRARALYDFKSENPGEISLREHEVLSLCSEQDIEGWLEGINSRGDRGLFPASYV-
QVIRAPEPGPPADGGPGAPARYANVPPGGFEPLPAAPPAAFPPLLQPQASPGSFQPPGAGFPYGGGALQPSPQQLYGGYQASLGSDDDWDDEWDDSSTVADEPGALGSGAYPDLDGSSSAGGGAAGRYRLSTRSDL
SLGSRGVSAPPAPSVWSQELGHGEPQPQSLLHLRQVGRGGLRAGRGVRLREGWGQAVRG-
AGSYGPEWQENPYPFQCTIDDPTKQTKFKGMKSYISYKLVPTHTQVPVHRRYKHFDWLYARLAEKFPVISVPHLPEKQATGRFEEDFISKRRKGLIWWMNHMASHPVLAQCDVFQHFLTCPSSTDEKAWKQGKRKA
EKDEMVGANFFLTLSTPPAAALDLQEVESKIDGFKCFTKKMDDSALQLNHTANE-
FARKQVTGFKKEYQKVGQSFRGLSQAFELDQQAFSVGLNQAIAFTGDAYDAIGELFAEQPRQDLDPVMDLLALYQGHLANFPDIIHVQKGALTKVKESRRHVEEGKMEVQKADGIQDRCNTISFATLAEIHHFHQI
RVRDFKSQMQHFLQQQIIFFQKVTQKLEEALHKYDSV

>sp|Q9WU78|PDC6I-MOUSE Programmed cell death 6-interacting protein OS=Mus musculus OX=10090 GN=Pdcd6ip PE=1 SV=3
MASFIWVQLKKTSEVDLAKPLVKFIQQTYPSGGEEQAQYCRAAEELSKLRRSALGRPLDKHE-
GALETLLRYYDQICSIEPKFPFSENQICLTFTWKDAFDKGSLFGGSVKLALASLGYEKSCVLFNCAALASQIAAEQNLDNDEGLKTAAKQYQFASGAFLHIKDTVLSALSREPTVDISPDTVGTLSLIMLAQAQEVFFLK
ATRDKMKDAIIAKLANQAADYFGDAFKQCQYKDTLPKEVFPT-
LAAKQCIMQANAEYHQSILAKQQKKFGEEIARLQHAAELIKNVASRYDEYVNVKDFSDKINRALTAAKKDNDFIYHDRVPDLKDLDPIGKATLVKPTPVNVPVSQKFTDLFEKMVPVSVQQSLAVFSQRKADLVNR
SIAQMREATTLANGVLASLNLPAAIEDVSGDTVPQSILTKSTSVVEQG-
GIQTVDQLIKELPELLQRNREILEESLRLLDEEEATDNDLRAKFKDRWQRTPSNDLYKPLRAEGAKFRAVLDKAVQADGQVKERYQSHRDTIALLCKPEPELNAAIPSANPAKTMQGSEVVSVLKSLLSNLDEIKKERE
SLENDLKSVNFDMTSKFLTALAQDGVINEEALSVTELDRIYGGLTSKVQESLKKQEGLLK-
NIQVSHQEFSKMKQSNNEANLREEVLKNLATAYDNFVELVANLKEGTKFYNELTEILVRFQNKCSDIVFARKTERDELLKDLQQSIAREPSAPSIPPPAYQSSPAAGHAAAPPTPAPRTMPPAKPQPPARPPPPVLPAN
RVPPASAAAAPAGVGTASAAPPQTPGSAPPPQAQGPPYPTYPGYPGYCQMPMPMGYNPYAYGQYNMPYPPVYHQSPGQAPYPGPQQPTYPFPQPPQQSYYPQQ

>sp|Q62167|DDX3X-MOUSE ATP-dependent RNA helicase DDX3X OS=Mus musculus OX=10090 GN=Ddx3x PE=1 SV=3
MSHVAVENALGLDQQFAGLDLNSSDNQSGGSTASKGRYIPPHLRNREATKGFYDKDSSGWS-
SSKDKDAYSSFGSRGDSRGKSSFFGDRGSGSRGRFDDRGRGDYDGIGGRGDRSGFGKFERGGNSRWCDKSDEDDWSKPLPPSERLEQELFSGGNTGINFEKYDDIPVEATGNNCPPHIESFSDVEMGEIIMGNIELTRYT
RPTPVQKHAIPIIKEKRDLMACAQTGSGKTAAFLLPILSQIYADGPGEALRAMKENGRYGRR-
KQYPISLVLAPTRELAVQIYEEARKFSYRSRVRPCVVYGGAEIGQQIRDLERGCHLLVATPGRLVDMMERGKIGLDFCKYLVLDEADRMLDMGFEPQIRRIVEQDTMPPKGVRHTMMFSATFPKEIQMLARDFLDEYIF
LAVGRVGSTSENITQKVVWVEEIDKRSFLLDLLNATGKDSLTLVFVETKKGADSLED-
FLYHEGYACTSIHGDRSQRDREEALHQFRSGKSPILVATAVAARGLDISNVKHVINFDLPSDIEEYVHRIGRTGRVGNLGLATSFFNERNINITKDLLDLLVEAKQEVPSWLENMAFEHHYKGSSRGRSKSSRFSGGFGA
RDYRQSSGASSSSFSSSRASSSRSGGGGHGGSRGFGGGGYGGFYNSDGYGGNYNSQGVDWWGN

>tr|Q4VAE6|Q4VAE6-MOUSE Ras family member A OS=Mus musculus OX=10090 GN=Rhoa PE=1 SV=1
MAAIRKKLVIVGDGACGKTCLLIVFSKDQFPEVYVPTVFENYVADIEVDGKQVELALWD-
TAGQEDYDRLRPLSYPDTDVILMCFSIDSPDSLENIPEKWTPEVKHFCPNVPIILVGNKKDLRNDEHTRRELAKMKQEPVKPEEGRDMANRIGAFGYMECSAKTKDGVREVFEMATRAALQARRGKKKSGCLIL

>sp|P63017|HSP7C-MOUSE Heat shock cognate 71 kDa protein OS=Mus musculus OX=10090 GN=Hspa8 PE=1 SV=1
MSKGPAVGIDLGTTYSCVGVFQHGKVEIIANDQGNRTTPSYVAFTDTERLIGDAAK-
NQVAMNPTNTVFDAKRLIGRRFDDAVVQSDMKHWPFMVVNDAGRPKVQVEYKGETKSFYPEEVSSMVLTKMKEIAEAYLGKTVTNAVVTVPAYFNDSQRQATKDAGTIAGLNVLRIINEPTAAAIAYGLDKKVG
AERNVLIFDLGGGTFDVSILTIEDGIFEVKSTAGDTHLGGEDFDNRMVNH-
FIAEFKRKHKKDISENKRAVRRLRTACERAKRTLSSSTQASIEIDSLYEGIDFYTSITRARFEELNADLFRGTLDPVEKALRDAKLDKSQIHDIVLVGGSTRIPKIQKLLQDFFNGKELNKSINPDEAVAYGAAVQAAILSG
DKSENVQDLLLLDVTPLSLGIETAGGVMTVLIKRNTTIPTKQTQTFTTYS-
DNQPGVLIQVYEGERAMTKDNNLLGKFELTGIPPAPRGVPQIEVTFDIDANGILNVSAVDKSTGKENKITITNDKGRLSKEDIERMVQEAEKYKAEDEKQRDKVSSKNSLESYAFNMKATVEDEKLQGKINDEDKQKI
LDKCNEIISWLDKNQTAEKEEFEHQQKELEKVCNPIITKLYQSAGGMPGGMPGGFPGGGAPPSGGASSGPTIEEVD

>sp|Q9D1C8|VPS28-MOUSE Vacuolar protein sorting-associated protein 28 homolog OS=Mus musculus OX=10090 GN=Vps28 PE=1 SV=1
MFHGIPATPGVGAPGNKPELYEEVKLYKNAREREKYDNMAELFAVVKTMQALEKAYIK-
DCVTPNEYTAACSRLLVQYKAAFRQVQGSEISSIDEFCRKFRLDCPLAMERIKEDRPITIKDDKGNLNRCIADVVSLFITVMDKLRLEIRAMDEIQPDLRELMETMHRMSHLPPDFEGRQTVSQWLQTLSGMSASDELD
DSQVRQMLFDLESAYNAFNRFLHA

>sp|P63168|DYL1-MOUSE Dynein light chain 1, cytoplasmic OS=Mus musculus OX=10090 GN=Dynll1 PE=1 SV=1
MCDRKAVIKNADMSEEMQQDSVECATQALEKYNIEKDIAAHIKKEFDKKYNPTWHCIVGRNFGSYVTHETKHFIYFYLGQVAILLFKSG

>sp|P10852|4F2-MOUSE 4F2 cell-surface antigen heavy chain OS=Mus musculus OX=10090 GN=Slc3a2 PE=1 SV=1
MSQDTEVDMKDVELNELEPEKQPMNAADGAAAGEKNGLVKIKVAEDETEAGVKFTGLSKEEL-
LKVAGSPGWVRTRWALLLLFWLGWLGMLAGAVVIIVRAPRCRELPVQRWWHKGALYRIGDLQAFVGRDAGGIAGLKSHLEYLSTLKVKGLVLGPIHKNQKDEINETDLKQINPTLGSQEDFKDLLQSAKKKSIHIIL
DLTPNYQGQNAWFLPAQADIVATKMKEALSSWLQDGVDGFQFRDVGKLMNAP-
LYLAEWQNITKNLSEDRLLIAGTESSDLQQIVNILESTSDLLLTSSYLSNSTFTGERTESLVTRFLNATGSQWCSWSVSQAGLLADFIPDHLLRLYQLLLFTLPGTPVFSYGDELGLQGALPGQPAKAPLMPWNESSIFHIP
RPVSLNMTVKGQNEDPGSLLTQFRRLSDLRGKERSLLHGDFHALSSSPDLFSYIRHWDQNERYLVVLNFRDSGRSARLGASNLPAGISLPASAKLLLSTDSARQSREEDTSLKLENLSLNPYEGLLLQFPFVA

>sp|P60335|PCBP1-MOUSE Poly(rC)-binding protein 1 OS=Mus musculus OX=10090 GN=Pcbp1 PE=1 SV=1
MDAGVTESGLNVTLTIRLLMHGKEVGSIIGKKGESVKRIREESGARINISEGNCPE-
RIITLTGPTNAIFKAFAMIIDKLEEDINSSMTNSTAASRPPVTLRLVVPATQCGSLIGKGGCKIKEIRESTGAQVQVAGDMLPNSTERAITIAGVPQSVTECVKQICLVMLETLSQSPQGRVMTIPYQPMPASSPVICAGGQ
DRCSDAAGYPHATHDLEGPPLDAYSIQGQHTISPLDLAKLNQVARQQSHFAMMHGGTG-
FAGIDSSSPEVKGYWASLDASTQTTHELTIPNNLIGCIIGRQGANINEIRQMSGAQIKIANPVEGSSGRQVTITGSAASISLAQYLINARLSSEKGMGCS

>sp|P46467|VPS4B-MOUSE Vacuolar protein sorting-associated protein 4B OS=Mus musculus OX=10090 GN=Vps4b PE=1 SV=2
MASTNT-
NLQKAIDLASKAAQEDKAGNYEEALQLYQHAVQYFLHVVKYEAQGDKAKQSIRAKCTEYLDRAEKLKEYLKKKEKKPQKPVKEEQSGPVDEKGNDSDGEAESDDPEKKKLQNQLQGAIVIERPNVKWSDVAGLE
GAKEALKEAVILPIKFPHLFTGKRTPWRGILLFGPPGTGKSYLAKAVATEANNSTFFSIS-
SSDLVSKWLGESEKLVKNLFQLARENKPSIIFIDEIDSLCGSRSENESEAARRIKTEFLVQMQGVGVDNDGILVLGATNIPWVLDSAIRRRFEKRIYIPLPEAHARAAMFRLHLGSTQNSLTEADFQELGRKTDGYSGADIS
IIVRDALMQPVRKVQSATHFKKVRGPSRADPNCIVNDLLTPCSPGDPGAIEMTWMDVPGDKLLEPVVSMWDMLRSLSSTKPTVNEQDLLKLKKFTEDFGQEG

>sp|Q99J93|IFM2-MOUSE Interferon-induced transmembrane protein 2 OS=Mus musculus OX=10090 GN=Ifitm2 PE=1 SV=1

MSHNSQAFLSTNAGLPPSYETIKEEYGVTELGEPSNSAVVRT-
TVINMPREVSVPDHVVWSLFNTLFFNACCLGFVAYAYSVKSRDRKMVGDVVGAQAYASTAKCLNISSLIFSILMVIICIIIFSTTSVVVFQSFAQRTPHSGF

>sp|P63242|IF5A1-MOUSE Eukaryotic translation initiation factor 5A-1 OS=Mus musculus OX=10090 GN=Eif5a PE=1 SV=2
MADDLDFETGDAGASATFPMQCSALRKNGFVVLKGRPCKIVEMSTSKTGKHGHAK-
VHLVGIDIFTGKKYEDICPSTHNMDVPNIKRNDFQLIGIQDGYLSLLQDSGEVREDLRLPEGDLGKEIEQKYDCGEEILITVLSAMTEEAAVAIKAMAK

>sp|P18760|COF1-MOUSE Cofilin-1 OS=Mus musculus OX=10090 GN=Cfl1 PE=1 SV=3
MASGVAVSDGVIKVFNDMKVRKSSTPEEVKKRKKAVLFCLSEDKKNIILEE-
GKEILVGDVGQTVDDPYTTFVKMLPDKDCRYALYDATYETKESKKEDLVFIFWAPENAPLKSKMIYASSKDAIKKKLTGIKHELQANCYEEVKDRCTLAEKLGGSAVISLEGKPL

>sp|P0CG50|UBC-MOUSE Polyubiquitin-C OS=Mus musculus OX=10090 GN=Ubc PE=1 SV=2
MQIFVKTLTGKTITLEVEPSDTIENVKAKIQDKEGIPPDQQRLI-
FAGKQLEDGRTLSDYNIQKESTLHLVLRLRGGMQIFVKTLTGKTITLEVEPSDTIENVKAKIQDKEGIPPDQQRLIFAGKQLEDGRTLSDYNIQKESTLHLVLRLRGGMQIFVKTLTGKTITLEVEPSDTIENVKAKIQDKE
GIPPDQQRLIFAGKQLEDGRTLSDYNIQKESTLHLVLRLRGGMQIFVKTLTGK-
TITLEVEPSDTIENVKAKIQDKEGIPPDQQRLIFAGKQLEDGRTLSDYNIQKESTLHLVLRLRGGMQIFVKTLTGKTITLEVEPSDTIENVKAKIQDKEGIPPDQQRLIFAGKQLEDGRTLSDYNIQKESTLHLVLRLRGGM
QIFVKTLTGKTITLEVEPSDTIENVKAKIQDKEGIPPDQQRLI-
FAGKQLEDGRTLSDYNIQKESTLHLVLRLRGGMQIFVKTLTGKTITLEVEPSDTIENVKAKIQDKEGIPPDQQRLIFAGKQLEDGRTLSDYNIQKESTLHLVLRLRGGMQIFVKTLTGKTITLEVEPSDTIENVKAKIQDKE
GIPPDQQRLIFAGKQLEDGRTLSDYNIQKESTLHLVLRLRGGMQIFVKTLTGK-
TITLEVEPSDTIENVKAKIQDKEGIPPDQQRLIFAGKQLEDGRTLSDYNIQKESTLHLVLRLRGGMQIFVKTLTGKTITLDVEPSVTTKKVKQEDRRTFLTTVSKKSPPCACSWV

>sp|O35566|CD151-MOUSE CD151 antigen OS=Mus musculus OX=10090 GN=Cd151 PE=1 SV=2
MGEFNEKKATCGTVCLKYLLFTYNCCFWLAGLAVMAVGIWTLALKSDYISLLASSTYLATA-
YILVVAGVVVMVTGVLGCCATFKERRNLLRLYFILLLIIFLLEIIAGILAYVYYQQLNTELKENLKDTMVKRYHQSGHEGVSSAVDKLQQEFHCCGSNNSQDWQDSEWIRSGEADSRVVPDSCCKTMVAGCGKRDHA
SNIYKVEGGCITKLETFIQEHLRVIGAVGIGIACVQVFGMIFTCCLYRSLKLEHY

>sp|P17742|PPIA-MOUSE Peptidyl-prolyl cis-trans isomerase A OS=Mus musculus OX=10090 GN=Ppia PE=1 SV=2
MVNPTVFFDITADDEPLGRVSFELFADKVPKTAENFRALSTGEKGFGYKGSSFHRIIPGFMC-
QGGDFTRHNGTGGRSIYGEKFEDENFILKHTGPGILSMANAGPNTNGSQFFICTAKTEWLDGKHVVFGKVKEGMNIVEAMERFGSRNGKTSKKITISDCGQL

>sp|Q9R0P5|DEST-MOUSE Destrin OS=Mus musculus OX=10090 GN=Dstn PE=1 SV=3
MASGVQVADEVCRIFYDMKVRKCSTPEEIKKRKKAVIFCLSADKKCIVVEEGKEILVGDVGA-
TITDPFKHFVGMLPEKDCRYALYDASFETKESRKEELMFFLWAPEQAPLKSKMIYASSKDAIKKKFPGIKHEYQANGPEDLNRTCIAEKLGGSLIVAFEGSPV

>sp|P35278|RAB5C-MOUSE Ras-related protein Rab-5C OS=Mus musculus OX=10090 GN=Rab5c PE=1 SV=2
MAGRGGAARPNGPAAGNKICQFKLVLLGESAVGKSSLVLRFVKGQFHEYQESTI-
GAAFLTQTVCLDDTTVKFEIWDTAGQERYHSLAPMYYRGAQAAIVVYDITNTDTFARAKNWVKELQRQASPNIVIALAGNKADLASKRAVEFQEAQAYADDNSLLFMETSAKTAMNVNEIFMAIAKKLPKNEPQ
NAAGAPGRTRGVDLQESNPASRSQCCSN

>tr|Q3UFR4|Q3UFR4-MOUSE Amino acid transporter OS=Mus musculus OX=10090 GN=Slc1a5 PE=2 SV=1
MAVDPPKADPKGVVAVDSTANGGPALGSREDQSAKAGGCCGSRDRVRRCIRANLLVLL-
TVAAVVAGVGLGLGVSAAGGADALGPARLTAFAFPGELLLRLLKMIILPLVVCSLIGGAASLDPSALGRVGAWALLFFLVTTLLASALGVGLALALKPGAAVTAITSINDSVVDPCARSAPTKEVLDSFLDLVRNIFPS
NLVSAAFRSFATSYEPKDNSCKIPQSCIQREINSTMVQLLCEVEGMNILGLVVFAIVFGVAL-
RKLGPEGELLIRFFNSFNDATMVLVSWIMWYAPVGILFLVASKIVEMKDVRQLFISLGKYILCCLLGHAIHGLLVLPLIYFLFTRKNPYRFLWGIMTPLATAFGTSSSSATLPLMMKCVEEKNGVAKHISRFILPIGATVN
MGGAALFQCVAAVFIAQLNGVSLDFVKIITILVTATASS-
VGAAGIPAGGVLTLAIILEAVSLPVKDISLILAVDWLVDRSCTVLNVEGDAFGAGLLQSYVDRTKMPSSEPELIQVKNEVSLNPLPLATEEGNPLLKQYQGPTGDSSATFEKESVM

>sp|P99024|TBB5-MOUSE Tubulin beta-5 chain OS=Mus musculus OX=10090 GN=Tubb5 PE=1 SV=1
MREIVHIQAGQCGNQIGAKFWEVISDEHGIDPTGTYH-
GDSDLQLDRISVYYNEATGGKYVPRAILVDLEPGTMDSVRSGPFGQIFRPDNFVFGQSGAGNNWAKGHYTEGAELVDSVLDVVRKEAESCDCLQGFQLTHSLGGGTGSGMGTLLISKIREEYPDRIMNTFSVVPSPKV
SDTVVEPYNATLSVHQLVENTDETYCIDNEALYDICFRTLKLTTPTYG-
DLNHLVSATMSGVTTCLRFPGQLNADLRKLAVNMVPFPRLHFFMPGFAPLTSRGSQQYRALTVPELTQQVFDAKNMMAACDPRHGRYLTVAAVFRGRMSMKEVDEQMLNVQNKNSSYFVEWIPNNVKTAVCDI
PPRGLKMAVTFIGNSTAIQELFKRISEQFTAMFRRKAFLHWYTGEGMDEMEFTEAESNMNDLVSEYQQYQDATAEEEEDFGEEAEEEA

>sp|P51150|RAB7A-MOUSE Ras-related protein Rab-7a OS=Mus musculus OX=10090 GN=Rab7a PE=1 SV=2
MTSRKKVLLKVIILGDSGVGKTSLMNQYVNKKFSNQYKATIGADFLTKEVMVDDRLVTM-
QIWDTAGQERFQSLGVAFYRGADCCVLVFDVTAPNTFKTLDSWRDEFLIQASPRDPENFPFVVLGNKIDLENRQVATKRAQAWCYSKNNIPYFETSAKEAINVEQAFQTIARNALKQETEVELYNEFPEPIKLDKNDR
AKASAESCSC

>sp|O08992|SDCB1-MOUSE Syntenin-1 OS=Mus musculus OX=10090 GN=Sdcbp PE=1 SV=1
MSLYPSLEDLKVDKVIQAQTAYSANPASQAFVLVDASAALPPDGNLYPKLYPELSQYMGL-
SLNEAEICESMPMVSGAPAQGQLVARPSSVNYMVAPVTGNDAGIRRAEIKQGIREVILCKDQDGKIGLRLKSIDNGIFVQLVQANSPASLVGLRFGDQVLQINGENCAGWSSDKAHKVLKQAFGEKITMTIRDRPFER
TVTMHKDSSGHVGFIFKSGKITSIVKDSSAARNGLLTDHHICEINGQNVIGLKDAQIADILSTAGTVVTITIMPTFIFEHIIKRMAPSIMKSLMDHTIPEV

>tr|B2RRX1|B2RRX1-MOUSE Actin, beta OS=Mus musculus OX=10090 GN=Actb PE=2 SV=1
MDDDIAALVVDNGSGMCKAGFAGDDAPRAVFPSIVGRPRHQGVMVGMGQKDSYVGDEAQSKR-
GILTLKYPIEHGIVTNWDDMEKIWHHTFYNELRVAPEEHPVLLTEAPLNPKANREKMTQIMFETFNTPAMYVAIQAVLSLYASGRTTGIVMDSGDGVTHTVPIYEGYALPHAILRLDLAGRDLTDYLMKILTERGYSF
TTTAEREIVRDIKEKLCYVALDFEQEMATAASSSSLEKSYELPDGQVITI-
GNERFRCPEALFQPSFLGMESCGIHETTFNSIMKCDVDIRKDLYANTVLSGGTTMYPGIADRMQKEITALAPSTMKIKIIAPPERKYSVWIGGSILASLSTFQQMWISKQEYDESGPSIVHRKCF

>sp|P61089|UBE2N-MOUSE Ubiquitin-conjugating enzyme E2 N OS=Mus musculus OX=10090 GN=Ube2n PE=1 SV=1
MAGLPRRIIKETQRLLAEPVPGIKAEPDESNARYFHVVIAGPQDSPFEGGTFKLELFLPEEY-
PMAAPKVRFMTKIYHPNVDKLGRICLDILKDKWSPALQIRTVLLSIQALLSAPNPDDPLANDVAEQWKTNEAQAIETARAWTRLYAMNNI

>sp|P63001|RAC1-MOUSE Ras-related C3 botulinum toxin substrate 1 OS=Mus musculus OX=10090 GN=Rac1 PE=1 SV=1

MQAIKCVVVGDGAVGKTCLLISYTTNAFPGEYIPTVFDNYSANVMVDGKPVNLGLWDTAGQE-
DYDRLRPLSYPQTDVFLICFSLVSPASFENVRAKWYPEVRHHCPNTPIILVGTKLDLRDDKDTIEKLKEKKLTPITYPQGLAMAKEIGAVKYLECSALTQRGLKTVFDEAIRAVLCPPPVKKRKRKCLLL

>sp|P61205|ARF3-MOUSE ADP-ribosylation factor 3 OS=Mus musculus OX=10090 GN=Arf3 PE=2 SV=2
MGNIFGNLLKSLIGKKEMRILMVGLDAAGKTTILYKLKLGEIVTTIPTIGFNVET-
VEYKNISFTVWDVGGQDKIRPLWRHYFQNTQGLIFVVDSNDRERVNEAREELMRMLAEDELRDAVLLVFANKQDLPNAMNAAEITDKLGLHSLRHRNWYIQATCATSGDGLYEGLDWLANQLKNKK

>sp|P62331|ARF6-MOUSE ADP-ribosylation factor 6 OS=Mus musculus OX=10090 GN=Arf6 PE=1 SV=2
MGKVLSKIFGNKEMRILMLGLDAAGKTTILYKLKLGQSVTTIPTVGFNVET-
VTYKNVKFNVWDVGGQDKIRPLWRHYYTGTQGLIFVVDCADRDRIDEARQELHRIINDREMRDAIILIFANKQDLPDAMKPHEIQEKLGLTRIRDRNWYVQPSCATSGDGLYEGLTWLTSNYKS

>sp|P17182|ENOA-MOUSE Alpha-enolase OS=Mus musculus OX=10090 GN=Eno1 PE=1 SV=3
MSILRIHAREIFDSRGNPTVEVDLYTAKGLFRAAVPSGASTGIYEALELRD-
NDKTRFMGKGVSQAVEHINKTIAPALVSKKVNVVEQEKIDKLMIEMDGTENKSKFGANAILGVSLAVCKAGAVEKGVPLYRHIADLAGNPEVILPVPAFNVINGGSHAGNKLAMQEFMILPVGASSFREAMRIGAE
VYHNLKNVIKEKYGKDATNVGDEGGFAPNILENKEALELLKTAIAKAGYT-
DQVVIGMDVAASEFYRSGKYDLDFKSPDDPSRYITPDQLADLYKSFVQNYPVVSIEDPFDQDDWGAWQKFTASAGIQVVGDDLTVTNPKRIAKAASEKSCNCLLLKVNQIGSVTESLQACKLAQSNGWGVMVSHRS
GETEDTFIADLVVGLCTGQIKTGAPCRSERLAKYNQILRIEEELGSKAKFAGRSFRNPLAK

>sp|P40240|CD9-MOUSE CD9 antigen OS=Mus musculus OX=10090 GN=Cd9 PE=1 SV=2
MPVKGGSKCIKYLLFGFNFIFWLAGIAVLAIGLWLRFDSQTKSIFEQENNHSSFYTGVYILI-
GAGALMMLVGFLGCCGAVQESQCMLGLFFGFLLVIFAIEIAAAVWGYTHKDEVIKELQEFYKDTYQKLRSKDEPQRETLKAIHMALDCCGIAGPLEQFISDTCPKKQLLESFQVKPCPEAISEVFNNKFHIIGAVGIGIA
VVMIFGMIFSMILCCAIRRSREMV

>sp|Q9Z127|LAT1-MOUSE Large neutral amino acids transporter small subunit 1 OS=Mus musculus OX=10090 GN=Slc7a5 PE=1 SV=2
MAVAGAKRRAVATPAAAAAEEERQAREKMLEARRGDGADPEGEGVTLQRNITLLNG-
VAIIVGTIIGSGIFVTPTGVLKEAGSPGLSLVVWAVCGVFSIVGALCYAELGTTISKSGGDYAYMLEVYGSLPAFLKLWIELLIIRPSSQYIVALVFATYLLKPVFPTCPVPEEAAKLVACLCVLLLTAVNCYSVKAATRVQD
AFAAAKLLALALIILLGFIQMGKDMGQGDASNLQQKLSFEGTNLDVGNIVLALYS-
GLFAYGGWNYLNFVTEEMINPYRNLPLAIIISLPIVTLVYVLTNLAYFTTLSTNQMLTSEAVAVDFGNYHLGVMSWIIPVFVGLSCFGSVNGSLFTSSRLFFVGSREGHLPSVLSMIHPQLLTPVPSLVFTCIMTLMYAFSR
DIFSIINFFSFFNWLCVALAIIGMMWLRFKKPELERPIKVNLALPVFFILACLFLIAVSFWKTPMECGIGFAIILSGLPVYFFGVWWKNKPKWILQAIFSVTVLCQKLMQVVPQET

>sp|P16858|G3P-MOUSE Glyceraldehyde-3-phosphate dehydrogenase OS=Mus musculus OX=10090 GN=Gapdh PE=1 SV=2
MVKVGVNGFGRIGRLVTRAAICSGKVEIVAINDPFIDL-
NYMVYMFQYDSTHGKFNGTVKAENGKLVINGKPITIFQERDPTNIKWGEAGAEYVVESTGVFTTMEKAGAHLKGGAKRVIISAPSADAPMFVMGVNHEKYDNSLKIVSNASCTTNCLAPLAKVIHDNFGIVEGLMT
TVHAITATQKTVDGPSGKLWRDGRGAAQNIIPASTGAAKAVGKVIPELNGKLTG-
MAFRVPTPNVSVVDLTCRLEKPAKYDDIKKVVKQASEGPLKGILGYTEDQVVSCDFNSNSHSSTFDAGAGIALNDNFVKLISWYDNEYGYSNRVVDLMAYMASKE

>sp|P68040|RACK1-MOUSE Receptor of activated protein C kinase 1 OS=Mus musculus OX=10090 GN=Rack1 PE=1 SV=3

MTEQMTLRGTLKGHNGWVTQIATTPQFPDMILSASRDKTIIMWKLTRDETNYGIPQRAL-
RGHSHFVSDVVISSDGQFALSGSWDGTLRLWDLTTGTTTRRFVGHTKDVLSVAFSSDNRQIVSGSRDKTIKLWNTLGVCKYTVQDESHSEWVSCVRFSPNSSNPIIVSCGWDKLVKVWNLANCKLKTNHIGHTGYLN
TVTVSPDGSLCASGGKDGQAMLWDLNEGKHLYTLDGGDIINALCFSPNRYWLCAATGPSIKIWDLEGKIIVDELKQEVISTSSKAEPPQCTSLAWSADGQTLFAGYTDNLVRVWQVTIGTR

>sp|P41731|CD63-MOUSE CD63 antigen OS=Mus musculus OX=10090 GN=Cd63 PE=1 SV=2
MAVEGGMKCVKFLLYVLLLAFCACAVGLIAIGVAVQVVLKQAITHETTAGSLLPVVIIAV-
GAFLFLVAFVGCCGACKENYCLMITFAIFLSLIMLVEVAVAIAGYVFRDQVKSEFNKSFQQQMQNYLKDNKTATILDKLQKENNCCGASNYTDWENIPGMAKDRVPDSCCINITVGCGNDFKESTIHTQGCVETIAI
WLRKNILLVAAAALGIAFVEVLGIIFSCCLVKSIRSGYEVM

>sp|P16045|LEG1-MOUSE Galectin-1 OS=Mus musculus OX=10090 GN=Lgals1 PE=1 SV=3
MACGLVASNLNLKPGECLKVRGEVASDAKSFVLNLGKDSNNLCLHFNPRFNAHGDANTIVCNTKEDGTWGTEHREPAFPFQPGSITEVCITFDQADLTIKLPDGHEFKFPNRLNMEAINYMAADGDFKIKCVAFE

>sp|P35762|CD81-MOUSE CD81 antigen OS=Mus musculus OX=10090 GN=Cd81 PE=1 SV=2
MGVEGCTKCIKYLLFVFNFVFWLAGGVILGVALWLRHDPQTTSLLYLELGNK-
PAPNTFYVGIYILIAVGAVMMFVGFLGCYGAIQESQCLLGTFFTCLVILFACEVAAGIWGFVNKDQIAKDVKQFYDQALQQAVMDDDANNAKAVVKTFHETLNCCGSNALTTLTTTILRNSLCPSGGNILTPLLQQD
CHQKIDELFSGKLYLIGIAAIVVAVIMIFEMILSMVLCCGIRNSSVY

Table S1. Functional enrichment data for 11 highly disordered host proteins focusing on gene ontology highlighting biological process.

Protein ID	Biological Process (Go term)	Count in Network	Enrichment Strength	False Discovery Rate
Q9WVE8	1. Negative regulation of membrane tubulation (GO:1903526)	2 of 2	2.56	0.0048
	2. Caveola Assembly (GO:0070836)	3 of 4	2.44	0.00014
	3. Plasma membrane tubulation (GO:0097320)	9 of 14	2.37	6.48e-15
	4. Early endosome to Golgi apparatus (GO:0034498)	4 of 8	2.26	6.77e-06
	5. Lipid tube assembly (GO:0060988)	2 of 4	2.26	0.0106
Q9D8B3	1. Viral budding via host ESCRT complex (GO:0039702)	14 of 14	2.34	3.25e-23
	2. Viral budding from plasma membrane (GO:0046761)	13 of 13	2.34	1.17e-21
	3. Multivesicular body-lysosome fusion (GO:0061763)	11 of 11	2.34	2.33e-18
	4. Vesicle fusion with vacuole (GO:0051469)	11 of 11	2.34	2.33e-18
	5. Protein transport to vacuole involved in ubiquitin-dependent (GO:0043328)	6 of 6	2.34	9.19e-10
O54946	1. Positive regulation of aggrephagy (GO:1905337)	3 of 3	2.51	0.00020
	2. Protein refolding(GO:0042026)	15 of 21	2.36	1.27e-25
	3. Negative regulation of inclusion body assembly (GO:0090084)	6 of 11	2.24	7.57e-09
	4. Telomerase holoenzyme complex assembly (GO:1905323)	2 of 4	2.21	0.0162
	5. Protein insertion into mitochondrial outer membrane (GO:0045040)	2 of 4	2.21	0.0162
Q62167	1. Viral translational termination-reinitiation (GO:0075525)	5 of 5	1.82	1.02e-05
	2. Regulation of polysome binding (GO:1905696)	2 of 2	1.82	0.0355
	3. NLRP1 inflammasome complex assembly (GO:1904784)	2 of 2	1.82	0.0355
	4. Pyroptosome complex assembly (GO:1904270)	2 of 2	1.82	0.0355
	5. Eukaryotic translation initiaition factor 4F complex assembly (GO:0097010)	2 of 2	1.82	0.0355
Q91ZR2	1. Negative regulation of membrane tubulation (GO:1903526)	2 of 2	2.7	0.0038
	2. Lipid tube assembly (GO:0060988)	2 of 4	2.39	0.0084
	3. Cleavage furrow formation (GO:0036089)	3 of 7	2.33	0.00023
	4. Positive regulation of neutrophil apoptotic process (GO:0033031)	2 of 5	2.3	0.0111
	5. Positive regulation of clathrin-dependent endocytosis (GO:2000370)	2 of 6	2.22	0.0138
Q61187	1. Viral budding (GO:0046755)	20 of 20	1.97	1.07e-26
	2. Multivesicular body organization (GO:0036257)	15 of 15	1.97	6.35e-20
	3. Viral budding via host ESCRT complex (GO:0039702)	14 of 14	1.97	1.45e-18

	4. Multivesicular body assembly (GO:0036258)<br>5. Viral budding from plasma membrane (GO:0046761)	14 of 14<br>13 of 13	1.97<br>1.97	1.45e-18<br>3.11e-17
P06837	1. Neurofilament bundle assembly (GO:0033693)<br>2. Trans-synaptic signaling by neuropeptide, modulating synaptic transmission (GO:0099551)<br>3. Taste bud development (GO:0061193)<br>4. Regulation of norepinephrine uptake (GO:0051621)<br>5. Regulation of retinal cell programmed cell death (GO:0046668)	3 of 3<br>2 of 2<br>2 of 2<br>2 of 2<br>4 of 6	2.18<br>2.18<br>2.18<br>2.18<br>2.0	0.00020<br>0.0059<br>0.0059<br>0.0059<br>1.80e-05
P26040	1. Membrane to membrane docking (GO:0022614)<br>2. Paranodal junction maintenance (GO:1990227)<br>3. Terminal web assembly (GO:1902896)<br>4. Beta selection (GO:0043366)<br>5. Positive regulation of protein processing in phagocytic vesicles (GO:1903923)	5 of 5<br>3 of 3<br>3 of 3<br>3 of 3<br>2 of 2	1.89<br>1.89<br>1.89<br>1.89<br>1.89	2.31e-06<br>0.00070<br>0.00070<br>0.00070<br>0.0117
Q91YD9	1. Positive regulation of platelet-derived growth factor receptor-beta signaling pathway (GO:2000588)<br>2. Formin-nucleated actin cable assembly (GO:0070649)<br>3. Meiotic chromosome movement towards spindle pole (GO:0016344)<br>4. Positive regulation of barbed-end actin filament capping (GO:2000814)<br>5. Positive regulation of protein processing in phagocytic vesicle (GO:1903923)	3 of 3<br>3 of 3<br>3 of 3<br>2 of 2<br>2 of 2	1.97<br>1.97<br>1.97<br>1.97<br>1.97	0.00069<br>0.00069<br>0.00069<br>0.0141<br>0.0141
Q8R0J7	1. Viral budding via host ESCRT complex (GO:0039702)<br>2. Viral budding from plasma membrane (GO:0046761)<br>3. Multivesicular body-lysosome fusion (GO:0061763)<br>4. Vesicle fusion with vacuole (GO:0051469)<br>5. Ubiquitin-independent protein catabolic process via the multivesicular body sorting pathway (GO:0090611)	14 of 14<br>13 of 13<br>11 of 11<br>11 off 11<br>5 of 5	2.72<br>2.72<br>2.72<br>2.72<br>2.72	2.64e-29<br>3.38e-27<br>6.86e-23<br>6.86e-23<br>6.21e-10
P63037	1. Positive regulation of aggrephagy (GO:1905337)<br>2. Negative regulation of inclusion body assembly(GO:0090084)<br>3. Protein refolding (GO:0042026)<br>4. Positive regulation of establishment of protein localization to telomere (GO:1904851)<br>5. Telomerase holoenzyme complex assembly (GO:1905323)	3 of 3<br>9 of 11<br>17 of 21<br>8 of 10<br>3 of 4	2.26<br>2.17<br>2.16<br>2.16<br>2.13	0.00047<br>5.83e-13<br>8.51e-26<br>2.62e-11<br>0.00075

Table S2. Functional enrichment data for 11 highly disordered host proteins focusing on their individual gene ontology highlighting Molecular Function.

Protein ID & (Name)		Molecular Function (Go Term)	Count in Network	Enrichment Strength	False Discovery Rate
	1.	D2 dopamine receptor binding (GO:0031749)	2 of 6	2.08	0.0304
Q9WVE8	2.	inositol-1,4,5-trisphosphate 5-phosphatase activity (GO:0052658)	2 of 7	2.02	0.0368
	3.	Nitric-oxide synthase binding (GO:0050998)	4 of 22	1.82	0.00022
Pacsin2	4.	Dopamine receptor binding (GO:0050780)	3 of 25	1.64	0.0111
	5.	G protein activity (GO:0003925)	3 of 39	1.45	
Q9D8B3 (Chmp4b)	1.	MIT domain binding (GO:0090541)	3 of 4	2.21	0.0012
	2.	Structural constituent of eye lens (GO:0005212)	14 of 25	2.09	4.36e-20
	3.	Protein tag (GO:0031386)	4 of 14	1.8	0.00048
	4.	Structural constituent of cytoskeleton (GO:0005200)	14 of 77	1.6	8.50e-15
	5.	Ubiquitin binding (GO:0043130)	9 of 97	1.31	8.22e-07
O54946 (Dnajb6)	1.	CTP binding (GO:0002135)	2 of 2	2.51	0.0059
	2.	ATP-dependent protein disaggregase activity (GO:0140545)	3 of 4	2.38	0.00012
	3.	C3HC4-type RING finger domain binding (GO:0055131)	4 of 6	2.33	2.53e-06
	4.	Sulfonylurea receptor binding (GO:0017098)	2 of 3	2.33	0.0093
	5.	ATP-dependent protein folding chaperone (GO:0140662)	18 of 28	2.31	2.62e-31
Q62167 (Ddx3x)	1.	Cysteine-type endopeptidase activator activity (GO:0140608)	2 of 2	1.82	0.0490
	2.	mRNA cap binding (GO:0098808)	2 of 2	1.82	0.0490
	3.	RNA strand-exchange activity (GO:0034057)	2 of 2	1.82	0.0490
	4.	U5 snRNA binding (GO:0030623)	2 of 2	1.82	0.0490
	5.	CTP binding (GO:0002135)	2 of 2	1.82	0.0490
Q91ZR2 (Snx18)	1.	1-phosphatidylinositol-4-phosphate 3-kinase activity (GO:0035005)	7 of 7	2.7	4.46e-13
	2.	phosphatidylinositol-3,4-bisphosphate 5-kinase activity (GO:0052812)	5 of 6	2.62	6.48e-09
	3.	phosphatidylinositol-4,5-bisphosphate 3-kinase activity (GO:0046934)	5 of 6	2.62	6.48e-09
	4.	1-phosphatidylinositol-3-kinase activity (GO:0016303)	7 of 10	2.54	1.82e-12
	5.	D2 dopamine receptor binding (GO:0031749)	2 of 6	2.22	0.0245
Q61187 (Tsg101)	1.	L-lactate dehydrogenase activity (GO:0004459)	4 of 4	1.97	0.00012
	2.	Atg8-specific peptidase activity (GO:0019786)	2 of 2	1.97	0.0399
	3.	CTP binding (GO:0002135)	2 of 2	1.97	0.0399

	4. MIT domain binding (GO:0090541)	3 of 4	1.85	0.0040
	5. Glyceraldehyde-3-phosphate dehydrogenase (NAD+) (phosphorylating) activity (GO:0004365)	3 of 4	1.85	0.0040
P06837 (Gap43)	1. Calcium ion binding involved in regulation of presynaptic cytosolic calcium ion concentration (GO:0099534)	2 of 2	2.18	0.0245
	2. Neurotrophin TRKB receptor binding (GO:0005169)	2 of 2	2.18	0.0245
	3. Nerve growth factor receptor binding (GO:0005163)	4 of 5	2.08	8.41e-05
	4. Ganglioside GT1b binding (GO:1905576)	2 of 3	2.0	0.0354
	5. Structural constituent of postsynaptic intermediate filament cytoskeleton(GO:0099184)	2 of 3	2.0	0.0345
P26040 (Ezr)	1. Rho GDP-dissociation inhibitor activity (GO:0005094)	3 of 3	1.89	0.0022
	2. Cytoskeletal protein-membrane anchor activity (GO:0106006)	2 of 2	1.89	0.0391
	3. Rho-dependent protein serine/threonine kinase activity (GO:0072518)	2 of 2	1.89	0.0391
	4. Histone kinase activity (H3-T6 specific) (GO:0035403)	2 of 2	1.89	0.0391
	5. CTP binding (GO:0002135)	2 of 2	1.89	0.0391
Q91YD9 (WasL)	1. EH domain binding (GO:1990175)	2 of 2	1.97	0.0336
	2. Rho-dependent protein serine/threonine kinase activity (GO:0072518)	2 of 2	1.97	0.0336
	3. GBD domain binding (GO:0032427)	2 of 2	1.97	0.0336
	4. Profilin binding (GO:0005522)	8 of 12	1.8	2.36e-09
	5. Arp2/3 complex binding (GO:0071933)	9 of 17	1.7	6.28e-10
Q8R0J7 (Vps37b)	1. MIT domain binding (GO:0090541)	3 of 4	2.59	0.00019
	2. Protein tag (GO:0031386)	4 of 14	2.17	4.31e-05
	3. Ubiquitin binding (GO:0043130)	10 of 97	1.73	3.22e-11
	4. Calcium-dependent protein binding (GO:0048306)	4 of 94	1.34	0.0177
	5. Ubiquitin-like protein ligase binding (GO:0044389)	8 of 333	1.1	0.00019
P63037	1. CTP binding (GO:0002135)	2 of 2	2.26	0.0166
	2. ATP-dependent protein folding chaperone (GO:0140662)	24 of 28	2.19	2.00e-38
	3. ATP-dependent protein disaggregase activity (GO:0140545)	3 of 4	2.13	0.00067
	4. dATP binding (GO:0032564)	3 of 4	2.13	0.00067
	5. Adenyl-nucleotide exchange factor activity (GO:0000774)	8 of 11	2.12	1.13e-11

Table S3. Functional enrichment data for 11 highly disordered host proteins focusing on their individual gene ontology highlighting Cellular Component.

Protein ID & (Name)	Cellular Component (Go Term)	Count in Network	Enrichment Strength	False Discovery Rate
Q9WVE8<br>Pacsin2	1. Ciliary pocket membrane (GO:0020018)<br>2. Caveolar macromolecular signaling complex (GO:0002095)<br>3. Extrinsic component of synaptic vesicle membrane (GO:0098850)<br>4. Myb complex (GO:0031523)<br>5. Presynaptic endocytic zone (GO:0098833)	2 of 3<br>2 of 3<br>6 of 10<br>2 of 4<br>5 of 13	2.39<br>2.39<br>2.34<br>2.26<br>2.15	0.0018<br>0.0018<br>2.63e-10<br>0.0025<br>6.35e-08
Q9D8B3<br>(Chmp4b)	1. ESCRT complex (GO:0036452)<br>2. Amphisome membrane (GO:1904930)<br>3. ESCRT I complex (GO:0000813)<br>4. ESCRT III complex (GO:0000815)<br>5. SCRT II complex (GO:0000814)	26 of 26<br>11 of 11<br>11 of 11<br>10 of 10<br>3 of 3	2.34<br>2.34<br>2.34<br>2.34<br>2.34	3.59e-45<br>7.70e-19<br>7.70e-19<br>3.42e-17<br>6.54e-05
O54946<br>(Dnajb6)	1. Chaperonin-containing T-complex (GO:0005832)<br>2. Zona pellucida receptor complex (GO:0002199)<br>3. PAM complex, Tim23 associated import motor (GO:0001405)<br>4. Chaperone complex (GO:0101031)<br>5. Endoplasmic reticulum chaperone complex (GO:0034663)	4 of 10<br>4 of 11<br>2 of 6<br>10 of 36<br>3 of 12	2.11<br>2.07<br>2.03<br>1.95<br>1.9	5.86e-06<br>7.46e-06<br>0.0090<br>1.59e-13<br>0.00054
Q62167<br>(Ddx3x)	1. Inflammasome complex (GO:0061702)<br>2. Eukaryotic translation initiation factor 3 complex (GO:0005852)<br>3. Eukaryotic 48S preinitiation complex (GO:0033290)<br>4. NLRP3 inflammasome complex (GO:0072559)<br>5. PAF inflammasome complex (GO:0072557)	21 of 21<br>16 of 16<br>15 of 15<br>8 of 8<br>8 of 8	1.82<br>1.82<br>1.82<br>1.82<br>1.82	1.04e-25<br>1.62e-19<br>2.60e-18<br>1.35e-09<br>1.35e-09
Q91ZR2<br>(Snx18)	1. Retromer, cargo-selective complex (GO:0030906)<br>2. tubular endosome (O:0097422)	3 of 3<br>4 of 5<br>4 of 6<br>2 of 4	2.7<br>2.6<br>2.52<br>2.39	1.19e-05<br>2.60e-07<br>4.04e-07<br>0.0023

	3. Phosphatidylinositol 3-kinase complex, class IA (GO:0005943)	8 of 21	2.28	1.74e-13
	4. Phosphatidylinositol 3-kinase complex, class IB (GO:0005944)			
	5. Retromer complex (GO:0030904)			
Q61187 (Tsg101)	1. ESCRT complex (GO:0036452)	26 of 26	1.97	7.72e-36
	2. Amphisome membrane (GO:1904930)	11 of 11	1.97	4.24e-15
	3. ESCRT I complex (GO:0000813)	11 of 11	1.97	4.24e-15
	4. ESCRT III complex (GO:0000815)	10 of 10	1.97	9.96e-14
	5. Retromer, cargo-selective complex (GO:0030906)	3 of 3	1.97	0.00033
P06837 (Gap43)	1. Presynaptic intermediate filament cytoskeleton (GO:0099182)	2 of 2	2.18	0.0037
	2. Postsynaptic intermediate filament cytoskeleton (GO:0099160)	3 of 4	2.05	0.00021
	3. Dendritic branch (GO:0044307)	4 of 6	2.0	1.04e-05
	4. Neurofibrillary tangle (GO:0097418)	3 of 5	1.96	0.00033
	5. Dendritic filopodium (GO:1902737)	3 of 6	1.88	0.00045
P26040 (Ezr)	1. Stereocilium base (GO:0120044)	5 of 5	1.89	1.38e-06
	2. HSP90-CDC37 chaperone complex (GO:1990565)	2 of 2	1.89	0.0105
	3. Upper tip-link density (GO:1990435)	2 of 2	1.89	0.0105
	4. Septate junction (GO:0005918)	2 of 2	1.89	0.0105
	5. Schwann cell microvillus (GO:0097454)	2 of 2	1.71	0.0162
Q91YD9 (WasL)	1. Arp2/3 protein complex (GO:0005885)	9 of 9	1.97	2.05e-12
	2. Actin cap (GO:0030478)	4 of 4	1.97	1.56e-05
	3. Peripheral region of growth cone (GO:0090725)	3 of 3	1.97	0.00036
	4. Podosome core (GO:0061825)	3 of 3	1.97	0.00036
	5. Tubulobulbar complex (GO:0036284)	3 of 3	1.97	0.00036
Q8R0J7 (Vps37b)	1. ESCRT complex (GO:0036452)	26 of 26	2.72	9.78e-58
	2. Amphisome membrane (GO:1904930)	11 of 11	2.72	3.41e-23
	3. ESCRT I complex (GO:0000813)	11 of 11	2.72	3.41e-23
	4. ESCRT III complex (GO:0000815)	10 of 10	2.72	3.97e-21
	5. ESCRT II complex (GO:0000814)	3 of 3	2.72	4.55e-06

P63037	1. HSP90-CDC37 chaperone complex (GO:1990565)<br>2. Chaperonin-containing T-complex (GO:0005832)<br>3. Zona pellucida receptor complex (GO:0002199)<br>4. Chaperone complex (GO:0101031)<br>5. PAM complex, Tim23 associated import motor (GO:0001405)	2 of 2<br>8 of 10<br>8 of 11<br>20 of 36<br>2 of 6	2.26<br>2.16<br>2.12<br>2.0<br>1.78	0.0097<br>1.44e-11<br>1.98e-11<br>1.75e-28<br>0.0313

Table S4. Multifactorial analysis of intrinsic disorder predisposition of mouse proteins entrapped in RABV particles.

Sequence ID	Gene Name	PONDR VLXT, %	PONDR VLXT, Score	PONDR VSL2, %	PONDR VSL2, Score	PONDR VL3, %	PONDR VL3, Score	IU-Pred_Short, %	IU-Pred_Short, Score	IU-Pred_Long, %	IU-Pred_Long, Score	PONDR FIT, %	PONDR FIT, Score	MDP, %	MDP, Score	dCDF	dCH	Node Degree	PLLPS
P06837	Gap43	90.75	0.8237	100	0.97094	100	0.92615	98.68	0.7291	99.56	0.90544	100	0.8702	100	0.87092	−0.4218	0.22313	3	0.9949
O54946	Dnajb6	50.68	0.50954	96.44	0.82287	89.59	0.73547	43.29	0.50606	52.33	0.59267	76.16	0.68979	66.58	0.64273	−0.1063	0.04155	3	0.9937
Q9DB34	Chmp2a	91.89	0.80524	84.68	0.7787	89.19	0.75691	26.58	0.43193	43.69	0.47228	91.89	0.78788	87.84	0.67216	−0.4015	−0.0662	9	0.4463
Q8R0J7	Vps37b	75.09	0.6758	80.35	0.71006	76.84	0.68684	35.09	0.42488	46.67	0.50847	50.18	0.55174	64.56	0.59297	−0.272	−0.1387	9	0.7062
Q91YD9	Wasl	60.68	0.59373	70.46	0.67398	69.06	0.65377	62.08	0.50558	72.46	0.66646	59.28	0.53035	64.47	0.60398	−0.1877	3.6E−05	8	0.9796
P63024	Vamp3	47.57	0.44546	66.02	0.49674	49.51	0.51802	24.27	0.31274	15.53	0.23886	44.66	0.52867	19.42	0.33708	−0.0471	−0.233	8	0.2109
Q9WVE8	Pacsin2	41.36	0.43913	62.14	0.62508	55.97	0.5258	34.16	0.40153	44.44	0.49655	48.97	0.47636	53.5	0.49408	−0.0395	0.09151	3	0.7374
P63037	Dnaja1	28.72	0.34943	61.46	0.54464	63.73	0.55642	22.42	0.33609	20.91	0.38103	27.96	0.35151	34.76	0.41985	0.05144	−0.0354	5	0.3089
P26040	Ezr	55.8	0.49604	56.14	0.59049	51.02	0.47935	32.94	0.34698	44.54	0.42032	51.88	0.48866	51.02	0.47031	−0.0977	0.05128	16	0.5557
P63101	Ywhaz	44.9	0.42135	53.88	0.50166	50.61	0.44431	10.2	0.29499	12.65	0.33685	27.76	0.38217	13.88	0.32284	−0.0212	−0.0119	13	0.1834
Q61187	Tsg101	51.15	0.49621	53.45	0.53086	49.62	0.44529	12.28	0.26785	30.43	0.33599	47.83	0.43953	48.85	0.41929	−0.0936	−0.1241	19	0.5314
P11499	Hsp90ab1	31.63	0.36826	45.17	0.49574	37.02	0.44923	15.88	0.2935	22.24	0.34964	21.55	0.32532	24.72	0.38028	0.03085	−0.0006	25	0.3785
Q91ZR2	Snx18	43	0.42843	44.95	0.52868	31.76	0.43592	28.01	0.35376	33.06	0.40678	37.13	0.40634	35.34	0.42665	−0.0301	−0.0815	1	0.763
Q9WU78	Pdcd6ip	48.33	0.45406	44.53	0.51719	39.47	0.44568	16.11	0.29251	20.14	0.36149	26.58	0.35033	30.72	0.40354	−0.0531	−0.1177	16	0.346
Q62167	Ddx3x	31.57	0.37903	40.63	0.5043	35.35	0.40918	24.17	0.3152	25.68	0.35973	33.38	0.38935	33.23	0.3928	0.01955	−0.0726	2	0.845
Q4VAE6	Rhoa	31.09	0.35982	39.9	0.41219	43.01	0.44401	16.06	0.28989	11.92	0.29392	22.8	0.29548	26.42	0.34922	0.0387	−0.1405	17	0.264
P63017	Hspa8	25.23	0.33332	38.7	0.45257	29.26	0.3437	10.06	0.30085	20.43	0.37374	21.05	0.28628	21.98	0.34841	0.06746	−0.1047	27	0.3235
Q9D1C8	Vps28	22.62	0.31164	38.01	0.41881	27.6	0.34358	9.5	0.25107	1.81	0.25115	13.12	0.2777	1.81	0.25173	0.08547	−0.1076	9	0.4616
P63168	Dynll1	19.1	0.23101	37.08	0.41573	31.46	0.31438	8.99	0.22919	0	0.16193	28.09	0.35668	24.72	0.28482	0.16282	−0.1259	7	0.1091
P10852	Slc3a2	24.14	0.28345	37.07	0.40321	25.29	0.32542	9.32	0.22888	7.22	0.22491	14.83	0.26242	12.55	0.28805	0.1131	−0.1816	3	0.2208
P60335	Pcbp1	46.35	0.46959	36.52	0.45513	16.01	0.34592	8.71	0.32671	15.17	0.3748	13.48	0.33537	7.3	0.32693	−0.0695	−0.2272	7	0.3965
P46467	Vps4b	31.76	0.35627	36.04	0.47074	25	0.38447	14.19	0.30692	20.5	0.37041	20.5	0.30462	19.37	0.36557	0.04241	−0.1004	10	0.5084
Q99J93	Ifitm2	22.22	0.26329	34.72	0.3316	22.22	0.24907	11.11	0.18773	0	0.12423	22.22	0.3174	5.56	0.20404	0.12956	−0.3834	1	0.1254
P63242	Eif5a	22.08	0.28374	33.77	0.41635	18.83	0.37768	8.44	0.30059	2.6	0.29006	16.88	0.31493	5.19	0.26761	0.11276	−0.1363	9	0.2982
P18760	Cfl1	24.7	0.29399	33.73	0.43418	29.52	0.44383	6.63	0.23865	0	0.22483	21.69	0.30263	5.42	0.24905	0.10466	−0.1315	16	0.2105
P0CG50	Ubc	34.47	0.35876	30.93	0.41033	19.07	0.36646	19.75	0.30588	15.94	0.35598	2.86	0.20281	0	0.27229	0.04093	−0.1113	18	0.1552
O35566	Cd151	2.77	0.10503	27.67	0.26758	9.88	0.25232	5.53	0.1238	0	0.06807	11.46	0.22188	1.58	0.13106	0.28721	−0.3664	6	0.174
P17742	Ppia	11.59	0.22616	26.83	0.39369	10.37	0.28484	10.37	0.33042	2.44	0.31635	12.8	0.27073	0.61	0.25623	0.16995	−0.139	13	0.2871
Q9R0P5	Dstn	19.39	0.29191	26.67	0.37146	0	0.31259	6.06	0.20864	0	0.1693	14.55	0.26307	2.42	0.2174	0.10817	−0.1706	4	0.1597
P35278	Rab5c	17.13	0.26715	24.07	0.40612	23.61	0.27882	18.52	0.25911	18.52	0.30265	23.61	0.29055	18.52	0.25426	0.13292	−0.1584	5	0.2693
Q3UFR4	Slc1a5	28.47	0.2837	21.08	0.28862	16.76	0.28279	11.35	0.13422	4.68	0.11005	12.61	0.23475	16.22	0.22236	0.11061	−0.4538	5	0.2124
P99024	Tubb5	20.95	0.25416	18.92	0.35091	7.88	0.21994	10.36	0.27052	8.11	0.29566	7.88	0.2135	8.56	0.26745	0.14331	−0.1001	10	0.2479
P51150	Rab7	13.53	0.20495	18.36	0.34004	17.39	0.23029	4.83	0.21137	0	0.205	12.08	0.21336	5.31	0.19579	0.1912	−0.1475	17	0.1258
O08992	Sdcbp	23.41	0.30192	17.06	0.35682	10.37	0.2907	4.01	0.21475	0.67	0.24411	12.37	0.23968	1.67	0.22621	0.09531	−0.2542	12	0.1387
B2RRX1	Actb	28	0.32049	16.27	0.33595	0	0.2148	5.6	0.23147	2.67	0.25109	10.93	0.21446	1.33	0.22558	0.07841	−0.1696	24	0.288
P61089	Ube2n	38.16	0.37761	15.79	0.33684	3.95	0.24102	13.16	0.28606	6.58	0.26415	15.13	0.24233	2.63	0.25116	0.0241	−0.1733	2	0.1846
P63001	Rac1	15.62	0.19482	15.1	0.29621	7.81	0.21206	3.12	0.16615	0	0.16694	6.77	0.19189	1.04	0.16934	0.201	−0.2058	13	0.1137
P61205	Arf3	9.39	0.16719	14.92	0.29522	0	0.16731	3.87	0.14742	0	0.15249	9.39	0.20174	0.55	0.16067	0.22906	−0.1874	2	0.1064
P62331	Arf6	8.57	0.18214	14.86	0.29959	0	0.13135	4.57	0.17894	0	0.17107	12.57	0.20626	2.29	0.173	0.21403	−0.1285	15	0.0996
P17182	Eno1b	13.13	0.21537	14.52	0.33887	5.3	0.23471	2.3	0.21846	0.23	0.24289	5.76	0.18902	2.07	0.20077	0.18369	−0.1967	8	0.1539
P40240	Cd9	10.18	0.12461	14.16	0.24658	0	0.25687	1.33	0.08551	0	0.04249	11.95	0.24936	2.21	0.12476	0.26601	−0.4149	14	0.1103
Q9Z127	Slc7a5	11.13	0.12935	13.87	0.22891	7.62	0.21148	8.2	0.07664	4.3	0.05904	10.55	0.21084	8.98	0.15271	0.25943	−0.476	2	0.24

P16858	Gapdh	7.81	0.17668	12.01	0.31873	0	0.14148	4.2	0.22182	2.1	0.24409	7.21	0.18807	0.3	0.19157	0.21884	−0.226	25	0.1226
P68040	Rack1	5.99	0.1372	11.67	0.30795	0	0.12645	4.1	0.21255	0.63	0.21761	6.31	0.18839	2.52	0.17728	0.25839	−0.1827	13	0.1252
P41731	Cd63	2.52	0.06168	11.34	0.21189	0	0.26451	0.42	0.07787	0	0.03977	7.56	0.22148	1.26	0.10212	0.32679	−0.4907	14	0.1011
P16045	Lgals1	2.96	0.21176	7.41	0.33871	10.37	0.27574	12.59	0.26589	5.19	0.23595	9.63	0.21734	0	0.21161	0.18463	−0.1449	2	0.1439
P35762	Cd81	0.42	0.06865	5.93	0.19764	0	0.21627	0.42	0.05324	0	0.02717	9.75	0.1922	0	0.08982	0.31727	−0.4642	16	0.106

Table S8. Localization of ELMs (Eukaryotic Linear Motifs) within the Droplet Promoting Regions, Aggregation Hot-spots and MoRFs of mouse Neuromodulin (UniProt ID: P06837).

Region Type	Region Range	ELM ID	Position
**Droplet Promoting Region**	**52–227**	CLV_C14_Caspase3-7	197–201
		DOC_USP7_MATH_1,	119–123<br>190–194<br>207–211
		DOC_WW_Pin1_4	169–174<br>139–144<br>93–98
		LIG_BIR_III_2	90–94<br>118–122
		LIG_PDZ_Class_3	222–227
		LIG_TRAF6_MATH_1,	184–192
		LIG_WD40_WDR5_VDV_2,	58–64<br>63–66<br>95–99<br>96–99<br>130–137<br>131–137<br>132–137<br>133–137<br>154–161<br>155–161<br>215–222<br>218–222<br>219–222
		MOD_CDK_SPK_2	93–98<br>139–146
		MOD_CDK_SPxxK_3	
		MOD_CK1_1	86–92<br>128–134<br>142–148<br>190–196

		MOD_GlcNHglycan	84–88<br>85–88<br>127–130<br>132–135<br>209–212
		MOD_GSK3_1	135–142<br>186–193
		MOD_PIKK_1	190–196
		MOD_Plk_2-3	107113
		MOD_ProDKin_1	169–175<br>139–145<br>93–99
		MOD_SUMO_for_1	152–155<br>97–100
		MOD_SUMO_rev_2	118–126<br>122–126<br>149–159<br>154–159<br>191–201<br>192–201<br>193–201<br>196–201<br>198–207<br>200–207<br>203–207
**Aggregation Hot-spot**	52–66	LIG_WD40_WDR5_VDV_2	58–64<br>63–66
**MoRF**	58–81	LIG_WD40_WDR5_VDV_2	58–64<br>63–66
	85–100	DOC_WW_Pin1_4<br>LIG_BIR_III_2	93–98<br>90–94
		LIG_WD40_WDR5_VDV_2	95–99<br>96–99
		MOD_CDK_SPK_2,	93–98
		MOD_CK1_1	86–92
		MOD_ProDKin_1	93–99
		MOD_SUMO_for_1	97–100
**MoRF**	116–227	CLV_C14_Caspase3-7	197–201
		DOC_USP7_MATH_1	119–123<br>190–194<br>207–211
		DOC_USP7_UBL2_3,	153–158

	DOC_WW_Pin1_4	139–144
		169–174
	LIG_BIR_III_2	118–122
	LIG_PDZ_Class_3	222–227
	LIG_TRAF6_MATH_1	184–192
	LIG_WD40_WDR5_VDV_2	130–137
		131–137
		132–137
		133–137
		154–161
		155–161
		215–222
		218–222
		219–222
	MOD_CDK_SPK_2	93–98
	MOD_CDK_SPxxK_3	139–146
	MOD_CK1_1	128–134
		142–148
	MOD_CK2_1	142–148
		190–196
	MOD_GlcNHglycan	127–130
		132–135
		209–212
	MOD_GSK3_1	135–142 & 186–193
	MOD_PIKK_1	190–196
	MOD_ProDKin_1	169–175 & 139–145
	MOD_SUMO_rev_2	152–155
		118–126
		122–126
		149–159
		154–159
		191–201
		192–201
		198–207
		200–207

Table S9. Distribution of ELMs (Eukaryotic Linear Motifs) in Droplet Promoting Regions, Aggregation Hot-spots, regions, and MoRFs (Molecular recognition features) of the protein Chmp4b (UniProt ID: Q9D8B3).

Region Type	Region Range	ELM ID	Position
**Aggregation hotspot**	**54–62**	DOC_USP7_UBL2_3	50–60
**Aggregation hotspot**	**197–207**	DOC_USP7_MATH_1	198–202
		DOC_USP7_UBL2_3	202–206
		IG_SH3_2	200–205
		LIG_SH3_4	202–209
		LIG_SH3_3	197–203
		LIG_SH3_3	194–200
**Aggregation hotspot**	**211–217**	MOD_SUMO_rev_2	208–217
		MOD_SUMO_rev_2	209–217
		MOD_SUMO_rev_2	210–217
		MOD_SUMO_rev_2	211–217
		MOD_SUMO_rev_2	212–217
**MORF regions**	**108–118**	CLV_PCSK_SKI1_1	114–118
		LIG_PTB_Apo_2	105–112
		LIG_PTB_Phospho_1	105–111
		LIG_SH2_STAP1	111–115
		LIG_WD40_WDR5_VDV_2	111–115
**MORF regions:**	**141–200**	DOC_PP1_RVXF_1	149–156
		LIG_FHA_1	186–192
		LIG_Pex14_2	155–159
		LIG_SH3_3	186–192
		LIG_SH3_3	189–195
		LIG_SH3_3	194–200
		MOD_GSK3_1	143–150
		MOD_CK2_1	181–187
**Droplet-Promoting Region:**	1–22	LIG_BIR_II_1	1–5
		DOC_WW_Pin1_4	18–23
		LIG_LIR_Nem_3	2–7
		LIG_Pex14_2	4–8
	190–224	LIG_SH3_4	202–209
		MOD_SUMO_rev_2	208–217<br>209–217<br>210–217<br>211–217<br>212–217
		CLV_PCSK_SKI1_1	202–206
		DOC_USP7_MATH_1	198–202
		LIG_SH3_4	202–209

		LIG_SH3_3	197–203<br>194–200
		LIG_SH3_2	200–205
		DOC_USP7_UBL2_3	202–206

Table S10. Distribution of ELMs (Eukaryotic Linear Motifs) in droplet promoting regions, aggregation hot-spots, regions with multiplicity of binding modes and MoRF (Molecular recognition features) of protein DnaJ homolog subfamily B member 6 (UniProt ID: O54946).

Region Type	Region Range	ELM ID	Position
**Droplet-Promoting Region:**	58–94	DOC_PP4_FxxP_1	84–87
		DOC_WW_Pin1_4	83–88
**Droplet-Promoting Region:**	119–185	CLV_NRD_NRD_1	127–129
		CLV_PCSK_KEX2_1	127–129
		DOC_USP7_MATH_1	164–168
		DOC_WW_Pin1_4	160–165
		LIG_AP2alpha_1,	116–120
		LIG_AP2alpha_1	120–124
		LIG_AP2alpha_2	118–120
		LIG_Arc_Nlobe_1,	148–152
		LIG_Arc_Nlobe_1	155–159
**Droplet-Promoting Region:**	233–365	CLV_NRD_NRD_1	245–247
		CLV_PCSK_KEX2_1,	245–247
		CLV_PCSK_KEX2_1,	345–347
		CLV_NRD_NRD_1	345–347
		CLV_PCSK_PC1ET2_1,	345–347
		DEG_ODPH_VHL_1	253–264
		DEG_SCF_FBW7_1,	271–278
		DEG_SCF_FBW7_1	273–278
		DEG_SCF_FBW7_1	275–282
		DEG_SCF_FBW7_1<br>DEG_SCF_FBW7_1	277–282<br>287–292
		DOC_ANK_TNKS_1	323–330
		DOC_CKS1_1	248–253
		DOC_USP7_MATH_1,	291–295<br>293–297<br>334–338
		DOC_USP7_UBL2_3	310–314<br>341–345<br>348–352<br>352–356<br>358–362
		DOC_WW_Pin1_4	247–252

			271–276
			275–280
			279–284
			287–292
**Aggregation hotspot:**	83–90	DOC_PP4_FxxP_1	84–87
		DOC_WW_Pin1_4	83–88
	105–114	LIG_AP2alpha_2	109–111
	**119–131**	CLV_PCSK_KEX2_1	127–129
		CLV_NRD_NRD_1	127–129
		LIG_AP2alpha_1<br>LIG_AP2alpha_1	116–120<br>120–124
	156–158	DOC_USP7_MATH_1	164–168
		DOC_WW_Pin1_4	160–165
		LIG_Arc_Nlobe_1	155–159
	241–250	CLV_NRD_NRD_1	245–247
		CLV_PCSK_KEX2_1	245–247
		DOC_CKS1_1	248–253
		DOC_WW_Pin1_4	247–252
	345–353	CLV_NRD_NRD_1	345–347
		CLV_PCSK_KEX2_1	345–347
		CLV_PCSK_PC1ET2_1	345–347
		DOC_USP7_UBL2_3	348–352
MoRF	223–278	DOC_CKS1_1	248–253
		CLV_NRD_NRD_1	245–257
		CLV_PCSK_KEX2_1	245–247
		CLV_PCSK_SKI1_1	226–230
		DEG_ODPH_VHL_1	253–264
		DEG_SCF_FBW7_1	271–278
		DEG_SCF_FBW7_1	247–252
		DOC_WW_Pin1_4<br>DOC_WW_Pin1_4	271–276
	282–298	DOC_USP7_MATH_1	291–295
			293–297
		DOC_WW_Pin1_4	287–292
	305–365	CLV_NRD_NRD_1	345–347
		CLV_PCSK_KEX2_1	345–347
		CLV_PCSK_PC1ET2_1	345–347
		DOC_ANK_TNKS_1	323–330
		DOC_USP7_MATH_1	334–338
		DOC_USP7_UBL2_3	310–314
			341–345

	348–352
	352–356
	358–362

Table S11. Distribution of ELMs (Eukaryotic Linear Motifs) in droplet promoting regions, aggregation hot-spots, regions with multiplicity of binding modes and MoRF (Molecular recognition features) of Vps37B protein (UniProt ID: Q8R0J7).

Region Type	Region Range	ELM ID	Position
**Droplet Promoting Region**	157–253	DOC_WW_Pin1_4,	181–186
			218–223
		DOC_WW_Pin1_4	218–230
		CLV_PCSK_KEX2_1	205–207
		CLV_NRD_NRD_1	205–207
		DOC_CKS1_1	219–224
		DOC_CKS1_1	219–225
		DOC_WW_Pin1_4	181–186
		DOC_WW_Pin1_4	218–230
		CLV_PCSK_KEX2_1	205–207
		CLV_NRD_NRD_1,	205–207
		DOC_CKS1_1	219–224
		DOC_CKS1_1	219–225
		DOC_USP7_MATH_1	226–230
		LIG_LYPXL_yS_3	235–238
		LIG_SH3_3	172–178
			175–181
			177–183
			205–211
			206–21
			207–213
			209–215
			216–22
			238–244
			239–245
			244–250
		LIG_SH3_2	241–246
		LIG_SH3_1	205–211
		LIG_SH3_1	206–212
		LIG_TYR_ITIM	189–194
		LIG_WD40_WDR5_VDV_2	182–188
			184–188
			187–194
			191–196
			193–196
			195–202
			197–202
			229–234

	244–285	DEG_Cend_TRIM7_1	282–285
		DEG_CRBN_cyclicCter_1	
		DOC_CYCLIN_yCln2_LP_	259–265
		LIG_Arc_Nlobe_1	279–283
		DOC_WW_Pin1_4	258–263
		DOC_PP2B_PxIxIT_1	280–285
		LIG_SH3_2	265–270
		LIG_SH3_1	274–280
		LIG_SH3_3	259–265
			262–268
			265–271
		LIG_WW_2	241–24
		LIG_WW_3	271–275
		MOD_GlcNHglycan	255–285
		MOD_PIKK_1	248–254
**Aggregation Hot-Spots:**	191–213	CLV_PCSK_KEX2_1	205–207
		LIG_SH3_1	205–211
		LIG_SH3_1	206–212
		CLV_NRD_NRD_1	205–207
		LIG_LYPXL_yS_3	191–194
		MOD_GSK3_1	190–197
	218–224	DOC_CKS1_1	219–224
		DOC_CKS1_1	219–225
		DOC_USP7_MATH_1	226–230
		DOC_WW_Pin1_4	218–230
	228–237	LIG_LIR_Nem_3	233–238
MoRF	188–202	LIG_LYPXL_yS_3	191–194
	218–242	DOC_WW_Pin1_4	218–223
		DOC_CKS1_1	219–225
		DOC_USP7_MATH_1	226–230
		LIG_LIR_Nem_3	233–238
		LIG_LYPXL_yS_3	235–238
	188–242<br>218–242	DOC_USP7_MATH_1	190–194
		DOC_CKS1_1	219–225
		DOC_USP7_MATH_1	226–230
		DOC_WW_Pin1_4	218–223
		LIG_LIR_Nem_3	233–238
		CLV_PCSK_KEX2_1	205–207
		CLV_NRD_NRD_1	205–207
		LIG_LYPXL_yS_3	235–238
		LIG_LYPXL_yS_3	191–194

		DOC_CKS1_1	219–225
		DOC_USP7_MATH_1	226–230
		DOC_WW_Pin1_4	218–223
		LIG_LIR_Nem_3	233–238
		LIG_LYPXL_yS_3	235–238
	249–263	DOC_WW_Pin1_4	258–263
	279–285	DEG_CRBN_cyclicCter_1	282–285
		LIG_Arc_Nlobe_1	279–283

Table S12. Distribution of ELMs (short linear functional motifs) within the sequence of the mouse Wasl protein (UniProt ID: Q91YD9).

Region type	Region range	ELM ID	Position
**Droplet promoting Region**	127–165	CLV_NRD_NRD_1	139–141
		CLV_NRD_NRD_1	270–272
		CLV_PCSK_KEX2_1	137–139
		CLV_PCSK_KEX2_1	144–146
		CLV_PCSK_KEX2_1	145–147
		CLV_PCSK_PC7_1	140–146
		CLV_PCSK_PC1ET2_1	144–146
		LIG_SH3_3	146–152<br>149–155
		LIG_SH3_1	146–152
		LIG_WD40_WDR5_VDV_2	158–161<br>160–166
		MOD_Cter_Amidation	135–138
		MOD_PKA_2	139–145
		TRG_ER_diArg_1	137–140
		LIG_FHA_2	156–162
**Droplet promoting Region**	194–222	CLV_PCSK_KEX2_1	193–195
		CLV_PCSK_PC1ET2_1,	193–195
		CLV_PCSK_SKI1_1<br>MOD_PKA_2	193–197<br>193–199
**Droplet promoting Region**	258–403	CLV_PCSK_KEX2_1	270–272
		DOC_USP7_MATH_1	396–400<br>346–350
		DOC_PP4_MxPP_1	339–342
		DOC_USP7_MATH_1	291–295
		TRG_Oom_RxLR_1	266–270
		TRG_ER_diArg_1	269–271
		CLV_NRD_NRD_1<br>LIG_EVH1_1	270–272<br>340–344
		LIG_FHA_2	257–263

		LIG_PROFILIN_1	284–290<br>285–291<br>355–361<br>369–375<br>370–376<br>371–377<br>372–378<br>374–380
**Droplet promoting Region**	444–501	CLV_PCSK_KEX2_1<br>& CLV_PCSK_PC1ET2_1	473–475<br>473–475
		DEG_SCF_FBW7_1	454–460 & 455–460
		DOC_CKS1_1	454–459
		DOC_USP7_MATH_1	477–481<br>450–455
		DOC_WW_Pin1_4	453–458
		LIG_FHA_2	458–464
		LIG_GBD_Chelix_1	463–471
**Aggregation Hot-Spots**	470–483	DOC_USP7_MATH_1	477–481
**MoRF**	458–482	CLV_PCSK_PC1ET2_1	473–475
		DOC_USP7_MATH_1	477–481
		DOC_USP7_MATH_1	477–481
		LIG_FHA_1	458–464

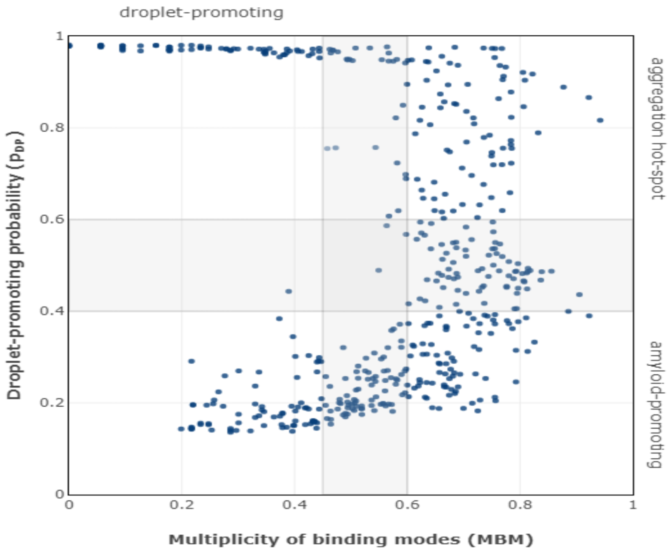
droplet-promoting
aggregation hot-spot
amyloid-promoting
Droplet-promoting probability (p_{DP})
Multiplicity of binding modes (MBM)

(**a**)

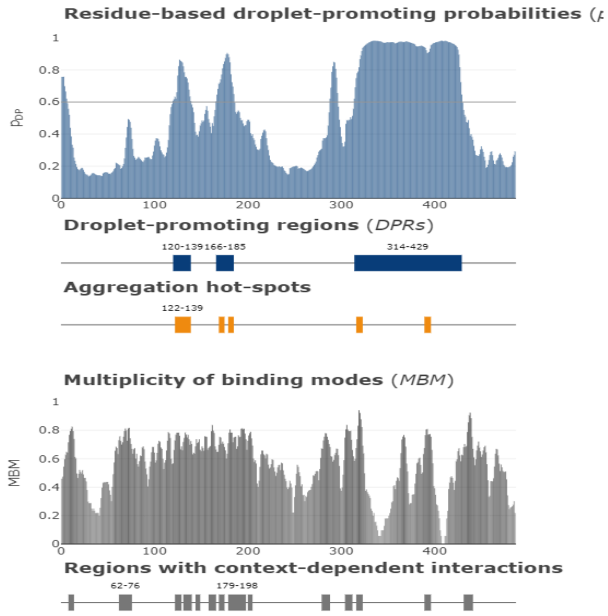
Residue-based droplet-promoting probabilities
Droplet-promoting regions (DPRs)
120-139
166-185
314-429
Aggregation hot-spots
122-139
Multiplicity of binding modes (MBM)
Regions with context-dependent interactions
62-76
179-198

(**b**)

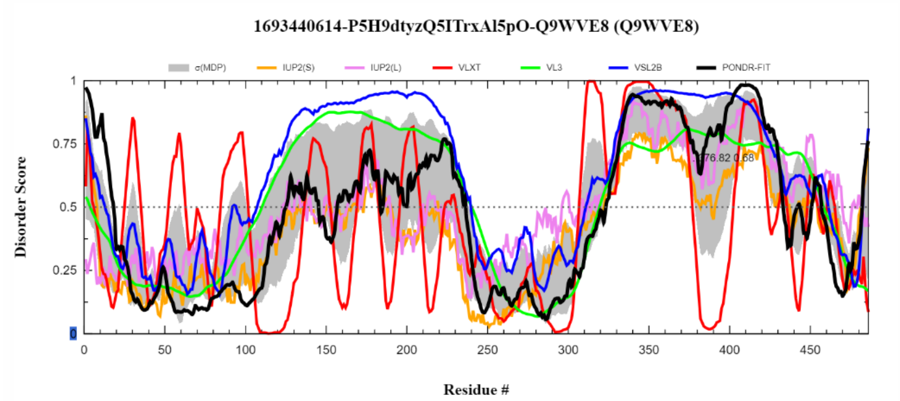
1693440614-P5H9dtyzQ5ITrxAl5pO-Q9WVE8 (Q9WVE8)
σ(MDP)
IUP2(S)
IUP2(L)
VLXT
VL3
VSL2B
PONDR-FIT
Disorder Score
Residue #
1
0.75
0.5
0.25
0
50
100
150
200
250
300
350
400
450

(c)

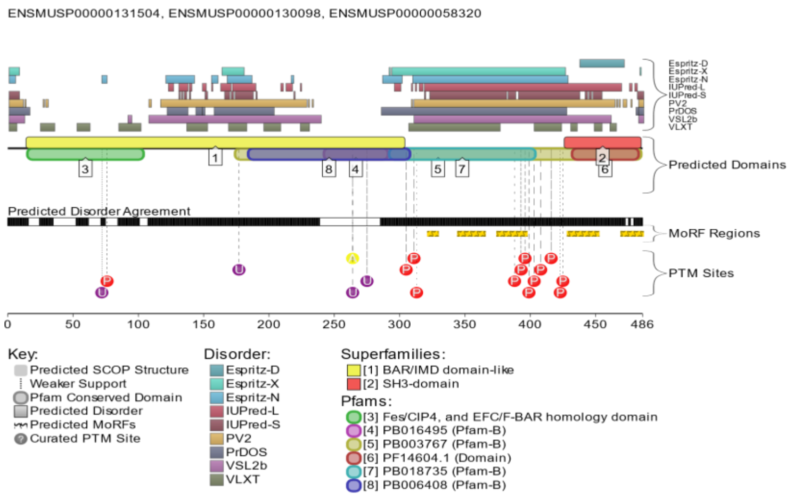
ENSMUSP00000131504, ENSMUSP00000130098, ENSMUSP00000058320
Espritz-D
Espritz-X
Espritz-N
IUPred-L
IUPred-S
PV2
PrDOS
VSL2b
VLXT
Predicted Domains
Predicted Disorder Agreement
MoRF Regions
PTM Sites
0
50
100
150
200
250
300
350
400
450
486
Key:
Predicted SCOP Structure
Weaker Support
Pfam Conserved Domain
Predicted Disorder
Predicted MoRFs
Curated PTM Site
Disorder:
Espritz-D
Espritz-X
Espritz-N
IUPred-L
IUPred-S
PV2
PrDOS
VSL2b
VLXT
Superfamilies:
[1] BAR/IMD domain-like
[2] SH3-domain
Pfams:
[3] Fes/CIP4, and EFC/F-BAR homology domain
[4] PB016495 (Pfam-B)
[5] PB003767 (Pfam-B)
[6] PF14604.1 (Domain)
[7] PB018735 (Pfam-B)
[8] PB006408 (Pfam-B)

(d)

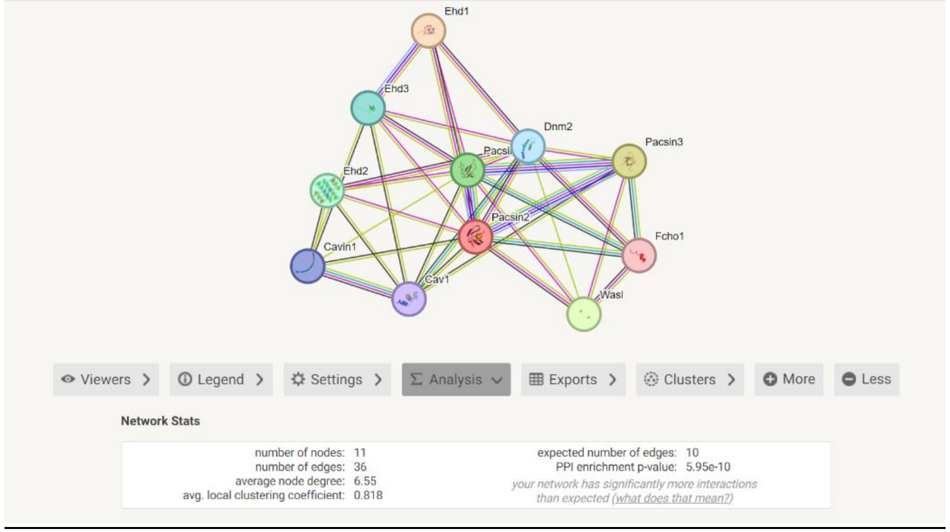
Ehd1
Ehd3
Dnm2
Pacsin3
Pacsin1
Ehd2
Pacsin2
Fcho1
Cavin1
Cav1
Wasl
Viewers
Legend
Settings
Analysis
Exports
Clusters
More
Less
Network Stats
number of nodes: 11
number of edges: 36
average node degree: 6.55
avg. local clustering coefficient: 0.818
expected number of edges: 10
PPI enrichment p-value: 5.95e-10
your network has significantly more interactions than expected (what does that mean?)

(e)

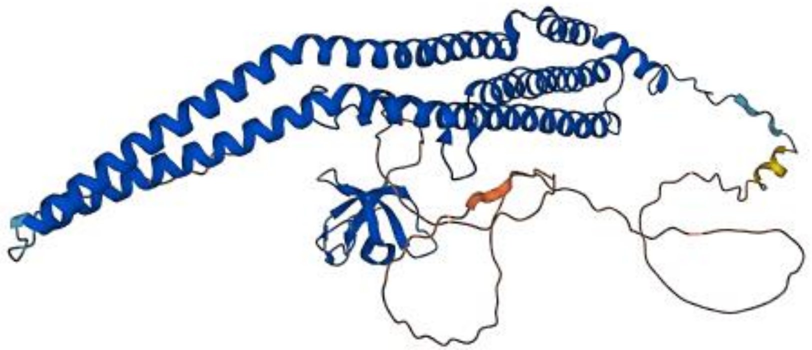

(f)

Figure S1. FuzDrop results (**a**,**b**), RIDAO results (**c**), D2P2 Results (**d**), STRING–generated PPI network (**e**), and AlphaFold Structure (**f**) for protein Pascin2 (UniProt ID: Q9WVE8).

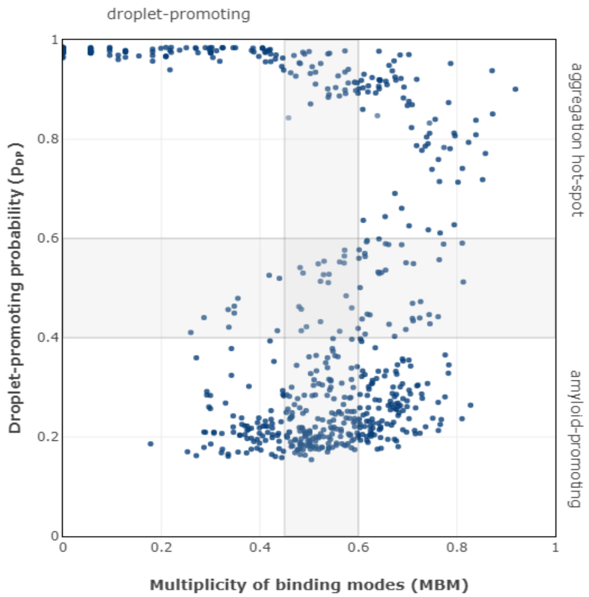

(**a**)

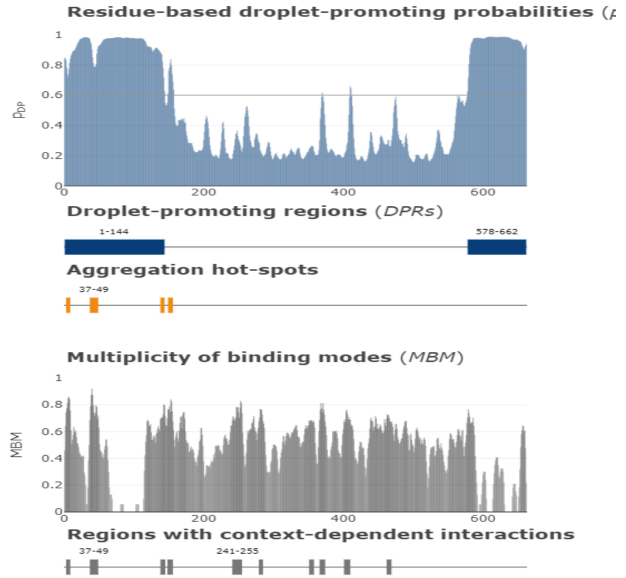

(**b**)

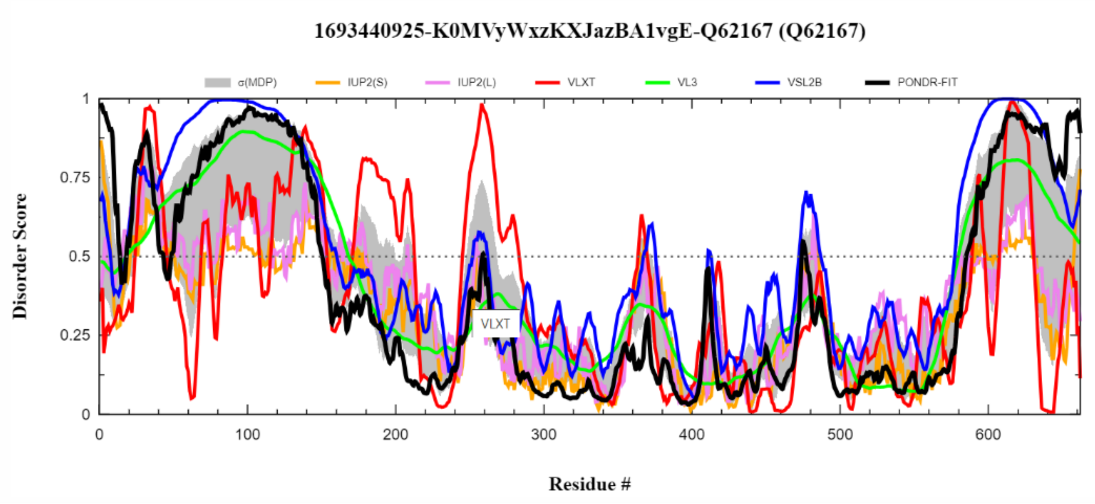

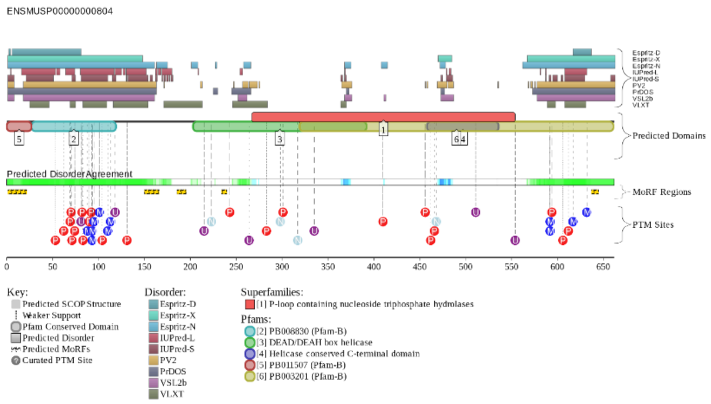

(**c**) (**d**)

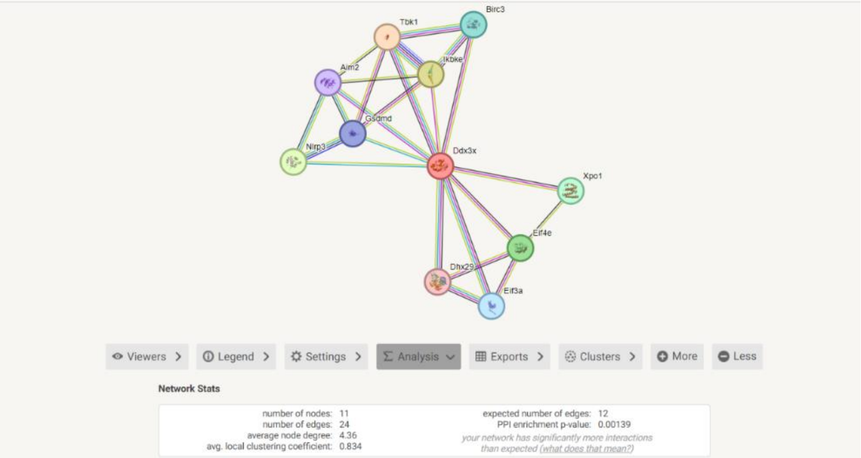

(**e**)

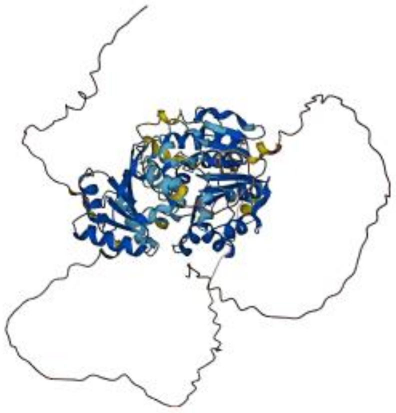

(**f**)

Figure S2. FuzDrop results (**a**,**b**), RIDAO results (**c**), D2P2 Results (**d**), STRING–generated PPI network (**e**), and AlphaFold Structure (**f**) for protein Ddx3x (UniProt ID: Q62167)**.**

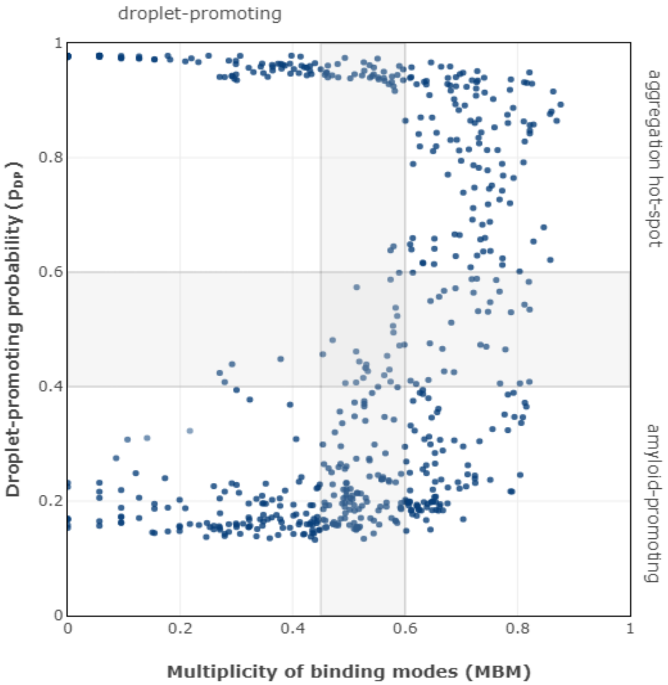
droplet-promoting
Droplet-promoting probability (pDP)
aggregation hot-spot
amyloid-promoting
Multiplicity of binding modes (MBM)
0
0.2
0.4
0.6
0.8
1

(**a**)

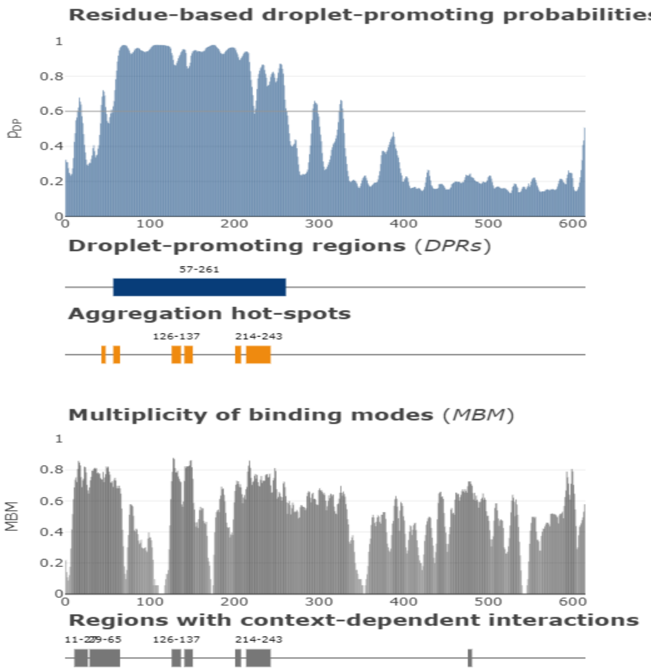
Residue-based droplet-promoting probabilities
pDP
Droplet-promoting regions (DPRs)
57-261
Aggregation hot-spots
126-137
214-243
Multiplicity of binding modes (MBM)
MBM
Regions with context-dependent interactions
126-137
214-243

(**b**)

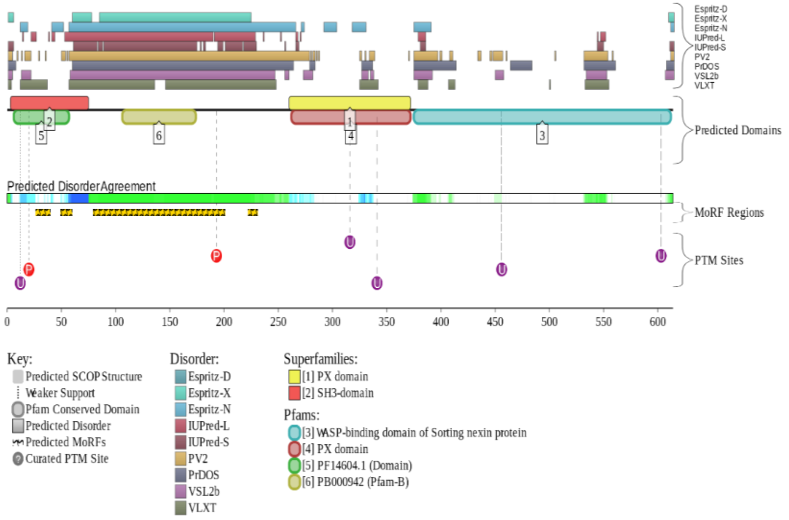

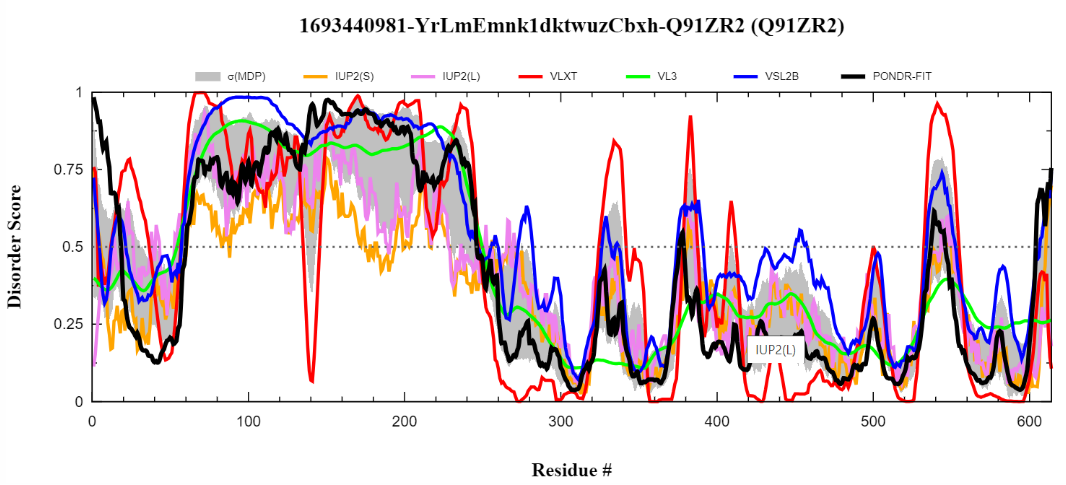

(**c**) (**d**)

Figure S3. FuzDrop (**a**,**b**), D2P2 (**c**), and RIDAO (**d**) results for protein Snx18 (UniProt ID:Q9Z1R2).

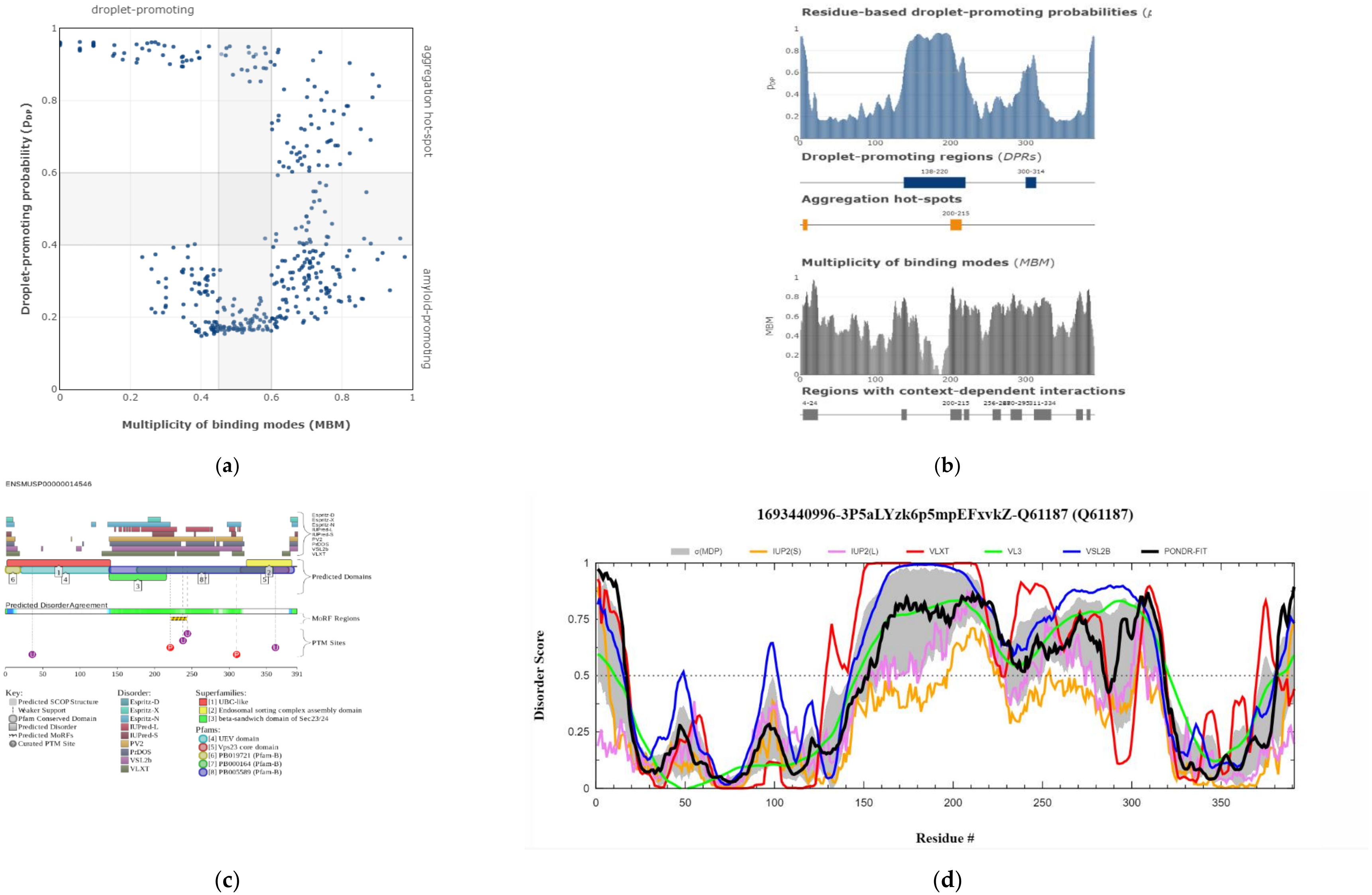

(a) (b) (c) (d)

Figure S4. FuzDrop (**a**,**b**), D2P2 (**c**), and RIDAO (**d**) results for protein Tsg101 (UniProt ID: Q61187).

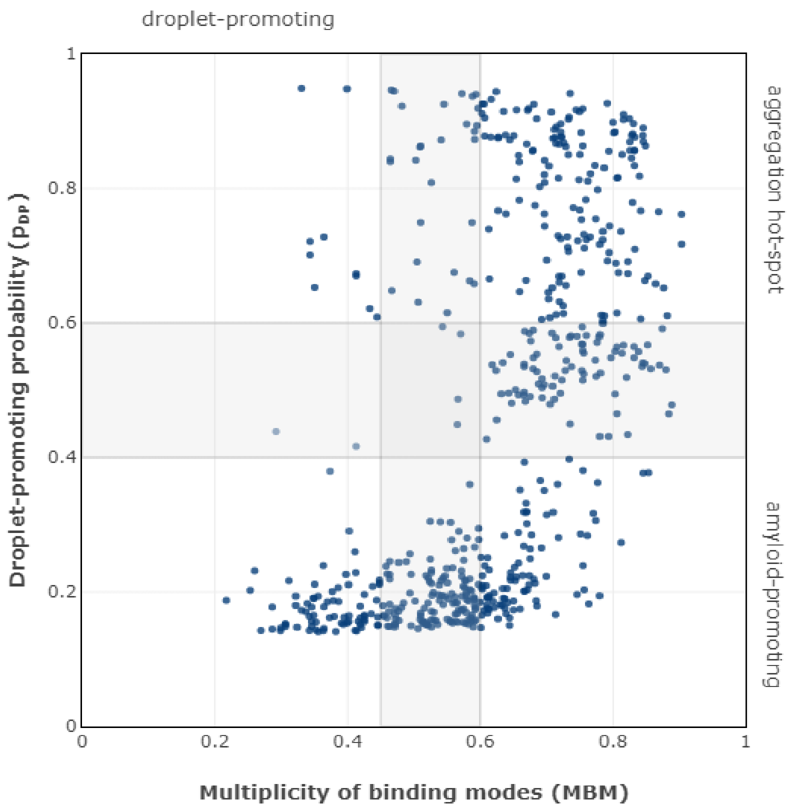
droplet-promoting
aggregation hot-spot
amyloid-promoting
Droplet-promoting probability (pDP)
Multiplicity of binding modes (MBM)

(**a**)

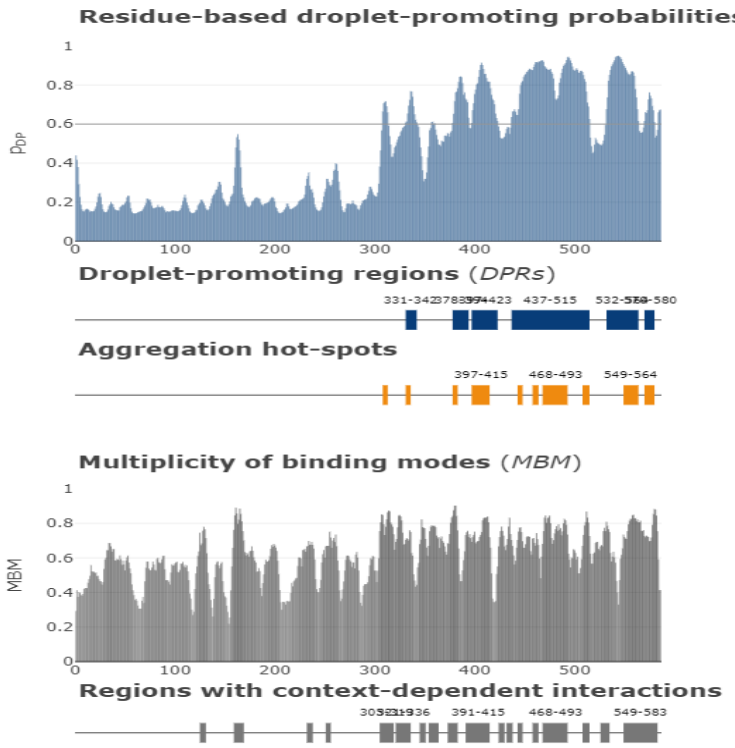
Residue-based droplet-promoting probabilities
Droplet-promoting regions (DPRs)
331-342
437-515
Aggregation hot-spots
397-415
468-493
549-564
Multiplicity of binding modes (MBM)
Regions with context-dependent interactions
391-415
468-493
549-583

(**b**)

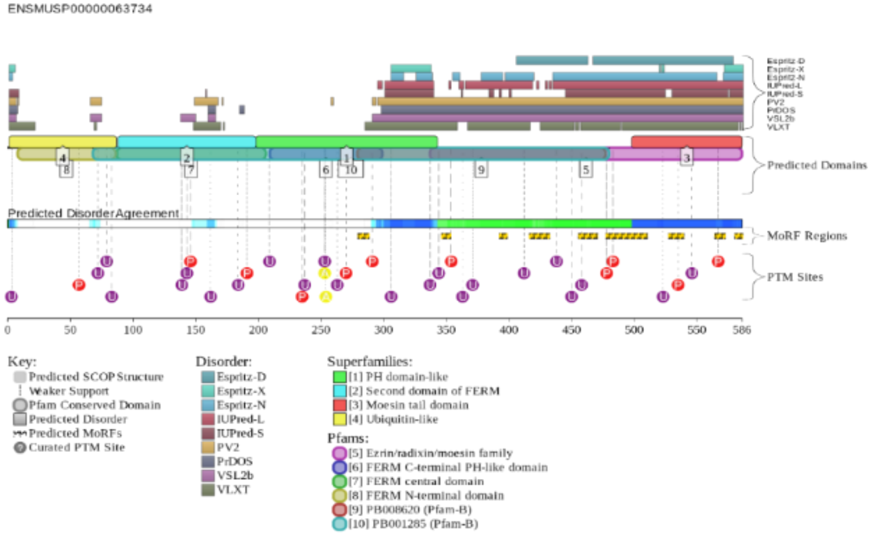

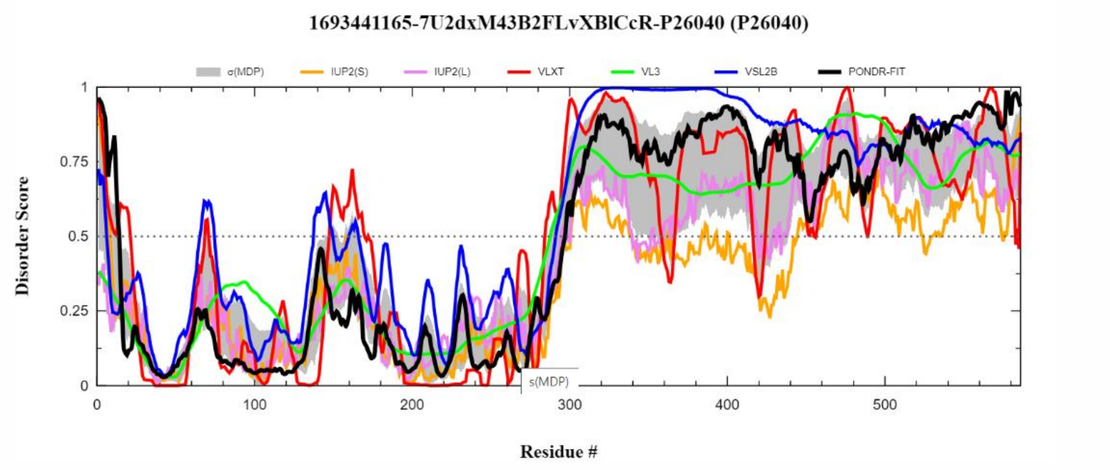

(**c**) (**d**)

Figure S5. FuzDrop (**a**,**b**), D2P2 (**c**), and RIDAO (**d**) results for protein Ezr (UniProt ID: P26040).
